# Supplementary figures and images for: Comparisons of performances of structural variants detection algorithms in solitary or combination strategy
Source: PLoS One. 2025 Feb 6;20(2):e0314982. doi: 10.1371/journal.pone.0314982 (PMC11801633; doi:10.1371/journal.pone.0314982)

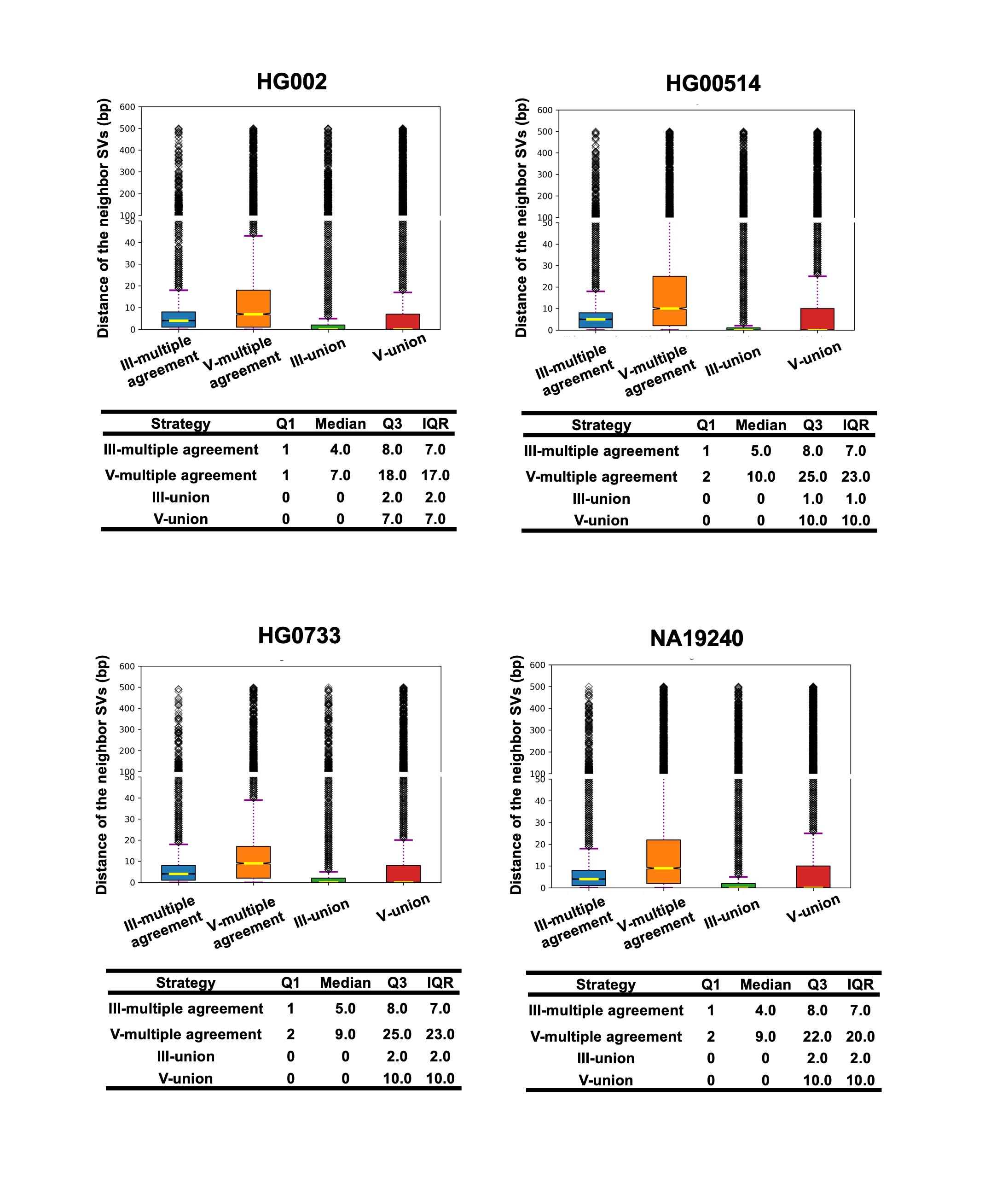

Supplement: S1 Fig — The distribution of distances between start positions in neighboring SVs across different combination strategies. IQR: Interquartile Range. (TIF) [file pone.0314982.s001.tif]

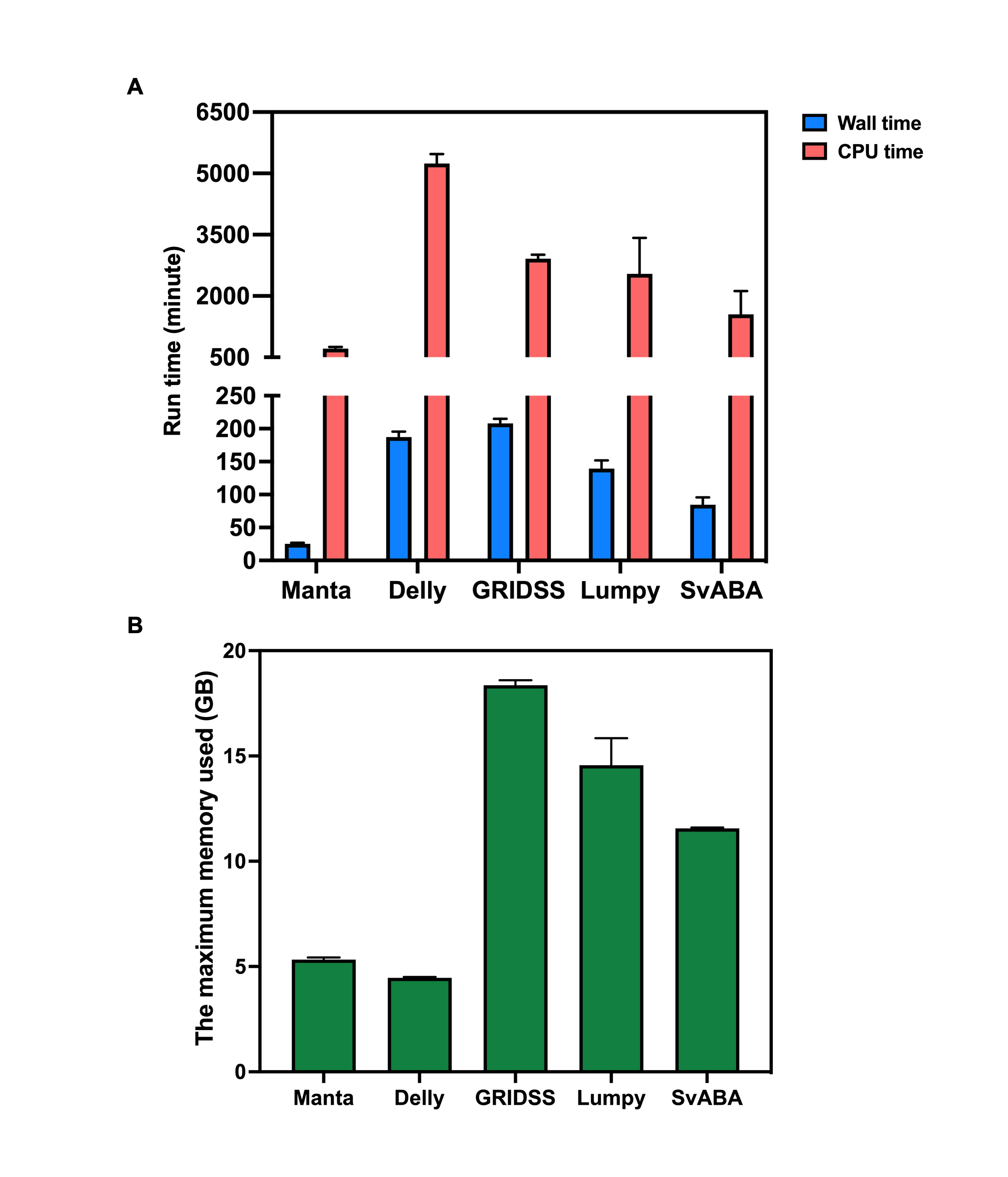

Supplement: S2 Fig — Runtime performance of each tool was tested on an isolated virtual machine, Taiwania 3, a high-performance computing platform featuring 900 compute nodes, each equipped with dual Intel Xeon Platinum 8280 processors (2.4 GHz, 28 cores per processor) and 192 GB of main memory. The system runs on CentOS 7.8 and utilizes the Slurm workload manager for resource scheduling. Taiwania 3 employs 100 Gbps InfiniBand HDR100 high-speed network connectivity, delivering a total computing power of up to 2.7 PFLOPS. We use node ngs92G, with total memory: 92 GB and total CPU cores: 14. Total CPU time indicates the overall CPU utilization, while wall time represents the real total elapsed time. Wall time can be less than the total CPU time if the process is executed efficiently, utilizing parallelization. (TIF) [file pone.0314982.s002.tif]

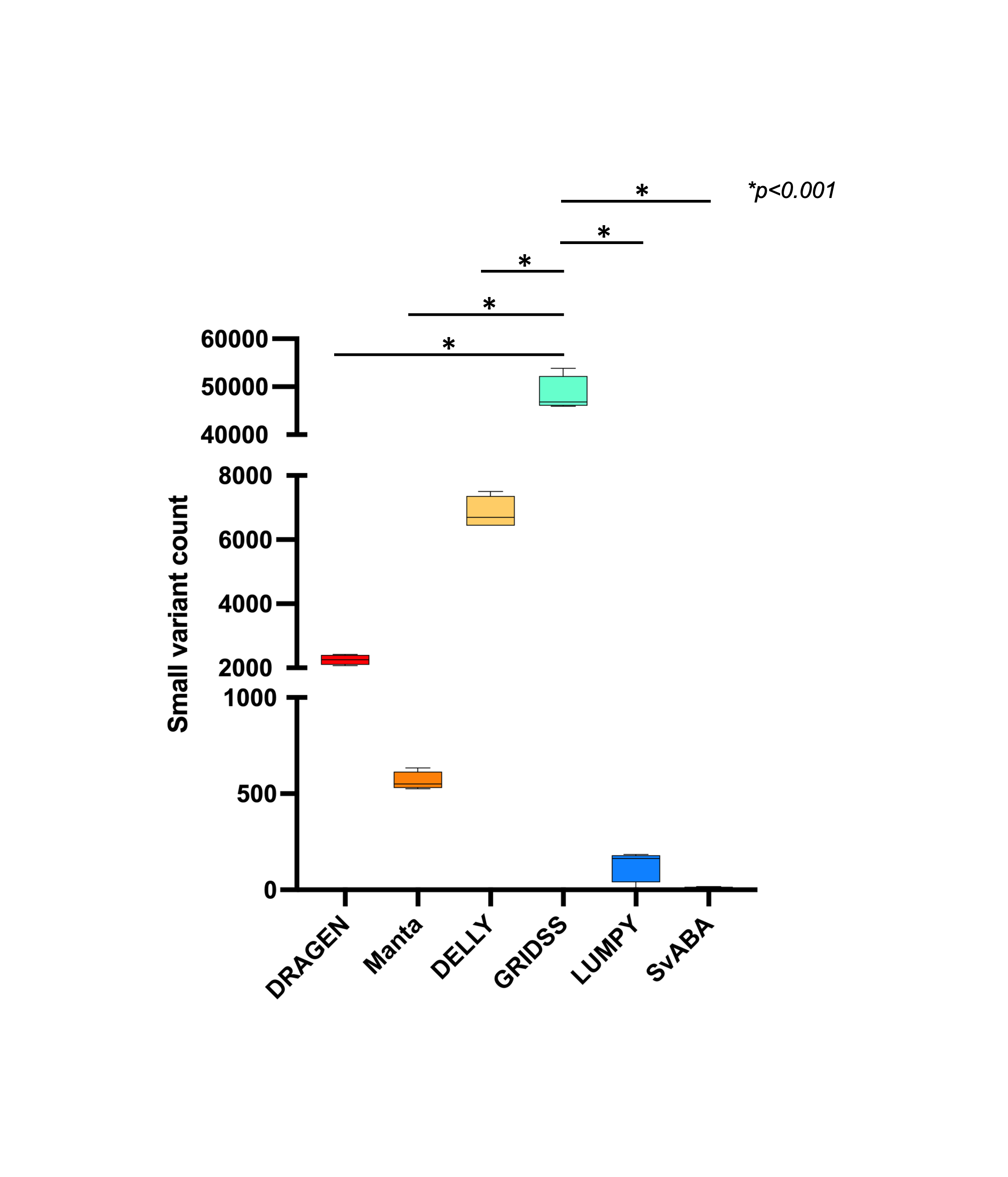

Supplement: S3 Fig — The comparative effectiveness of GRIDSS in detecting small variants compared to other SV detection tools. *p<0.0001. (TIF) [file pone.0314982.s003.tif]

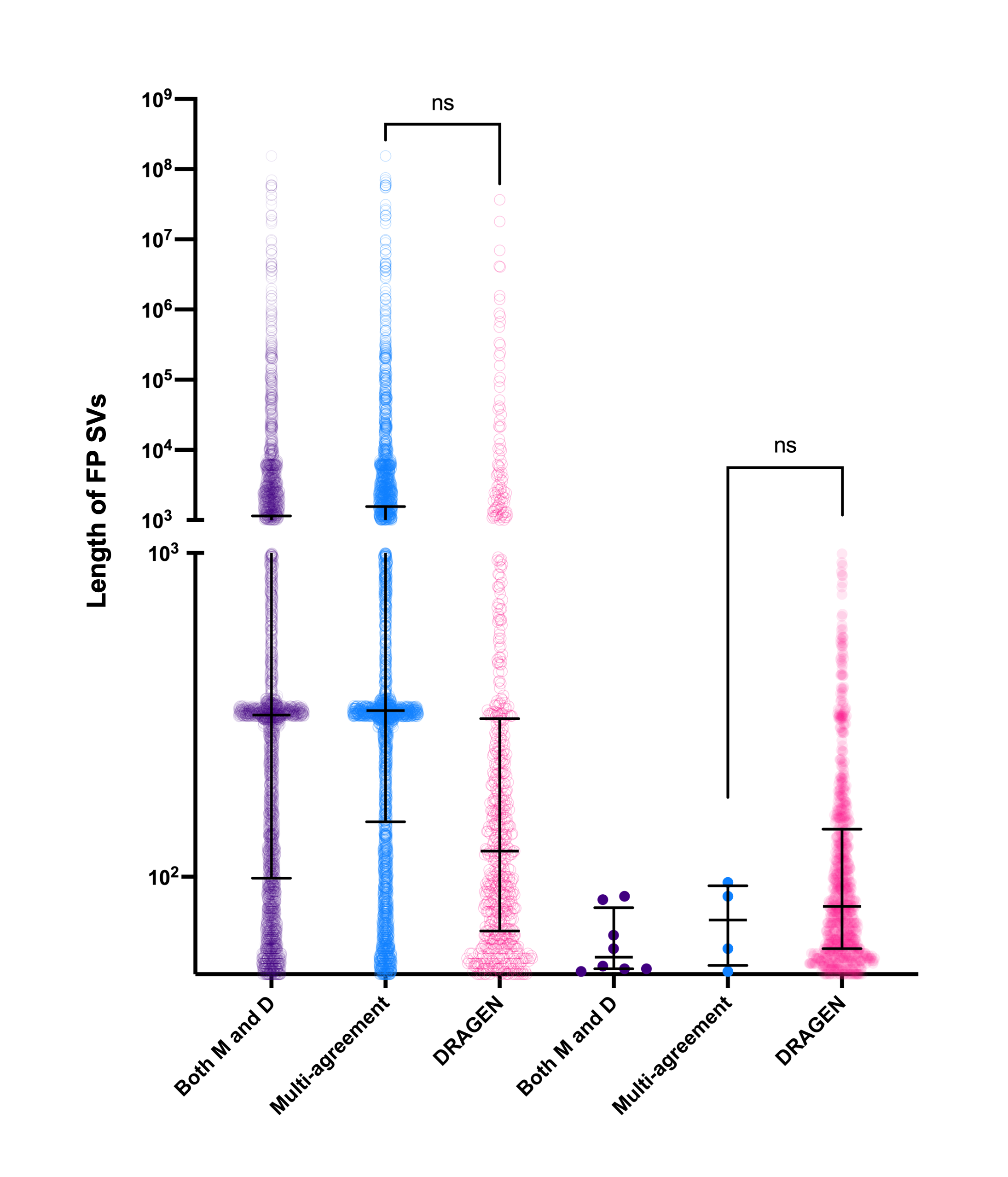

Supplement: S4 Fig — FPs identified only by the multi-agreement strategy (M), only by DRAGEN (D), and those identified by both (Both), in the HG002 dataset. The FP SV size displaying the median and interquartile range were shown. (TIF) [file pone.0314982.s004.tif]

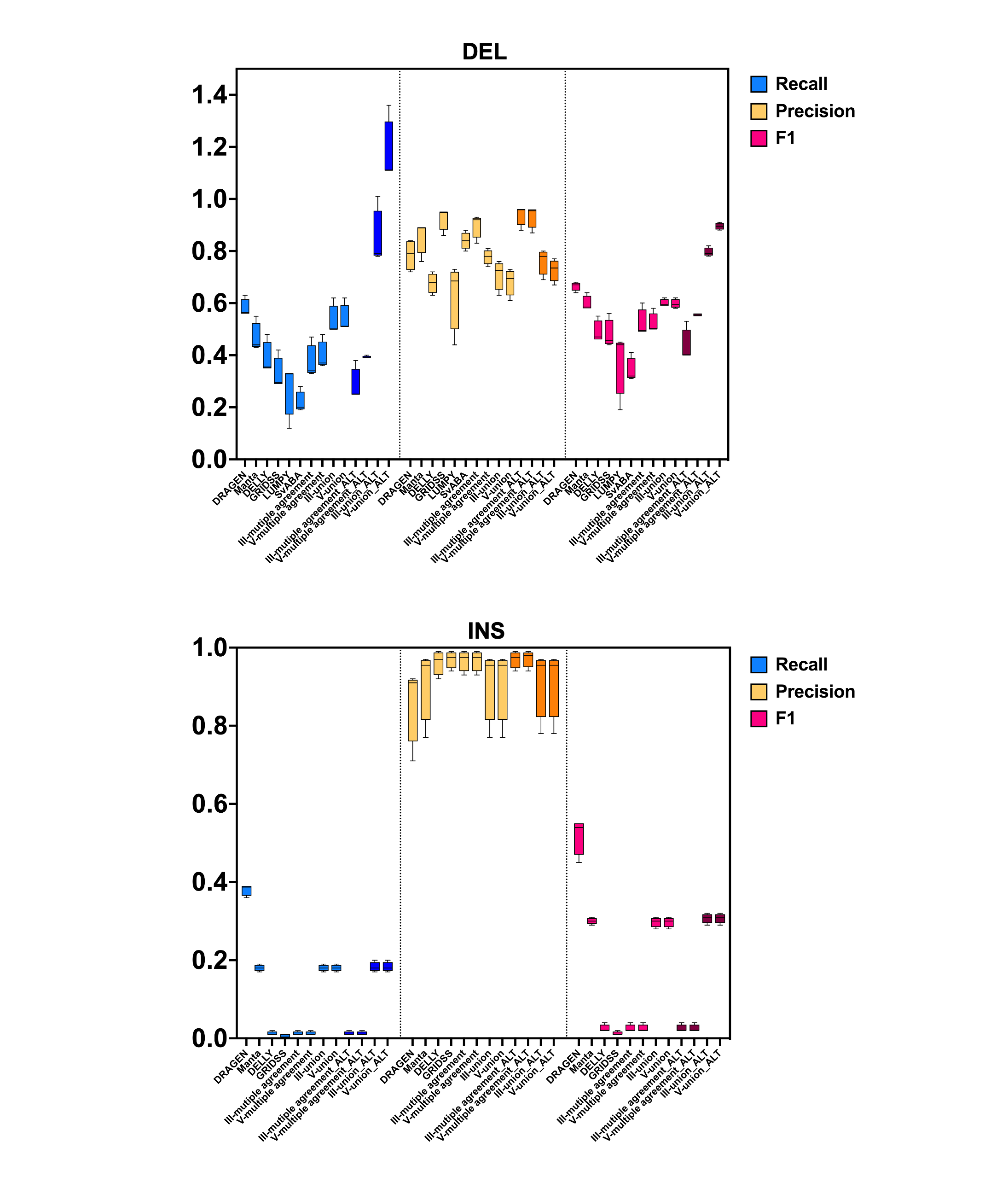

Supplement: S5 Fig — The maximum, minimum, and macro averages for recall, precision, and F1 score are calculated across all sample sets. Darker colors represent results from the ’directly merge method’. The ’neighboring SV method’ is explained in the ’Combination strategies of multiple algorithms’ section of the Methods, while the ’directly merge method’ involves merging all caller results into one VCF file before calculating the F1 score. (TIF) [file pone.0314982.s005.tif]
