# Supplementary material for: Comparisons of performances of structural variants detection algorithms in solitary or combination strategy
Source: PLoS One. 2025 Feb 6;20(2):e0314982. doi: 10.1371/journal.pone.0314982 (PMC11801633; doi:10.1371/journal.pone.0314982)
Supplement: S1 Table — (DOCX) [file pone.0314982.s006.docx]

**S1 Table. Types and sizes of all SVs detected by each individual algorithm in HG002**

| **SV callers** | **SV sizes** | **SV types** | | | | | |
| --- | --- | --- | --- | --- | --- | --- | --- |
|  |  | **DEL** | **INS** | **DUP** | | **INV** | **CXT** |
| **Manta** | [0, 50) | 40 | 518 | 1 | | 0 | - |
|  | [50, 1K) | 3,297 | 1,800 | 344 | | 85 | - |
|  | [1K, 10K) | 544 | 0 | 47 | | 55 | - |
|  | [10K, 100K) | 62 | 0 | 21 | | 31 | - |
|  | [100K, 1000K) | 19 | 0 | 15 | | 25 | - |
|  | $\geq$1000K | 13 | 0 | 14 | | 50 | - |
| Raw (Total) | 7,317 | 3,976 | 2,318 | 442 | 246 | | 335 |
| **Filtered (**$\geq$**50 bp)** | **7,758** | **3,936** | **1,800** | **441** | **246** | | **335** |
| **DELLY** | [0, 50) | 5,229 | 1,719 | 0 | 0 | | - |
|  | [50, 1K) | 2,739 | 155 | 677 | 73 | | - |
|  | [1K, 10K) | 912 | 0 | 200 | 115 | | - |
|  | [10K, 100K) | 232 | 0 | 172 | 158 | | - |
|  | [100K, 1000K) | 105 | 0 | 107 | 182 | | - |
|  | $\geq$1000K | 154 | 0 | 231 | 490 | | - |
| Raw (Total) | 13,650 | 9,371 | 1,874 | 1,387 | 1,018 | | 0 |
| **Filtered (**$\geq$**50 bp)** | **6,702** | **4,142** | **155** | **1,387** | **1,018** | | **0** |
| **GRIDSS** | [0, 50) | 24,045 | 23206 | 51 | 6 | | - |
|  | [50, 1K) | 2,097 | 90 | 514 | 92 | | - |
|  | [1K, 10K) | 527 | 0 | 36 | 60 | | - |
|  | [10K, 100K) | 43 | 0 | 9 | 16 | | - |
|  | [100K, 1000K) | 7 | 0 | 4 | 4 | | - |
|  | $\geq$1000K | 7 | 0 | 11 | 16 | | - |
| Raw (Total) | 51,056 | 26,726 | 23,296 | 628 | 194 | | 212 |
| **Filtered (**$\geq$**50 bp)** | **3,748** | **2,681** | **90** | **577** | **188** | | **212** |
| **LUMPY** | [0, 50) | 0 | 0 | 0 | 0 | | - |
|  | [50, 1K) | 219 | 0 | 147 | 57 | | - |
|  | [1K, 10K) | 933 | 0 | 304 | 93 | | - |
|  | [10K, 100K) | 238 | 0 | 190 | 126 | | - |
|  | [100K, 1000K) | 93 | 0 | 97 | 169 | | - |
|  | $\geq$1000K | 45 | 0 | 56 | 186 | | - |
| Raw (Total) | 4,656 | 1,528 | 0 | 794 | 631 | | 1,703 |
| **Filtered (**$\geq$**50 bp)** | **4,656** | **1,528** | **0** | **794** | **631** | | **1,703** |
| **SvABA** | [0, 50) | 3 | 1 | 4 | 0 | | - |
|  | [50, 1K) | 1,217 | 0 | 1,585 | 37 | | - |
|  | [1K, 10K) | 611 | 0 | 64 | 40 | | - |
|  | [10K, 100K) | 38 | 0 | 15 | 21 | | - |
|  | [100K, 1000K) | 27 | 0 | 21 | 25 | | - |
|  | $\geq$1000K | 17 | 0 | 25 | 50 | | - |
| Raw (Total) | 3,849 | 1,913 | 1 | 1,714 | 173 | | 48 |
| **Filtered (**$\geq$**50 bp)** | **3,841** | **1,910** | **0** | **1,710** | **173** | | **48** |
| **DRAGEN** | [0, 50) | 41 | 2126 | 0 | 0 | | - |
|  | [50, 1K) | 4,091 | 3,403 | 15 | 78 | | - |
|  | [1K, 10K) | 620 | 0 | 51 | 46 | | - |
|  | [10K, 100K) | 53 | 0 | 15 | 28 | | - |
|  | [100K, 1000K) | 15 | 0 | 11 | 21 | | - |
|  | $\geq$1000K | 8 | 0 | 9 | 40 | | - |
| Raw (Total) | 10,953 | 4,828 | 5,529 | 101 | 213 | | 282 |
| **Filtered (**$\geq$**50 bp)** | **10,912** | **4,787** | **5,529** | **101** | **213** | | **282** |

Raw: total number of detected structural variants (SVs); Filtered: number of detected SVs $\geq$50 bp.
