# Supplementary material for: Comparisons of performances of structural variants detection algorithms in solitary or combination strategy
Source: PLoS One. 2025 Feb 6;20(2):e0314982. doi: 10.1371/journal.pone.0314982 (PMC11801633; doi:10.1371/journal.pone.0314982)
Supplement: S2 Table — (DOCX) [file pone.0314982.s007.docx]

**S2 Table. Types and sizes of all SVs detected by each individual algorithm in HG00514**

| **SV callers** | **SV sizes** | **SV types** | | | | |
| --- | --- | --- | --- | --- | --- | --- |
|  |  | **DEL** | **INS** | **DUP** | **INV** | **CXT** |
| **Manta** | [0, 50) | 31 | 495 | 0 | 0 | - |
|  | [50, 1K) | 3,439 | 2,297 | 353 | 92 | - |
|  | [1K, 10K) | 637 | 0 | 73 | 66 | - |
|  | [10K, 100K) | 67 | 0 | 39 | 34 | - |
|  | [100K, 1000K) | 25 | 0 | 31 | 42 | - |
|  | $\geq$1000K | 15 | 0 | 24 | 67 | - |
| Raw (Total) | 8,632 | 4,214 | 2,786 | 520 | 301 | 811 |
| **Filtered (**$\geq$**50 bp)** | **8,106** | **4,183** | **2,291** | **520** | **301** | **811** |
| **DELLY** | [0, 50) | 4,977 | 1,471 | 0 | 0 | - |
|  | [50, 1K) | 2,616 | 128 | 645 | 88 | - |
|  | [1K, 10K) | 962 | 0 | 267 | 116 | - |
|  | [10K, 100K) | 343 | 0 | 300 | 133 | - |
|  | [100K, 1000K) | 240 | 0 | 251 | 221 | - |
|  | $\geq$1000K | 198 | 0 | 265 | 464 | - |
| Raw (Total) | 13,685 | 9,336 | 1,599 | 1,728 | 1,022 | 0 |
| **Filtered (**$\geq$**50 bp)** | **7,237** | **4,359** | **128** | **1,728** | **1,022** | **0** |
| **GRIDSS** | [0, 50) | 23,091 | 23,185 | 66 | 13 | - |
|  | [50, 1K) | 2,120 | 90 | 490 | 78 | - |
|  | [1K, 10K) | 559 | 0 | 42 | 63 | - |
|  | [10K, 100K) | 30 | 0 | 7 | 7 | - |
|  | [100K, 1000K) | 5 | 0 | 4 | 1 | - |
|  | $\geq$1000K | 4 | 0 | 7 | 16 | - |
| Raw (Total) | 50,444 | 25,809 | 23,275 | 616 | 178 | 566 |
| **Filtered (**$\geq$**50 bp)** | **4,089** | **2,718** | **90** | **550** | **165** | **566** |
| **LUMPY** | [0, 50) | 150 | 0 | 0 | 7 | - |
|  | [50, 1K) | 2,302 | 0 | 305 | 142 | - |
|  | [1K, 10K) | 1,073 | 0 | 391 | 110 | - |
|  | [10K, 100K) | 361 | 0 | 319 | 122 | - |
|  | [100K, 1000K) | 180 | 0 | 186 | 221 | - |
|  | $\geq$1000K | 137 | 0 | 153 | 277 | - |
| Raw (Total) | 12,245 | 4,203 | 0 | 1,354 | 879 | 5,809 |
| **Filtered (**$\geq$**50 bp)** | **12,088** | **4,053** | **0** | **1,354** | **872** | **5,809** |
| **SvABA** | [0, 50) | 3 | 0 | 8 | 0 | - |
|  | [50, 1K) | 1,113 | 0 | 1,136 | 66 | - |
|  | [1K, 10K) | 684 | 0 | 82 | 54 | - |
|  | [10K, 100K) | 68 | 0 | 47 | 18 | - |
|  | [100K, 1000K) | 80 | 0 | 70 | 65 | - |
|  | $\geq$1000K | 36 | 0 | 42 | 69 | - |
| **Raw (Total)** | **3,883** | **1,984** | **0** | **1,385** | **272** | **242** |
| **Filtered (**$\geq$**50 bp)** | **3,872** | **1,981** | **0** | **1,377** | **272** | **242** |
| **DRAGEN** | [0, 50) | 39 | 2,296 | 0 | 0 | - |
|  | [50, 1K) | 4,922 | 5,090 | 37 | 97 | **-** |
|  | [1K, 10K) | 825 | 10 | 158 | 76 | **-** |
|  | [10K, 100K) | 222 | 0 | 200 | 48 | - |
|  | [100K, 1000K) | 226 | 0 | 220 | 71 | - |
|  | $\geq$1000K | 208 | 0 | 229 | 133 | - |
| Raw (Total) | 16,072 | 6,442 | 7,396 | 844 | 425 | 965 |
| **Filtered (**$\geq$**50 bp)** | **13,737** | **6,403** | **5,100** | **844** | **425** | **965** |

Raw: total number of detected structural variants (SVs); Filtered: number of detected SVs $\geq$50 bp.
