# Supplementary material for: Comparisons of performances of structural variants detection algorithms in solitary or combination strategy
Source: PLoS One. 2025 Feb 6;20(2):e0314982. doi: 10.1371/journal.pone.0314982 (PMC11801633; doi:10.1371/journal.pone.0314982)
Supplement: S3 Table — (DOCX) [file pone.0314982.s008.docx]

**S3 Table. Types and sizes of all SVs detected by each individual algorithm in HG00733**

| **SV callers** | **SV sizes** | **SV types** | | | | |
| --- | --- | --- | --- | --- | --- | --- |
|  |  | **DEL** | **INS** | **DUP** | **INV** | **CXT** |
| **Manta** | [0, 50) | 30 | 513 | 0 | 0 | - |
|  | [50, 1K) | 3,499 | 2,312 | 376 | 91 | - |
|  | [1K, 10K) | 603 | 0 | 62 | 82 | - |
|  | [10K, 100K) | 87 | 0 | 39 | 36 | - |
|  | [100K, 1000K) | 25 | 0 | 31 | 33 | - |
|  | $\geq$1000K | 18 | 0 | 24 | 58 | - |
| Raw (Total) | 8,747 | 4,262 | 2,825 | 532 | 300 | 828 |
| **Filtered (**$\geq$**50 bp)** | **8,204** | **4,232** | **2,312** | **532** | **300** | **828** |
| **DELLY** | [0, 50) | 4,939 | 1,497 | 0 | 0 | - |
|  | [50, 1K) | 2,734 | 130 | 712 | 78 | - |
|  | [1K, 10K) | 944 | 0 | 253 | 115 | - |
|  | [10K, 100K) | 396 | 0 | 340 | 145 | - |
|  | [100K, 1000K) | 268 | 0 | 266 | 210 | - |
|  | $\geq$1000K | 232 | 0 | 263 | 450 | - |
| Raw (Total) | 13,972 | 9,513 | 1,627 | 1,834 | 998 | 0 |
| **Filtered (**$\geq$**50 bp)** | **7,536** | **4,574** | **130** | **1,834** | **998** | **0** |
| **GRIDSS** | [0, 50) | 22,605 | 23,244 | 61 | 7 | - |
|  | [50, 1K) | 2,102 | 81 | 523 | 73 | - |
|  | [1K, 10K) | 504 | 0 | 41 | 67 | - |
|  | [10K, 100K) | 42 | 0 | 4 | 9 | - |
|  | [100K, 1000K) | 3 | 0 | 5 | 3 | - |
|  | $\geq$1000K | 4 | 0 | 10 | 15 | - |
| Raw (Total) | 49,972 | 25,260 | 23,325 | 644 | 174 | 569 |
| **Filtered (**$\geq$**50 bp)** | **4,055** | **2,655** | **81** | **583** | **167** | **569** |
| **LUMPY** | [0, 50) | 165 | 0 | 0 | 4 | - |
|  | [50, 1K) | 2,351 | 0 | 325 | 145 | - |
|  | [1K, 10K) | 1,046 | 0 | 403 | 114 | - |
|  | [10K, 100K) | 423 | 0 | 406 | 129 | - |
|  | [100K, 1000K) | 184 | 0 | 194 | 207 | - |
|  | $\geq$1000K | 166 | 0 | 157 | 268 | - |
| Raw (Total) | 13,134 | 4,335 | 0 | 1,485 | 867 | 6,447 |
| **Filtered (**$\geq$**50 bp)** | **12,965** | **4,170** | **0** | **1,485** | **863** | **6,447** |
| **SvABA** | [0, 50) | 0 | 2 | 6 | 2 | - |
|  | [50, 1K) | 1,113 | 0 | 1,256 | 56 | - |
|  | [1K, 10K) | 644 | 0 | 86 | 52 | - |
|  | [10K, 100K) | 61 | 0 | 48 | 20 | - |
|  | [100K, 1000K) | 72 | 0 | 90 | 43 | - |
|  | $\geq$1000K | 54 | 0 | 43 | 60 | - |
| Raw (Total) | 3,976 | 1,944 | 2 | 1,529 | 233 | 268 |
| **Filtered (**$\geq$**50 bp)** | **3,966** | **1,944** | **0** | **1,523** | **231** | **268** |
| **DRAGEN** | [0, 50) | 41 | 2,029 | 0 | 0 | - |
|  | [50, 1K) | 5,072 | 5,312 | 27 | 76 | - |
|  | [1K, 10K) | 675 | 1 | 62 | 60 | - |
|  | [10K, 100K) | 67 | 0 | 17 | 9 | - |
|  | [100K, 1000K) | 19 | 0 | 18 | 7 | - |
|  | $\geq$1000K | 62 | 0 | 81 | 24 | - |
| Raw (Total) | 14,000 | 5,936 | 7,342 | 205 | 176 | 341 |
| **Filtered (**$\geq$**50 bp)** | **11,903** | **5,895** | **5,313** | **205** | **176** | **341** |

Raw: total number of detected structural variants (SVs); Filtered: number of detected SVs $\geq$50 bp.
