# Supplementary material for: Comparisons of performances of structural variants detection algorithms in solitary or combination strategy
Source: PLoS One. 2025 Feb 6;20(2):e0314982. doi: 10.1371/journal.pone.0314982 (PMC11801633; doi:10.1371/journal.pone.0314982)
Supplement: S4 Table — (DOCX) [file pone.0314982.s009.docx]

**S4 Table. Types and sizes of all SVs detected by each individual algorithm in NA19240**

| **SV callers** | **SV sizes** | **SV types** | | | | |
| --- | --- | --- | --- | --- | --- | --- |
|  |  | **DEL** | **INS** | **DUP** | **INV** | **CXT** |
| **Manta** | [0, 50) | 35 | 599 | 0 | 0 | - |
|  | [50, 1K) | 4,083 | 2,488 | 437 | 81 | - |
|  | [1K, 10K) | 754 | 0 | 63 | 71 | - |
|  | [10K, 100K) | 74 | 0 | 36 | 24 | - |
|  | [100K, 1000K) | 27 | 0 | 32 | 43 | - |
|  | $\geq$1000K | 17 | 0 | 15 | 65 | - |
| **Raw (Total)** | **9,867** | **4,990** | **3,087** | **583** | **284** | **923** |
| **Filtered (**$\geq$**50 bp)** | **9,233** | **4,955** | **2,488** | **583** | **284** | **923** |
| **DELLY** | [0, 50) | 5,825 | 1,681 | 0 | 0 | - |
|  | [50, 1K) | 3,161 | 134 | 674 | 92 | - |
|  | [1K, 10K) | 1107 | 0 | 275 | 123 | - |
|  | [10K, 100K) | 372 | 0 | 328 | 146 | - |
|  | [100K, 1000K) | 213 | 0 | 214 | 268 | - |
|  | $\geq$1000K | 208 | 0 | 246 | 497 | - |
| **Raw (Total)** | **15,564** | **10,886** | **1,815** | **1,737** | **1,126** | **0** |
| **Filtered (**$\geq$**50 bp)** | **8,058** | **5,061** | **134** | **1,737** | **1,126** | **0** |
| **GRIDSS** | [0, 50) | 27,252 | 26,535 | 61 | 0 | - |
|  | [50, 1K) | 2,411 | 72 | 577 | 81 | - |
|  | [1K, 10K) | 642 | 0 | 42 | 65 | - |
|  | [10K, 100K) | 36 | 0 | 6 | 4 | - |
|  | [100K, 1000K) | 6 | 0 | 3 | 2 | - |
|  | $\geq$1000K | 3 | 0 | 9 | 9 | - |
| **Raw (Total)** | **58,446** | **30,350** | **26,607** | **698** | **171** | **620** |
| **Filtered (**$\geq$**50 bp)** | **4,598** | **3,098** | **72** | **637** | **171** | **620** |
| **LUMPY** | [0, 50) | 179 | 0 | 0 | 5 | - |
|  | [50, 1K) | 2,741 | 0 | 345 | 142 | - |
|  | [1K, 10K) | 1,196 | 0 | 400 | 119 | - |
|  | [10K, 100K) | 382 | 0 | 362 | 127 | - |
|  | [100K, 1000K) | 154 | 0 | 165 | 262 | - |
|  | $\geq$1000K | 135 | 0 | 149 | 297 | - |
| **Raw (Total)** | **13,667** | **4,787** | **0** | **1,421** | **952** | **6,507** |
| **Filtered (**$\geq$**50 bp)** | **13,483** | **4,608** | **0** | **1,421** | **947** | **6,507** |
| **SvABA** | [0, 50) | 5 | 1 | 10 | 0 | - |
|  | [50, 1K) | 1,331 | 0 | 1,161 | 70 | - |
|  | [1K, 10K) | 802 | 0 | 73 | 40 | - |
|  | [10K, 100K) | 66 | 0 | 36 | 22 | - |
|  | [100K, 1000K) | 49 | 0 | 68 | 55 | - |
|  | $\geq$1000K | 35 | 0 | 31 | 72 | - |
| **Raw (Total)** | **4,213** | **2,288** | **1** | **1,379** | **259** | **286** |
| **Filtered (**$\geq$**50 bp)** | **4,197** | **2,283** | **0** | **1,369** | **259** | **286** |
| **DRAGEN** | [0, 50) | 49 | 2,375 | 0 | 0 | - |
|  | [50, 1K) | 5,905 | 6,144 | 24 | 74 | - |
|  | [1K, 10K) | 840 | 0 | 58 | 57 | - |
|  | [10K, 100K) | 55 | 0 | 20 | 5 | - |
|  | [100K, 1000K) | 21 | 0 | 19 | 11 | - |
|  | $\geq$1000K | 76 | 0 | 88 | 18 | - |
| **Raw (Total)** | **16,188** | **6,946** | **8,501** | **209** | **165** | **367** |
| **Filtered (**$\geq$**50 bp)** | **13,764** | **6,897** | **6,126** | **209** | **165** | **367** |

Raw: total number of detected structural variants (SVs); Filtered: number of detected SVs $\geq$50 bp.
