# Supplementary material for: Comparisons of performances of structural variants detection algorithms in solitary or combination strategy
Source: PLoS One. 2025 Feb 6;20(2):e0314982. doi: 10.1371/journal.pone.0314982 (PMC11801633; doi:10.1371/journal.pone.0314982)
Supplement: S5 Table — (DOCX) [file pone.0314982.s010.docx]

**S5 Table. Distribution of SVs in truth sets**

| SV truth sets | SV sizes | SV types | |
| --- | --- | --- | --- |
|  |  | DEL | INS |
| GIAB v.6 Tier1 (HG002) | [50, 1K) | **4,846** | **6,341** |
|  | [1K, 10K) | **578** | **909** |
|  | [10K, 100K) | 39 | 29 |
|  | [100K, 1000K) | 1 | 2 |
| Raw (Total) | 12,745 | **5,464** | **7,281** |
| HGSVC2  (HG00514) | [50, 1K) | **7,604** | **10,906** |
|  | [1K, 10K) | 867 | 1,385 |
|  | [10K, 100K) | 55 | 42 |
|  | [100K, 1000K) | 3 | 0 |
| Raw (Total) | 20,862 | 8,529 | 12,333 |
| HGSVC2  (HG00733) | [50, 1K) | **7,799** | **11,064** |
|  | [1K, 10K) | **832** | **1,396** |
|  | [10K, 100K) | 61 | 35 |
|  | [100K, 1000K) | 2 | 0 |
| Raw (Total) | 21,189 | 8,694 | 12,495 |
| HGSVC2  (NA19240) | [50, 1K) | **9,112** | **12,612** |
|  | [1K, 10K) | **986** | **1,502** |
|  | [10K, 100K) | 62 | 39 |
|  | [100K, 1000K) | 5 | 0 |
| Raw (Total) | 24,318 | 10,165 | 14,153 |

Raw: total number of SVs in truth set.
