# Supplementary material for: Comparisons of performances of structural variants detection algorithms in solitary or combination strategy
Source: PLoS One. 2025 Feb 6;20(2):e0314982. doi: 10.1371/journal.pone.0314982 (PMC11801633; doi:10.1371/journal.pone.0314982)
Supplement: S6 Table — (DOCX) [file pone.0314982.s011.docx]

**S6 Table. Count of neighbor SVs.**

| **HG002** | | |
| --- | --- | --- |
|  | **3-SV callers** | **5-SV callers** |
| Total SV | 16,660 | 23,406 |
| Total Neighbor SV | 9,771 | 11,255 |
| Neighbor SV (include 1 member variant) | **5,762** | **5,746** |
| Neighbor SV (include 2 member variants) | **1,535** | **1,964** |
| Neighbor SV (include 3 member variants) | **2,383** | **1,585** |
| Neighbor SV (include 4 member variants) | 58 | **1,277** |
| Neighbor SV (include 5 member variants) | 13 | **589** |
| Neighbor SV (include >5 member variants) | 20 | 94 |
| Multiple agreement (≥2 callers detected the variants) | 3,789 | 5,280 |
| Union (≥1 callers detected the variants) | 9,771 | 9,771 |
|  | | |
| **HG00514** | | |
|  | **3-SV callers** | **5-SV callers** |
| Total SV | 18,055 | 27,964 |
| Total Neighbor SV | 11,164 | 12,498 |
| Neighbor SV (include 1 member variant) | **7,016** | **6,251** |
| Neighbor SV (include 2 member variants) | **1,680** | **2,010** |
| Neighbor SV (include 3 member variants) | **2,386** | **1,253** |
| Neighbor SV (include 4 member variants) | 56 | **1,432** |
| Neighbor SV (include 5 member variants) | 15 | **1,409** |
| Neighbor SV (include >5 member variants) | 11 | 143 |
| Multiple agreement (≥2 callers detected the variants) | 3,858 | 6,034 |
| Union (≥1 callers detected the variants) | 11,164 | 12,498 |
|  | | |
| **HG00733** | | |
|  | **3-SV callers** | **5-SV callers** |
| Total SV | 15,383 | 22,975 |
| Total Neighbor SV | 9,252 | 10,382 |
| Neighbor SV (include 1 member variant) | **5,509** | **5,476** |
| Neighbor SV (include 2 member variants) | **1,429** | **1,324** |
| Neighbor SV (include 3 member variants) | **2,259** | **966** |
| Neighbor SV (include 4 member variants) | 40 | **1,267** |
| Neighbor SV (include 5 member variants) | 11 | **1,271** |
| Neighbor SV (include >5 member variants) | 4 | 78 |
| Multiple l agreement (≥2 callers detected the variants) | 3,640 | 4,756 |
| Union (≥1 callers detected the variants) | 9,252 | 10,382 |
|  | | |
| **NA19240** | | |
|  | **3-SV callers** | **5-SV callers** |
| Total SV | 18,398 | 28,614 |
| Total Neighbor SV | 11,454 | 12,944 |
| Neighbor SV (include 1 member variant) | **7,210** | **6,531** |
| Neighbor SV (include 2 member variants) | **1,816** | **2,110** |
| Neighbor SV (include 3 member variants) | **2,356** | **1,320** |
| Neighbor SV (include 4 member variants) | 46 | **1,474** |
| Neighbor SV (include 5 member variants) | 16 | **1,372** |
| Neighbor SV (include >5 member variants) | 10 | 137 |
| Multiple agreement (≥2 callers detected the variants) | 3,922 | 6,154 |
| Union (≥1 callers detected the variants) | 11,454 | 12,944 |
