# Supplementary material for: Comparisons of performances of structural variants detection algorithms in solitary or combination strategy
Source: PLoS One. 2025 Feb 6;20(2):e0314982. doi: 10.1371/journal.pone.0314982 (PMC11801633; doi:10.1371/journal.pone.0314982)
Supplement: S7 Table — (DOCX) [file pone.0314982.s012.docx]

**S7 Table. Distribution of SVs detected by different combination strategies.**

| **HG002** | | | | | |
| --- | --- | --- | --- | --- | --- |
| **Strategy** | **Total** | **DEL** | **INS** | **DUP** | **INV** |
| DRAGEN | 8,504 | 4,787 | 3,403 | 101 | 213 |
| III-multiple agreement | 3,789 | 3,087 | 162 | 382 | 158 |
| V-multiple agreement | 5,280 | 3,565 | 162 | 1,079 | 474 |
| III-union | 9,771 | 5,353 | 1,799 | 1,786 | 833 |
| V-union | 11,255 | 5,605 | 1,799 | 2,933 | 918 |
| **HG00514** | | | | | |
| **Strategy** | **Total** | **DEL** | **INS** | **DUP** | **INV** |
| DRAGEN | 12,772 | 6,403 | 5,100 | 844 | 425 |
| III-multiple agreement | 3,858 | 3,125 | 132 | 427 | 174 |
| V-multiple agreement | 6,034 | 3,988 | 132 | 1,321 | 593 |
| III-union | 11,164 | 5,842 | 2,277 | 2,140 | 905 |
| V-union | 12,498 | 6,234 | 2,277 | 2,964 | 1,023 |
| **HG00733** | | | | | |
| **Strategy** | **Total** | **DEL** | **INS** | **DUP** | **INV** |
| DRAGEN | 11,589 | 5,895 | 5,313 | 205 | 176 |
| III-multiple agreement | 3,922 | 3,154 | 136 | 448 | 184 |
| V-multiple agreement | 6,154 | 4,043 | 136 | 1,422 | 553 |
| III-union | 11,454 | 6,013 | 2,293 | 2,251 | 897 |
| V-union | 12,944 | 6,426 | 2,293 | 3,195 | 1,030 |
| **NA19240** | | | | | |
| **Strategy** | **Total** | **DEL** | **INS** | **DUP** | **INV** |
| DRAGEN | 13,415 | 6,897 | 6,144 | 209 | 165 |
| III-multiple agreement | 4,449 | 3,694 | 135 | 457 | 163 |
| V-multiple agreement | 6,767 | 4,594 | 135 | 1,404 | 634 |
| III-union | 12,461 | 6,751 | 2,481 | 2,257 | 972 |
| V-union | 13,755 | 7,100 | 2,481 | 3,088 | 1,086 |
