# Supplementary material for: Comparisons of performances of structural variants detection algorithms in solitary or combination strategy
Source: PLoS One. 2025 Feb 6;20(2):e0314982. doi: 10.1371/journal.pone.0314982 (PMC11801633; doi:10.1371/journal.pone.0314982)
Supplement: S1 File — (DOCX) [file pone.0314982.s013.docx]

**S1_file Example of the refinement process of “duplicated records.”**

In our study, we encountered “duplicated records” in the output files generated by several structural variant (SV) callers, including GRIDSS, SvABA, LUMPY, and Manta. These duplications typically arose when different records represented the same SV variant, with one record indicating the start position and another indicating the end position. Below is an example illustrating this process using GRIDSS output:

For example, in GRIDSS, there are two records: chr4:66740908 gridss75fb_3337o and chr4:66740925 gridss75fb_3337h represent for one structural variant.


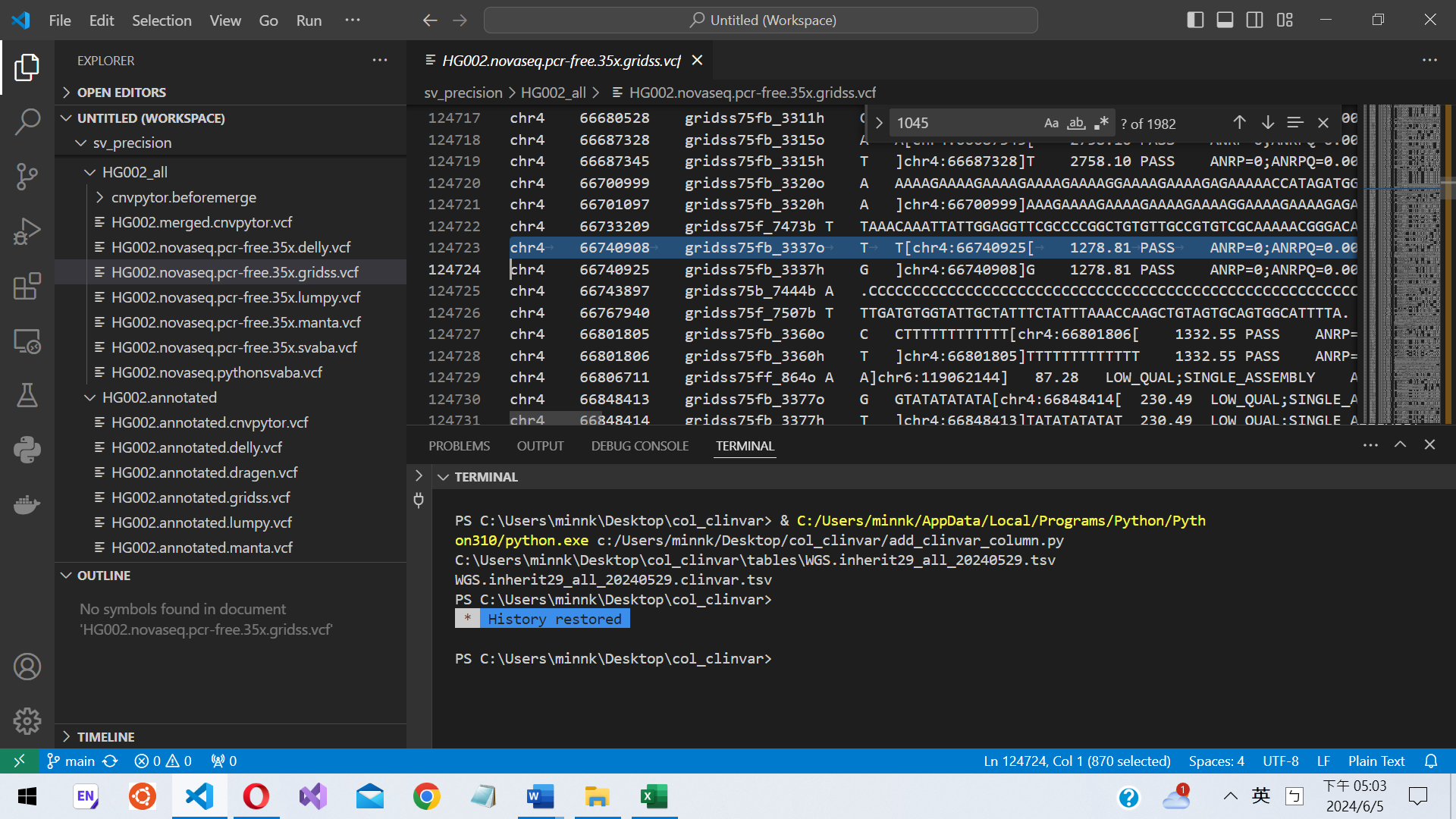
1. Raw data: two records for one variant

2. Annotation with R Package: two records for one variant, both are annotated as
 “SIMPLE_TYPE=DEL;SVLEN=-16”


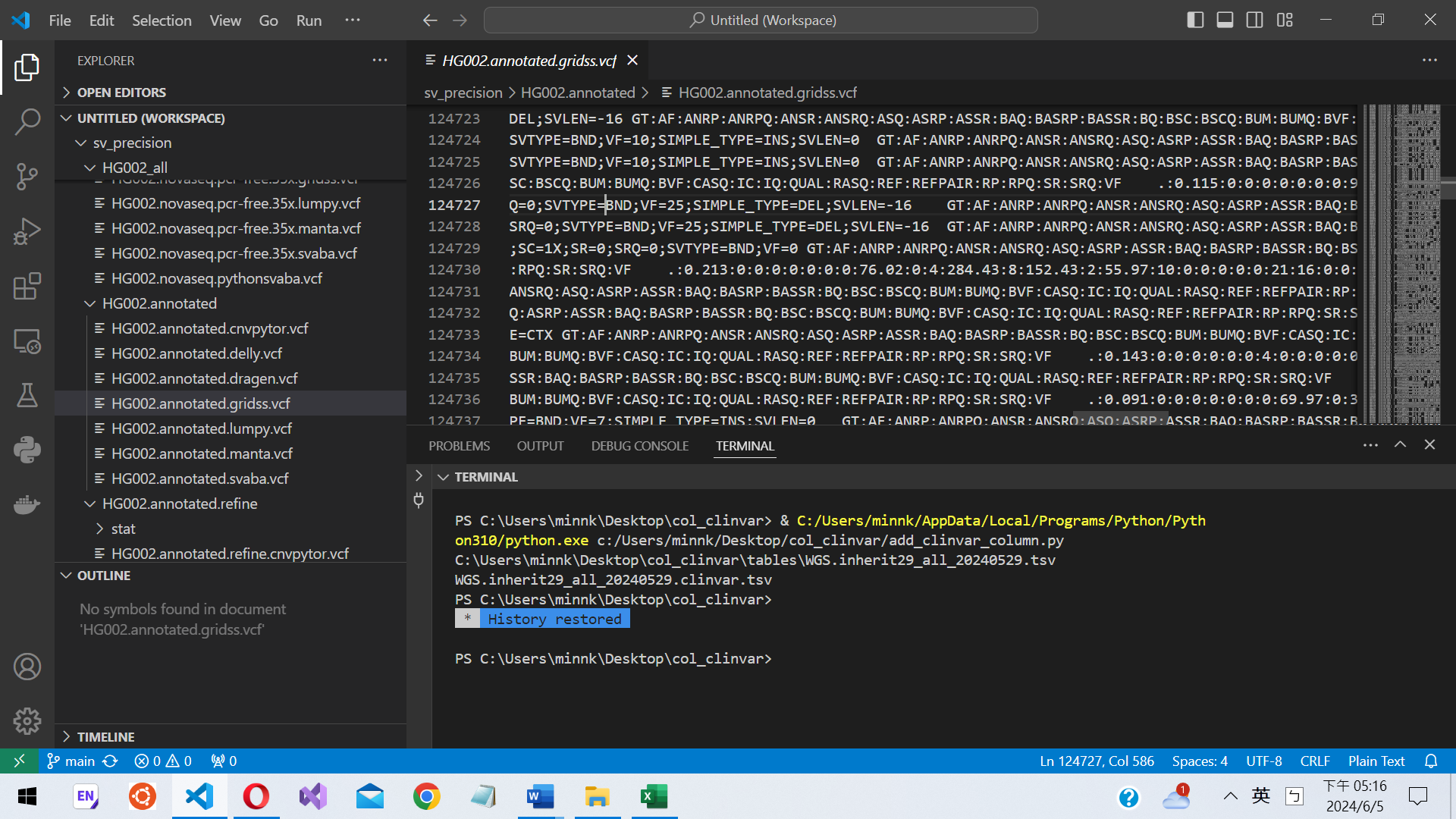

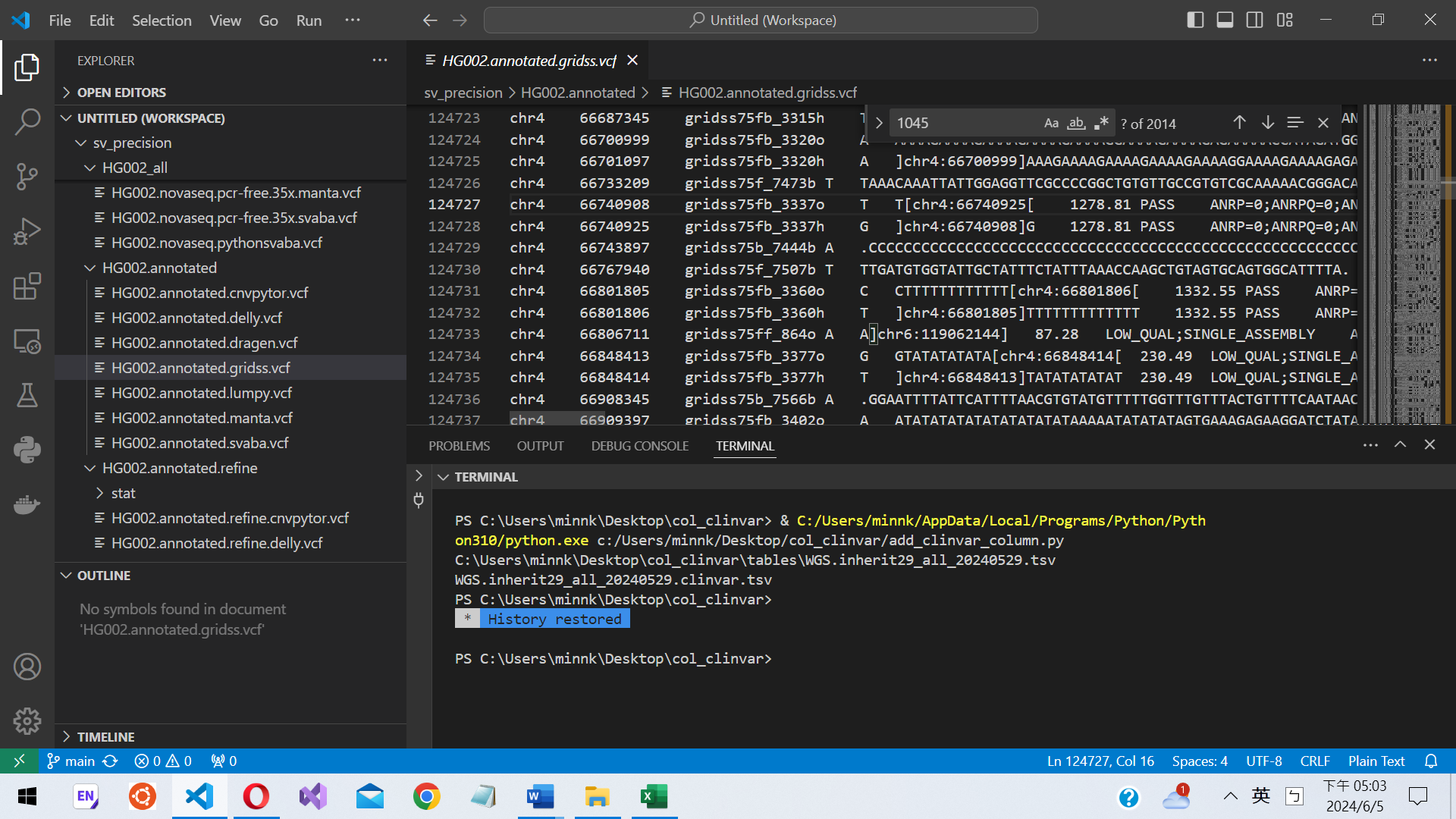


**3. Refinement Process:** To ensure each SV variant is represented uniquely in our
 dataset, we applied the following refinement steps:

1. **Identification:** We identified “duplicated records” by comparing genomic coordinates and variant types.
2. **Selection Criteria:** We removed the record that represented the end position of the SV.
3. **Implementation:** The refinement process was applied consistently across GRIDSS, SvABA, LUMPY, and Manta outputs to eliminate “duplicated records”.
4.
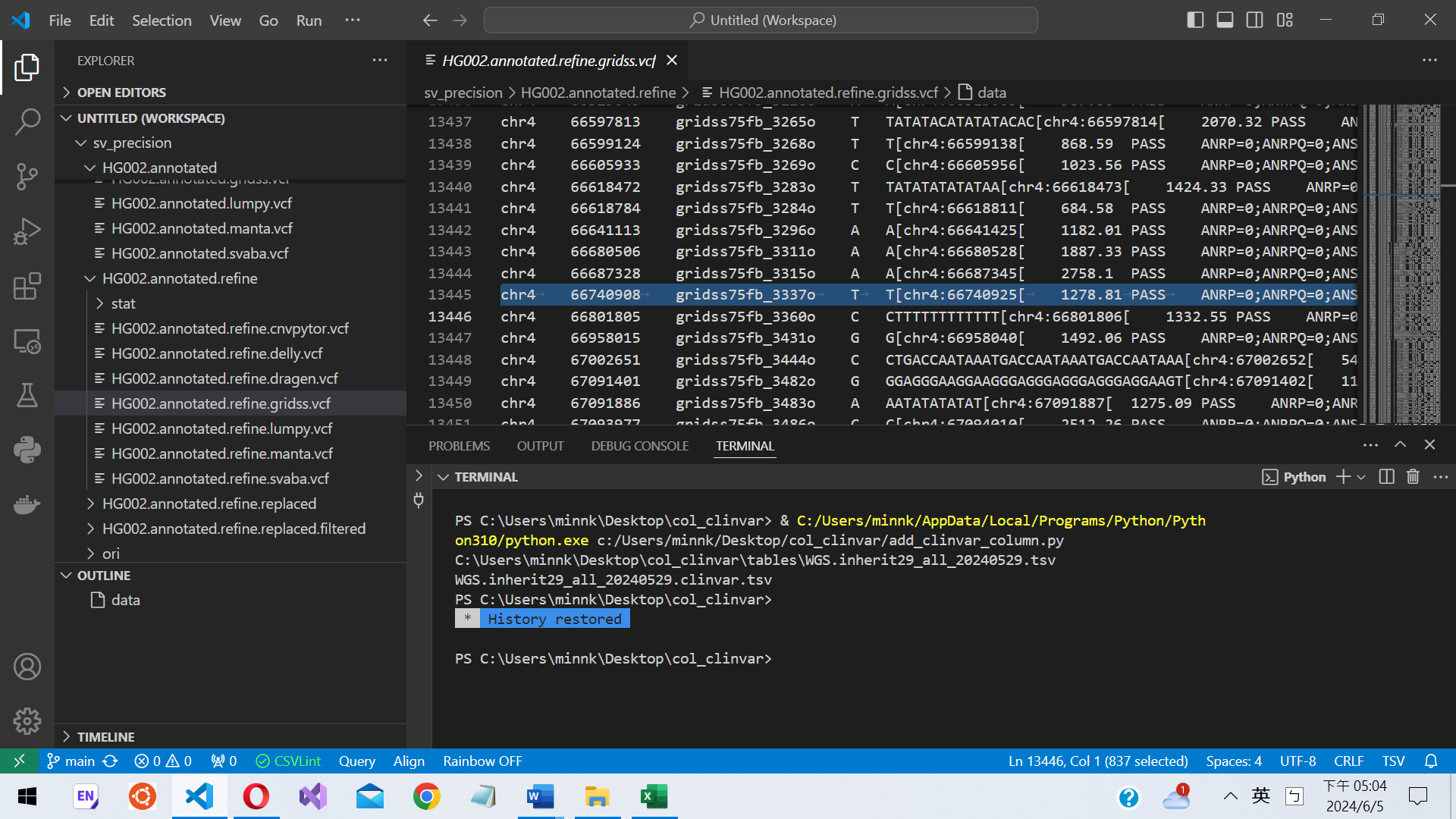
**Exception:** DELLY outputs did not exhibit “duplicated records”, and therefore, no removal was necessary for DELLY.

By implementing these steps, we ensured that our dataset contained accurate and non-redundant information for each structural variant detected by the SV callers used in our study.
