# Supplementary material for: Comparisons of performances of structural variants detection algorithms in solitary or combination strategy
Source: PLoS One. 2025 Feb 6;20(2):e0314982. doi: 10.1371/journal.pone.0314982 (PMC11801633; doi:10.1371/journal.pone.0314982)
Supplement: S2 File — (DOCX) [file pone.0314982.s014.docx]

**S2_file Combination of neighbor SV from multiple callers.**

| **Combinaiton of Multiple Callers** | |
| --- | --- |
| **Multiple-agreement** | **Union** |
| Meet all of the following criteria:   1. 49 $<$SVLEN and 2. “Start position” +/- 500 base pairs and 3. Same SV type 4. Callers $\geq2$ | Meet all of the following criteria:   1. 49 $<$SVLEN and 2. “Start position” +/- 500 base pairs and 3. Same SV type 4. Callers $\geq1$ |
| III: Manta, DELLY, GRIDSS  V: Manta, DELLY, GRIDSS, LUMPY, SvABA | III: Manta, DELLY, GRIDSS  V: Manta, DELLY, GRIDSS, LUMPY, SvABA |
