# Supplementary material for: Comparisons of performances of structural variants detection algorithms in solitary or combination strategy
Source: PLoS One. 2025 Feb 6;20(2):e0314982. doi: 10.1371/journal.pone.0314982 (PMC11801633; doi:10.1371/journal.pone.0314982)
Supplement: Attachment — Submitted filename: point-by-point response letter to Plos One.docx [file pone.0314982.s015.docx]

Submission ID PONE-D-24-11846

**Comparisons of performances of Structural Variants Detection Algorithms in Solitary or Combination Strategy**

Point-by-point Response to the Editor and Reviewers

We greatly thank the editor and reviewers for the thoughtful comments of our manuscript. Following with all your kind suggestions, we have made improvement to our manuscript. The detailed point-by-point response to the comments are listed on the following pages. All the changes in revised manuscript are indicated by red color, and we also provide a clear version of our manuscript in the submission package. We really hope the revision will make the paper be more suitable for publication in ***PLOS ONE*** and look forward to hear from you in the near future. Your kind consideration will be highly appreciated!

**Editor**

*Comments to the Author*

Thank you for submitting your manuscript to PLOS ONE. After careful consideration, we feel that it has merit but does not fully meet PLOS ONE’s publication criteria as it currently stands. Therefore, we invite you to submit a revised version of the manuscript that addresses the points raised during the review process.

Journal Requirements:

When submitting your revision, we need you to address these additional requirements.

1. Please ensure that your manuscript meets PLOS ONE's style requirements, including those for file naming. The PLOS ONE style templates can be found at https://journals.plos.org/plosone/s/file?id=wjVg/PLOSOne_formatting_sample_main_body.pdf and https://journals.plos.org/plosone/s/file?id=ba62/PLOSOne_formatting_sample_title_ authors_ affiliations.pdf

**Response:**

Thank you for your kind comment! Following your helpful reminder, we have amended our manuscript and figures to make sure that our manuscript meets PLOS ONE's style requirements, including those for file naming. We appreciate your guidance in this matter.

2. We note that the grant information you provided in the ‘Funding Information’ and ‘Financial Disclosure’ sections do not match.

When you resubmit, please ensure that you provide the correct grant numbers for the awards you received for your study in the ‘Funding Information’ section.

**Response:**

We appreciate your kind reminder! we have made corrections to the grant information. The correct grant numbers for our study are now provided in the ‘Funding Information’ and ‘Financial Disclosure’ sections. We apologize for this point and thanks again for your kind reminder!

**Funding:**

*This work was funded by the National Science and Technology Council of Taiwan (https://www.nstc.gov.tw), under grant 111-2314-B-002 -194 -MY3 and 111-2314-B-002 -243 -MY3 to Dr. Pei-Lung Chen, the corresponding author of this study.* *There was no additional external funding received for this study. The funder had no role in study design, data collection and analysis, decision to publish, or preparation of the manuscript.*

3. In the online submission form, you indicated that "For any further requests and questions, the data used and/or analyzed during the current study are available from the corresponding author upon reasonable request." All PLOS journals now require all data underlying the findings described in their manuscript to be freely available to other researchers, either 1. In a public repository, 2. Within the manuscript itself, or 3. Uploaded as supplementary information. This policy applies to all data except where public deposition would breach compliance with the protocol approved by your research ethics board. If your data cannot be made publicly available for ethical or legal reasons (e.g., public availability would compromise patient privacy), please explain your reasons on resubmission and your exemption request will be escalated for approval.

**Response:**

Thank you for your kind reminder of the data availability requirements. In compliance with PLOS publication guidelines, we have submitted all of the data used in this study within the manuscript and its supplementary information files. The coding during the current study is available in the Github repository (https://github.com/minn333/combination_sv). Thanks again for your reminder!

4. When completing the data availability statement of the submission form, you indicated that you will make your data available on acceptance. We strongly recommend all authors decide on a data sharing plan before acceptance, as the process can be lengthy and hold up publication timelines. Please note that, though access restrictions are acceptable now, your entire data will need to be made freely accessible if your manuscript is accepted for publication. This policy applies to all data except where public deposition would breach compliance with the protocol approved by your research ethics board. If you are unable to adhere to our open data policy, please kindly revise your statement to explain your reasoning and we will seek the editor's input on an exemption. Please be assured that, once you have provided your new statement, the assessment of your exemption will not hold up the peer review process.

**Response:**

Thank you for your reminder! We respect PLOS's open data policy. All data utilized in this research are either presented in the results or have been submitted as supplementary material accompanying our submission to ensure that all data used in this study are freely accessible. Once more, thank you for your advice.

5. Please include your full ethics statement in the ‘Methods’ section of your manuscript file. In your statement, please include the full name of the IRB or ethics committee who approved or waived your study, as well as whether or not you obtained informed written or verbal consent. If consent was waived for your study, please include this information in your statement as well.

**Response:**

Thank you for your important reminder! We have included the full ethics statement in the 'Methods' section of our manuscript. Since our study used publicly available existing data and did not involve any additional human subjects, no further ethics approval or informed consent was required. And thanks again for your kind remind!

***Page 10:***

**Methods**

**Ethics approval and consent to participate**

*This research project solely utilizes public datasets. The whole-genome paired-end short reads of HG002 were acquired from the National Institute of Standards and Technology (NIST) website (https://www.nist.gov). Additionally, the GIAB v0.6 Tier 1 benchmark set was obtained from ftp://ftp-trace.ncbi.nlm.nih.gov/ReferenceSamples/giab/data/AshkenazimTrio/analysis/NIST_SVs_Integration_v0.6/. The whole-genome paired-end short reads and benchmark sets of HG00154, HG00733, and NA19240 were obtained from HGSVC2 (https://www.internationalgenome.org/data-portal/data-collection/hgsvc2). As such, the study does not involve direct participation of human subjects. Therefore, formal ethics approval and consent to participate are not applicable.*

6. We notice that your supplementary tables are included in the manuscript file. Please remove them and upload them with the file type 'Supporting Information'. Please ensure that each Supporting Information file has a legend listed in the manuscript after the references list.

**Response:**

We apologize for any confusion caused by the misplaced supplementary tables in the original manuscript. Supplementary tables have been removed from the manuscript file and uploaded as separate 'Supporting Information' files. The legend of supplementary files has been listed in the manuscript after the references list. Thank you so much for your kind reminder.

***Page 33:***

**Supporting information**

***S1 Fig.*** *Distance of neighbor SVs in combination strategies. The distribution of distances between start positions in neighbor SVs across different combination strategies. IQR: Interquartile Range.*

***S1 Table.*** *Types and sizes of all SVs detected by each individual algorithm in HG002.*

***S2 Table.*** *Types and sizes of all SVs detected by each individual algorithm in HG00514.*

***S3 Table.*** *Types and sizes of all SVs detected by each individual algorithm in HG00733.*

***S4 Table.*** *Types and sizes of all SVs detected by each individual algorithm in NA19240.*

***S5 Table.*** *Distribution of SVs in truth sets.*

***S6 Table.*** *Counts of neighboring SVs.*

***S7 Table.*** *Distribution of SVs detected by different combination strategies.*

***S1 File.*** *Example of the refinement process of “duplicated records.”*

***S2 File.*** *Combination of neighboring SVs from multiple callers.*

*Additional Editor Comments:*

Please address the reviewer's comments carefully. Please explain why the need for another benchmarking tests. I also noticed that you have not included any machine learning based algorithms.

**Response:**

We sincerely appreciate the detailed review and the valuable comments provided. Numerous short-read-based general-purpose SV detection algorithms have been published to date. However, existing evaluations often focus on comparisons of individual tools or new callers. For instance, a benchmarking study by Cameron DL and colleagues evaluated more than 40 SV callers published between 2009 and 2017 identified DELLY, LUMPY, Manta, and GRIDSS as popular tools based on their citation counts in the Web of Science database. Although SvABA, published in 2018, was not included, it has demonstrated robust performance in detecting both germline and somatic SVs in other assessments.

While numerous reviews have explored the theoretical foundations of these algorithms, comparisons of combination strategies and benchmarking efforts remain limited. Some studies evaluated the performance of combining the consensus of individual tools. Daniel L. Cameron et al. assessed 10 SV callers individually and in possible m-of-n ensembles using well-referenced cell lines. They found that while simple ensemble calling sometimes improves the performance of the best individual callers, it is highly sensitive to the specific callers chosen for the ensemble. Similarly, Kosugi et al. analyzed the performance of 69 SV detection algorithms and the effectiveness of combining pairs of these algorithms. They observed that when pairs of SV detection algorithms were analyzed, SVs commonly identified by multiple algorithms exhibited higher precision but lower recall than those identified by a single algorithm. Building on this, our study evaluated various combination strategies, including multiple agreement and union strategies, with existing free software—specifically the aforementioned five tools—compared to the commercial software DRAGEN. Our goal is to achieve comparable performance and highlight the utility of free tools. Our findings indicate that the union combination strategy significantly improves recall in detecting both deletions (DEL) and insertions (INS) compared to most of single tool, with recall and F1 scores comparable to those achieved with commercial tools, although the precision was not satisfactory.

In clinical practice, post-processing tasks like filtering target genes using a virtual panel, along with additional validation through the Integrative Genomics Viewer (IGV) or methods such as PCR, are commonly used. Therefore, given these considerations, we believe that achieving a high recall rate to minimize false negatives (FNs) significantly enhances the utility of these findings in clinical settings. Moreover, following the suggestion of Reviewer #1, in addition to the GIAB v6 benchmark set, we have incorporated three additional benchmark sets (HG00514, HG00733, NA19240) from Human Genome Structural Variation Consortium 2 (HGSVC2) to enhance our study's robustness. Thus, we believe the findings of our study are quite valuable.

We also sincerely appreciate your suggestion for implementation of machine learning (ML) based algorithms This is also an aspect that many existing comparative studies lack. Applying ML models requires considering various settings and hardware resources, which differs from sequencing-based methods. Building on the foundation of this study, we look forward to leveraging ML methods to enhance precision without compromising recall moving forward. As noted by Reviewer #1, Guiwu Zhuang et al. recently evaluated several short-read based algorithms for clinically relevant somatic SV detection, and developed a random-forest-based decision model to improve precision of the sequencing-based method.

Following your kind suggestion, we have thoroughly revised and added a discussion on the potential to further enhance precision through ML in the 'Discussion' section. Thank you again for your insightful comments. Below, we provide a point-by-point response to the specific issues raised by the reviewers.

*References:*

- *Cameron DL, Di Stefano L, Papenfuss AT. Comprehensive evaluation and characterization of short read general-purpose structural variant calling software. Nature Communications. 2019;10(1):3240. doi: 10.1038/s41467-019-11146-4.*
- *Kosugi S, Momozawa Y, Liu X, Terao C, Kubo M, Kamatani Y. Comprehensive evaluation of structural variation detection algorithms for whole genome sequencing. Genome Biology. 2019;20(1):117. doi: 10.1186/s13059-019-1720-5.*
- *Zhuang G, Zhang X, Du W, Xu L, Ma J, Luo H, et al. A benchmarking framework for the accurate and cost-effective detection of clinically-relevant structural variants for cancer target identification and diagnosis. Journal of Translational Medicine. 2024;22(1):65. doi: 10.1186/s12967-024-04865-w.*

***Page 24-27 :***

**Discussion**

*SVs* can have a substantial phenotypic impact, disrupting gene function, regulation, or modifying gene dosage. Their significance in medicine and molecular biology has been underscored by recent studies [28]. The role of *SVs* in the pathogenicity of genetic diseases is increasingly recognized, and there is a possibility that screening for *SVs* may become routine in clinical settings in the future. Despite the evidence underscoring the importance of *SVs*, they have garnered less attention compared to *SNVs* due to inherent challenges in identification. While theoretically, each type of *SV* produces a distinct pattern in read mapping, offering crucial details for recognizing the underlying alteration, the reality is more intricate. First, sequencing and mapping errors introduce uncertainty into the observed patterns. Second, the patterns generated by different types of *SVs* can exhibit striking similarities, complicating distinctions, such as between novel INSs and tandem DUPs in genomic alignments. Third, the overlap or nesting of multiple *SVs* introduces complex mapping patterns that are significantly more intricate [29].

*Sequencing-based methods theoretically possess the capability to detect any type of SV. However, no single computational methodology can reliably and sensitively identify every size and type of SV, as each detection method exhibits unique strengths and limitations across various SV sizes and types. Numerous studies have highlighted the individual performance of various callers. For example, Varuni Sarwal et al. [30] evaluated the performance of SV detection tools on mouse and human WGS data, focusing on DELs detection using a comprehensive set confirmed by polymerase chain reaction (PCR) and the GIAB benchmark set. Evaluations of combination strategies, however, remain relatively scarce. Some studies evaluated the performance of combining the consensus of individual tools. Daniel L. Cameron et al. [22] assessed 10 SV callers individually and in possible m-of-n ensembles using well-referenced cell lines such as NA12878, HG002, CHM1, and CHM13. They found that while simple ensemble calling sometimes improves the performance of the best individual callers, it is highly sensitive to the specific callers chosen for the ensemble. Similarly, Kosugi et al. [6] analyzed the performance of 69 SV detection algorithms and the effectiveness of combining pairs of these algorithms. They observed that when pairs of SV detection algorithms were analyzed, SVs commonly identified by multiple algorithms exhibited higher precision but lower recall than those identified by a single algorithm. Building on this, our study comprehensively assessed the performance of six state-of-the-art SV detection algorithms. We evaluated these algorithms individually to detail their respective abilities and limitations using both the Genome in a Bottle (GIAB) benchmark set and the Human Genome Structural Variation Consortium 2 (HGSVC2) benchmark set. Additionally, we proposed combination strategies that involved multiple agreement strategies, which requires the consensus of more than two tools, as well as union strategies that integrates the outputs of all selected algorithms.*

According to our study, relying on a single algorithm for SV detection may pose the risk of limited SV size and type detection, along with low F1 score. While our multiple agreement strategy, which focuses on consensus among algorithms, tends to yield higher precision at the cost of lower recall, employing a union combination strategy covers a broader range of SV sizes and diverse types more effectively. Specifically, the union strategy enhances recall and F1 scores for DELs and INSs within the SV size range of 50 to 10k base pairs compared to both single algorithms and the multiple agreement approach, though it does not improve precision. *In clinical practice, post-processing tasks like filtering target genes using a virtual panel, along with additional validation through the Integrative Genomics Viewer (IGV) or methods such as PCR, are commonly used. Therefore, weighing these considerations, we believe that achieving a high recall rate to minimize false negatives (FN) enhance the utility of these findings in clinical settings. ~~Our findings indicate that the union combination strategy significantly improves recall in detecting both deletions (DEL) and insertions (INS), with recall and F1 scores comparable to those achieved with commercial tools.~~*

*Additionally, although the overall performance of most single callers in detecting INSs is unsatisfactory due to a poor recall rate, the union strategy is particularly effective due to its comprehensive coverage, which capitalizes on the strong performance of individual callers like Manta in INS detection (Fig. 6). This underscores the importance of choosing well-performing callers tailored to specific types and sizes of SVs for different clinical scenarios. Recently, Ramesh Rajaby and colleagues developed an INS caller, INSurVeyor [31], which improves the sensitivity of INS calling from short-read sequencing data, performing even better than Manta. Building on the foundation of our study, we believe that by integrating algorithms that perform well for specific SV types, such as INS detection, and using a union strategy, excellent performance can be achieved tailored to various detection goals.*

*Moreover, recently, Guiwu Zhuang et al. [32] established an expert-reviewed tumor-specific clinically relevant SVs call set, evaluating several algorithms for SV detection, and developed a random-forest-based decision model to improve the precision. Building on these research foundations, it is worth exploring the potential of integrating the union combination strategy with an machine learning (ML) based decision model in the future. For practical clinical applications, further investigation into the performance using real clinical sample sets is necessary.*

*Finally, when addressing SV calling, the role of long-read sequencing cannot be overlooked. Long-read sequencing offers significant advantages as it can span repetitive or other problematic regions. For applications that require identifying very long or complex SVs, performing a localized long-read de novo assembly is more effective than conventional short-read mapping approaches. However, despite the advancement of long-read technologies and the continuous cost reductions by both Oxford Nanopore and PacBio, which have made long-read sequencing increasingly widespread, the costs remain substantial. Additionally, algorithms based on long reads generally have longer runtimes compared to standard algorithms using tools like BWA [6]. A more comprehensive assessment of these technologies is still lacking. Considering time, costs, different clinical practical scenario and the vast amount of existing data, an urgent need remains for an effective short-read-based detection strategy.*

*Although our study only focused on the DELs and INSs— which are the majority but relatively simple SV types in the human genome— methods failing to detect DELs and INSs might also be less effective at identifying more complex SV types. Currently, our study evaluated the ability of various methods and combination strategies to detect DELs and INSs, providing a valuable and optimistic perspective on overall SV detection performance.*

Reviewers' comments:

Reviewer's Responses to Questions

Comments to the Author

1. Is the manuscript technically sound, and do the data support the conclusions?

The manuscript must describe a technically sound piece of scientific research with data that supports the conclusions. Experiments must have been conducted rigorously, with appropriate controls, replication, and sample sizes. The conclusions must be drawn appropriately based on the data presented.

Reviewer #1: No

Reviewer #2: Partly

**Response:**

We sincerely appreciate for the detailed review and valuable comment. Following with the reviewers’ constructive suggestions, we add three sample sets (HG00514, 00733, NA19240) obtained from HGSVC2 to further examine the performance of different individual SV callers and combination strategies. We have revised our manuscript to make sure the conclusions are directly supported by the data presented. Any speculative statements have been revised or removed. We also have rewritten the discussion section includes a more thorough explanation of our conclusions. All the changes are labeled with red color in the revised manuscript. We thank again for reviewers’ constructive comment!

2. Has the statistical analysis been performed appropriately and rigorously?

Reviewer #1: No

Reviewer #2: Yes

**Response:**

Thank you for your comment! Following the reviewers’ constructive suggestions, we have added three sample sets (HG00514, 00733, NA19240) obtained from HGSVC2 to further examine the performance of different individual SV callers and combination strategies. We hope this strengthens the evidence supporting our conclusions. We sincerely appreciate the detailed review and valuable comments.

3. Have the authors made all data underlying the findings in their manuscript fully available? The PLOS Data policy requires authors to make all data underlying the findings described in their manuscript fully available without restriction, with rare exception (please refer to the Data Availability Statement in the manuscript PDF file). The data should be provided as part of the manuscript or its supporting information, or deposited to a public repository. For example, in addition to summary statistics, the data points behind means, medians and variance measures should be available. If there are restrictions on publicly sharing data—e.g. participant privacy or use of data from a third party—those must be specified.

Reviewer #1: Yes

Reviewer #2: Yes

**Response:**

We appreciate for your comment!

4. Is the manuscript presented in an intelligible fashion and written in standard English?

PLOS ONE does not copyedit accepted manuscripts, so the language in submitted articles must be clear, correct, and unambiguous. Any typographical or grammatical errors should be corrected at revision, so please note any specific errors here.

Reviewer #1: Yes

Reviewer #2: Yes

**Response:**

We appreciate for your comment!

5. Review Comments to the Author

Please use the space provided to explain your answers to the questions above. You may also include additional comments for the author, including concerns about dual publication, research ethics, or publication ethics. (Please upload your review as an attachment if it exceeds 20,000 characters)

**Response:**

Thank you for reviewers’ valuable comment on our manuscript. We appreciate the detailed review and have taken steps to address the concerns raised. Below, we provide a point-by-point response to the specific issues identified by the reviewers.

6. PLOS authors have the option to publish the peer review history of their article (what does this mean?). If published, this will include your full peer review and any attached files. If you choose “no”, your identity will remain anonymous but your review may still be made public. Do you want your identity to be public for this peer review? For information about this choice, including consent withdrawal, please see our Privacy Policy.

Reviewer #1: No

Reviewer #2: Yes: Wan-Ping Lee

**Response:**

We sincerely appreciate for the detailed review and valuable comment. We are comfortable with the peer review history being published, respecting the preferences of both reviewers.

**Reviewer #1**

*Comments to the Author*

The manuscript describes a benchmark experiment to detect structural variants from short reads. It uses the HG002 benchmark dataset of the Genome in a bottle project as gold standard. Due to lack of information in the gold standard or lack of events in some of the evaluated tools, this benchmark only takes into account deletions between 50 and 10kbp. Although I understand that reviews for PloS One should not be emphasized in novelty, I think benchmarking of SVs with GIAB data is one extreme case. I do not see a point on publishing over and over the same benchmark experiment with the exact same gold standard. Just by a simple google search with the words “benchmark structural variants detection”, I obtained the following papers:

- Zook et al., 2020. https://doi.org/10.1038/s41587-020-0538-8

- Sarwal et al., 2022. https://doi.org/10.1093/bib/bbac492

- Fang et al., 2024. https://doi.org/10.1186/s12967-024-04865-w

- Cameron et al., 2019. https://doi.org/10.1038/s41467-019-11146-4

The two more recent papers are not even cited in the literature. The main issue of making all benchmarking experiments on only one dataset as gold standard is that the development of the different tools is likely to be biased towards that particular dataset, which is likely to produce overfitting. Moreover, it is at least strange that even having an almost perfect diploid assembly for this dataset, there are still no gold-standard calls for inversions and duplications. I think that the authors should come up with at least one benchmark experiment different than GIAB, to really assess the accuracy of the different tools in cases different than those probably used to tune these tools. One source to at least improve the genetic diversity of the samples is to perform benchmarking using the datasets generated by the HGSVC2 consortium (see https://doi.org/10.1038/s41467-018-08148-z and https://doi.org/10.1126/science.abf7117)

**Response:**

We sincerely thank you for your valuable comments and constructive suggestions. Following your kind recommendation, we have now included the two more recent papers in our manuscript.

Indeed, there have been numerous studies utilizing the GIAB dataset to explore SV tool performance. While GIAB is highly commended for its comprehensiveness, as highlighted in a recent review by Sina Majidian et al. that discusses benchmark datasets, including those for medically relevant and complex genomic regions, we agree with your comment that relying solely on one dataset for performance evaluation is restrictive. Following with your suggestion, to address this concern and enhance our study's robustness, we have added three sample sets (HG00514, HG00733, NA19240) obtained from the Human Genome Structural Variation Consortium 2 (HGSVC2). We hope this addition will help improve the genetic diversity of the samples and provide a more comprehensive assessment of the tools' accuracy.

*Reference:*

- *Majidian, S., Agustinho, D.P., Chin, CS. et al. Genomic variant benchmark: Genomic variant benchmark: if you cannot measure it, you cannot improve it. Genome Biol 24, 221 (2023).*

Following with your suggestion, we have made the thoroughly revision of 'Results' and 'Discussion' sections, and all the changes are labeled with red color in the revised manuscript. Additionally, we have updated the figures and supplementary information to include data from the new sample sets and reorganized them for clarity. We sincerely appreciate your valuable comments and insightful suggestions!

***Page 11-24:***

**Results**

Thoroughly revision

***Page 24-27:***

***Discussion***

Thoroughly revision

***Fig. 1***

***Fig 1. SV detection workflow.*** *SV detection workflow starting with benchmark set collection from GIAB and HGSVC2, followed by sequence alignment using BWA-MEM to produce BAM files. These BAM files are then processed by various SV callers (DRAGEN, Manta, DELLY, LUMPY, GRIDSS, SvABA) to generate VCF files, which are subsequently used for performance assessment, including single tool and combination strategies, based on recall, precision, and F1 score.*

*Fig. 2*

*Fig 2. The SVs detected by six SV callers. (A) SVs of all sizes detected by individual SV callers in samples HG002, HG00514, HG00733, and NA19240. (B) Length distribution of different variants for all samples detected by individual SV callers. The dashed line indicates the range from 50-10k base pairs. The maximum, minimum, and median based on the integrated values from all sample sets. DEL: deletion, INS: insertion, DUP: duplication, INV: inversion, and CTX: complex translocation.*

*Fig. 3*

*Fig 3. The distribution of SVs in truth sets and the performance of individual algorithms. (A) The distribution of SVs size and types in truth sets. The dashed line separates the regions above and below 10k base pairs. (B) The comparison of False negative (FN) and False positive (FP) numbers among individual algorithms. (C) The precision, recall, and F1 score of each individual algorithm in detecting “DELs” and “INSs” within the specific size range of 50-10k base pairs. DEL: deletion, INS: insertion.*

*Fig 4*

*Fig 4. Concordance of neighbor SVs detected by member callers in different combination strategies. (A*) *The agreement among three SV detection tools (Manta, DELLY, and GRIDSS). (B) The agreement among five SV detection tools (Manta, DELLY, GRIDSS, LUMPY, and SvABA).*

*Fig 5*

*Fig 5. Distribution and performance of combination strategies. (A) The distribution of SVs in different combination strategies. (B) The comparison of False negative (FN) and False positive (FP) numbers among individual algorithms. (C) The precision, recall, and F1 score of combination strategies in the detection of DELs and INSs within the specific size range of 50-10K base pairs. DEL: deletion, INS: insertions, DUP: duplication, INV: inversion.*

*Fig 6*

***Fig 6. Combination performance of each single caller and combination strategy in detecting DELs and INSs.*** *The maximum, minimum, and macro average of recall, precision, and F1 score based on the integrated values from all sample sets.*

*Major comments*

I have some additional comments, as follows.

1. Lines 43 – 45, and 466 - 469. It is not clear which is the evidence to support the statements made in these lines of the abstract and the conclusions. They seem to be based solely on the number of variants produced by each tool, before the assessment using the gold-standard. In particular it is impossible to draw conclusions about translocations without a gold standard to assess the accuracy of these events.

**Response:**

We sincerely thank you for your kind reminder. To avoid overstating our findings, we have removed the statements lacking sufficient basis and have rewritten the sections of 'Abstract' and 'Conclusions' in our manuscript accordingly. We appreciate your comment!

***Page 2:***

**Abstract**

*................In this study, we assessed six state-of-the-art SV detection algorithms, including the commercial software DRAGEN, utilizing the GIAB (Genome in a Bottle) v0.6 Tier 1 benchmark set and high-quality referenced cell lines from HGSVC2 (Human Genome Structural Variation Consortium, Phase 2). Our evaluation incorporated both individual and combination strategies, with systematic assessments of recall, precision, and F1 scores. ~~DELLY struggled with complex translocations, and LUMPY had difficulty with~~* *~~insertions. GRIDSS excelled in smaller SVs, while LUMPY performed better with larger SVs. The combination approach showed diversity in SV types and sizes compared to single algorithms~~*..................*Our results demonstrate that union combination approach enhanced detection capabilities across a broader spectrum of SV types and sizes, surpassing single algorithms in identifying deletions and insertions, delivered comparable recall and F1 scores to the commercial software DRAGEN.*

***Page 28:***

**Conclusions**

In this study, *~~with a focus on clinical applicability~~*, we evaluated the performance of five state-of-the-art *SV* detection algorithms and a commercial software, DRAGEN, using short-read whole-genome sequence data from the GIAB v0.6 Tier 1 benchmark set *and well referenced benchmark set from HGSVC2.* We tested each algorithm independently and in various combinations, employing both multiple agreement and union strategies. *~~Among the six algorithms, DELLY failed to detect complex translocations, while LUMPY struggled with insertions. The remaining algorithms demonstrated varying success in detecting different SV types. GRIDSS excelled in identifying smaller SVs, whereas LUMPY performed better with larger SVs.~~* *The union approach achieved a higher recall rate, whereas the multiple strategy exhibited superior precision. Our analysis revealed that each algorithm had its intrinsic strengths and weaknesses, leading to variations in the types and sizes of SVs detected. The union combination strategies effectively coordinated these differences, enhancing overall performance, achieving similar F1 scores as the commercial software DRAGEN.*

2. Lines 52-56: Taking into account that the decisions regarding variant detection and interpretation have been rather conservative, I really doubt that a method in which the false positives of all tools are retained would be the recommended method for diagnosis in clinical settings.

**Response:**

Thank you for your comment! In clinical practice, post-processing tasks like filtering target genes using a virtual panel, along with additional validation through the Integrative Genomics Viewer (IGV) or methods such as PCR, are commonly used. Therefore, weighing these considerations, we believe that achieving a high recall rate to minimize false negatives (FN) enhance the utility of these findings in clinical settings. Our findings indicated the union strategy enhanced recall and F1 scores for DELs and INSs within the SV size range of 50 to 10k base pairs compared to both single algorithms and the multiple agreement approach.

As a literature you mentioned, Guiwu Zhuang et al. recently evaluated several short-read based algorithms for clinically relevant somatic SV detection, and developed a random-forest-based decision model to improve precision of the sequencing-based method. With this research foundation, we also aim to further improve precision by training a decision model and validating the feasibility of combining the union strategy with the decision model using real clinical data for germline SV detection in the future.

Following your comment, we have revised and added a discussion on the potential to further enhance precision through ML in the 'Discussion' section. Thank you again for your insightful comments.

*Reference:*

- *Zhuang G, Zhang X, Du W, Xu L, Ma J, Luo H, et al. A benchmarking framework for the accurate and cost-effective detection of clinically-relevant structural variants for cancer target identification and diagnosis. Journal of Translational Medicine. 2024;22(1):65. doi: 10.1186/s12967-024-04865-w.*

***Page 25-26:***

**Discussion**

...............According to our study, relying on a single algorithm for SV detection may pose the risk of limited SV size and type detection, along with low F1 score. While our multiple agreement strategy, which focuses on consensus among algorithms, tends to yield higher precision at the cost of lower recall, employing a union combination strategy covers a broader range of SV sizes and diverse types more effectively. Specifically, the union strategy enhances recall and F1 scores for DELs and INSs within the SV size range of 50 to 10k base pairs compared to both single algorithms and the multiple agreement approach, though it does not improve precision. *In clinical practice, post-processing tasks like filtering target genes using a virtual panel, along with additional validation through the Integrative Genomics Viewer (IGV) or methods such as PCR, are commonly used. Therefore, weighing these considerations, we believe that achieving a high recall rate to minimize false negatives (FN) enhance the utility of these findings in clinical settings. ~~Our findings indicate that the union combination strategy significantly improves recall in detecting both deletions (DEL) and insertions (INS), with recall and F1 scores comparable to those achieved with commercial tools.~~*

*Additionally, although the overall performance of most single callers in detecting INSs is unsatisfactory due to a poor recall rate, the union strategy is particularly effective due to its comprehensive coverage, which capitalizes on the strong performance of individual callers like Manta in INS detection (Fig. 6). This underscores the importance of choosing well-performing callers tailored to specific types and sizes of SVs for different clinical scenarios. Recently, Ramesh Rajaby and colleagues developed an INS caller, INSurVeyor [31], which improves the sensitivity of INS calling from short-read sequencing data, performing even better than Manta. Building on the foundation of our study, we believe that by integrating algorithms that perform well for specific SV types, such as INS detection, and using a union strategy, excellent performance can be achieved tailored to various detection goals.*

*Moreover, recently, Guiwu Zhuang et al. [32] established an expert-reviewed tumor-specific clinically relevant SVs call set, evaluating several algorithms for SV detection, and developed a random-forest-based decision model to improve the precision. Building on these research foundations, it is worth exploring the potential of integrating the union combination strategy with an machine learning (ML) based decision model in the future. For practical clinical applications, further investigation into the performance using real clinical sample sets is necessary.*................

3. Lines 94 – 96. This statement is less true each day. Both PacBio and Nanopore have made important cost reductions and improvements in quality, reducing the cost difference with Illumina. Taking into account that SV calling with long reads has a clearly superior accuracy, compared to SV calling with short reads, I would at least discuss if it is really worth to keep considering Illumina for SV detection in clinical settings.

**Response:**

Thank you for your comment! We agree that the role of long-read sequencing in SVs detection cannot be overlooked. Long-read sequencing offers significant advantages as it can span repetitive or other problematic regions. For applications that require identifying very long or complex SVs, performing a localized long-read de novo assembly is more effective than conventional short-read mapping approaches.

Despite advancements and cost reductions that have made long-read sequencing more accessible, significant challenges remain. These include not only higher costs, longer runtimes, scalability issues, and stringent sample quantity and quality requirements, but also important considerations regarding the utility of existing short-read data. For example, many biobanks host extensive archives of short-read data, with a persisting high demand for their reanalysis. The UK Biobank has sequenced the whole genomes of 150,119 participants using Illumina NovaSeq sequencing. Although the All of Us (AoU) project has re-sequenced some samples using long-read techniques, it has already released over 100,000 datasets from Illumina whole genome sequencing. Meanwhile, short-read whole-genome sequencing (WGS) has achieved high accuracy in detecting single nucleotide variants (SNVs) and remains widely utilized. The practical convenience of employing the same sequencing approach for both SNVs and structural variants (SVs) presents significant benefits.

Furthermore, the global market for short-read sequencing, valued at USD 5,341.3 million in 2022, is expected to grow at a compound annual growth rate of 18.46% from 2023 to 2030, driven by increasing demand for personalized medicine and companion diagnostics. Short-read technologies, capable of generating vast data volumes in a single run, are not only cost-effective but also user-friendly. These factors are anticipated to further drive the industry’s growth.

Therefore, while the advantages of long-read sequencing are evident, the need for an effective short-read-based detection strategy remains urgent, considering the time, costs, existing data, and varied clinical scenarios. A more comprehensive assessment of these technologies is still valuable to balance their application in different contexts, reinforcing the utility of short-read WGS for detecting both SNVs and SVs. As you suggest, evaluating the applications of both long and short-read sequencing continues to be a focal point of widespread attention and discussion, this topic has been discussed by researchers like Amarasinghe SL et al. and Mahmoud M as well.

*References:*

- *Halldorsson, B.V., Eggertsson, H.P., Moore, K.H.S. et al. The sequences of 150,119 genomes in the UK Biobank. Nature 607, 732–740 (2022). https://doi.org/10.1038/s41586-022-04965-x*
- *Mahmoud, M., Huang, Y., Garimella, K. et al. Utility of long-read sequencing for All of Us. Nat Commun 15, 837 (2024). https://doi.org/10.1038/s41467-024-44804-3*
- *Amarasinghe SL, Su S, Dong X, Zappia L, Ritchie ME, Gouil Q. Opportunities and challenges in long-read sequencing data analysis. Genome Biology. 2020;21(1):30. doi: 10.1186/s13059-020-1935-5.*
- *Mahmoud M, Gobet N, Cruz-Dávalos DI, Mounier N, Dessimoz C, Sedlazeck FJ. Structural variant calling: the long and the short of it. Genome Biology. 2019;20(1):246. doi: 10.1186/s13059-019-1828-7.*
- *Grand View Research. Market analysis report: Short-read Sequencing Market Size, Share & Trends Analysis Report By Product (Instruments, Consumables), By Workflow, By Application, By End-Use, By Region, And Segment Forecasts, 2023 – 2030. Retrieved from https://www.grandviewresearch.com/industry-analysis/short-read-sequencing-market.*

Following your comment, we have included this section in both the 'Introduction' and 'Discussion' sections of our manuscript. Thank you again for your valuable insights!

***Page 4:***

**Introduction**

................ *Recently, long-read technologies such as Oxford Nanopore Technologies (ONT) and PacBio HiFi sequencing have made significant advancements and become more accessible, offering higher accuracy to overcome the limitations of short-read sequencing [11]. However, despite these advancements and cost reductions making long-read sequencing more feasible, substantial challenges remain. These include not only higher costs, longer runtimes, scalability issues, and stringent requirements for sample quantity and quality[12, 13], but also important considerations for the vast amounts of existing short-read data. For instance, many biobanks contain extensive archives of short-read data, which continue to have a high demand for reanalysis. The UK Biobank, for example, has sequenced the whole genomes of 150,119 participants using Illumina NovaSeq sequencing [14]. Similarly, while the All of Us (AoU) project has re-sequenced some samples with long-read techniques, it has already released over 100,000 datasets from Illumina whole genome sequencing (WGS) [15] , highlighting the ongoing reliance on short-read approaches.*.................

***Page 26-27:***

**Discussion**

................ *Finally, when addressing SV calling, the role of long-read sequencing cannot be overlooked. Long-read sequencing offers significant advantages as it can span repetitive or other problematic regions. For applications that require identifying very long or complex SVs, performing a localized long-read de novo assembly is more effective than conventional short-read mapping approaches. However, despite the advancement of long-read technologies and the continuous cost reductions by both Oxford Nanopore and PacBio, which have made long-read sequencing increasingly widespread, the costs remain substantial. Additionally, algorithms based on long reads generally have longer runtimes compared to standard algorithms using tools like BWA [6]. A more comprehensive assessment of these technologies is still lacking. Considering time, costs, different clinical practical scenario and the vast amount of existing data, an urgent need remains for an effective short-read-based detection strategy.*

*Although our study only focused on the DELs and INSs— which are the majority but relatively simple SV types in the human genome— methods failing to detect DELs and INSs might also be less effective at identifying more complex SV types. Currently, our study evaluated the ability of various methods and combination strategies to detect DELs and INSs, providing a valuable and optimistic perspective on overall SV detection performance.*

4. Lines 99 – 104. Although the issue with annotation of SVs is true, the paper does not address this issue. I recommend removing these lines, and instead writing a reduced version of what is currently on the discussion, providing a brief description of the algorithms implemented in the SV calling tools.

**Response:**

We sincerely appreciate for the recommendation! Following with your constructive suggestions, we have removed these lines from 'Discussion', and revised this section in the 'Introduction'. Both 'Introduction' and 'Discussions' have been thoroughly revised. Thank you again for your constructive suggestion!

***Page 3-5:***

**Introduction**

*~~In addition, interpreting the potential functional consequences of structural variants SVs poses a significant challenge. Annotating SVs is often more complex due to the necessity of considering their length and establishing a reliable assessment of allele frequency for the underlying sequence. Furthermore, SV reporting can vary among different methods, even when using the standard Variant Call Format (VCF). These complexities can impede routine SV screening, limiting accurate identification and characterization for clinical diagnoses.~~*

And with thoroughly revision

***Page 24-27:***

**Discussion**

Thoroughly revision

5. Lines 157 – 168: The procedures to merge results of the different tools need more clarity. What happen if for two events separated 1Kbp there is one intermediate event exactly in the middle? It is not clear which percentage of (reciprocal) intersection is required to call two events the same. It is also not clear how the 500bp threshold was chosen. The merging called intersection is not really an intersection of the methods. It is more like a two out of n kind of union. It should not be called intersection in part because it does not make sense that the intersection of five methods resulted in more events than the intersection between three methods.

**Response:**

Thank you for your comment. To better illustrate our methodology, we have provided an S2 File that outlines our merge criteria, and revise the 'Method' section. In our study, we grouped SVs whose start positions are within +/- 500 bases of each other with same SV type. Based on the original study of the Genome in a Bottle (GIAB) benchmark set, the process to form the SV benchmark includes the criterion: “Filter Complex: If two or more supported variants ≥50 bp were within 1000 bp of each other, they were excluded because they are potentially complex or inaccurate.” This implies that, within a range of 1000 bp, usually only one SV is detected unless it is complex or inaccurate. Therefore, we referenced this threshold of 1000 bp and set up a ±500 base pairs threshold for our analysis.

In response to the concern, we also analyzed the distances between start positions of neighboring SVs, across different combination strategies, revealed that the vast majority of grouped SVs are actually within 20 bp of each other. This indicates that the distances between most neighboring SVs are very close. Additionally, we conducted a statistical analysis on the average count of neighboring SVs in each merging groups. For instance, in the group using five callers, there should be at most five neighboring SVs per group. Identifying more than five suggests the presence of additional, smaller individual SVs. Our data shows that, in the group using three SV callers, the majority had fewer than three member variants, and in the group using five SV callers, the majority had fewer than five member variants, as detailed in S6 Table. These findings indicate that our merging strategy of +/- 500 base pairs rarely grouped distinct SVs erroneously.

Moreover, considering that most short reads range from 300 to 500 bp in length, we believe that SVs within this range can be sequenced and accurately identified. In practice, using other tools such as the IGV or methods like PCR is commonly used. Therefore, weighing these considerations, we believe that achieving a high recall rate to minimize false negatives (FN) is more crucial for the benefits it brings to clinical practice.

We sincerely appreciate your comment. Moving forward, we will ensure to consider both distance and overlap criteria when setting merge thresholds in future studies, to further refine our approach and enhance the reliability of our results.

Besides, in response to your suggestion, we have renamed the "intersection" strategy to the "multiple-agreement" strategy. The multiple-agreement strategy defines a 'true-called variant' if the members of a neighboring SV are detected by at least two or more algorithms. Thanks again for your important insight! Following with you commend, we have revised these sections in 'Method' and 'Results' sections.

***Page 8:***

**Methods**

**Combination strategies of multiple algorithms**

Individual SVs were annotated with the name of their respective SV detection algorithms in the 'INFOa' field (e.g., caller=DELLY). For grouping, one or more variants from different SV callers were considered a neighbor group if they existed within the range of +/- 500 *bp of 'POS' (start position)* and shared the same 'SVTYPE.' *When referring to candidate SVs within a neighbor group, we retained the variant with the smallest start position as the representative.*

Merging variants was conducted in the following ways: a combination of three algorithms, DELLY, Manta, and GRIDSS, formed the 3-SV (III) combination group, while a combination of five algorithms, Manta, DELLY, GRIDSS, LUMPY, and SvABA, formed the 5-SV (V) combination group. *The “multiple agreement” strategy* defined a 'true-called variant' if the members of a neighbor SV were detected by at least two or more algorithms. Conversely, the union strategy defined a “true-called variant” if at least one member of a neighbor SV was detected by at least one algorithm, as *demonstrated in S2 File.*

***Page 17:***

**Results**

**Systematic identification of the performances of combination strategies**

................ For our three-algorithm combination strategies, we selected Manta, DELLY, and GRIDSS, showcasing superior performance among all tools. Meanwhile, in the case of five-algorithm combination strategies, we considered all tools except the commercial DRAGEN. *Each individual SV variant was annotated by the name of its SV detection caller in the 'INFO' field. Based on the original study of the Genome in a Bottle (GIAB) benchmark set, the process to form the SV benchmark includes the criterion: “Filter Complex: If two or more supported variants ≥50 bp were within 1000 bp of each other, they were excluded because they are potentially complex or inaccurate.” Therefore, we referenced this threshold of 1000 bp and set up a ±500 base pairs threshold for our analysis.* Variants from different SV callers were grouped as a neighbor SV if they overlapped within the range of +/- 500 *base pairs* of 'POS' *(start position)* and shared the same 'SVTYPE.' The “*multiple agreement*” strategy categorizes variants as 'true-called' only if all members of the neighboring SV are detected by at least two algorithms. In contrast, the union strategy deems a variant as 'true-called' if at least one member of the neighboring SV is detected by at least one algorithm. *We evaluated the distance distribution of variant positions in neighboring SVs across different combination strategies (S1 Fig). The counts of neighboring SVs* *and* *the number of contributing member callers across different combination strategies are also presented in S6 Table. All variants in a neighbor SV were within the range of 0 and 500 base pairs, as per our setting in the combination of structural variants. The median distance of neighboring SVs across sample sets in III- multiple agreement, V- multiple agreement, III-union, and V-union were 4.0, 7.0-10.0, 0, and 0 base pairs respectively. The Interquartile Range (IQR) of neighbor SVs across sample sets in III-multiple agreement, V-multiple agreement, III-union, and V-union were 6.0-7.0, 15.0-16.0, 2.0, and 6.0-9.0 base pairs, respectively (S1_Fig), indicating that the majority of neighboring SV distances were very closed. The majority count of neighboring SVs within the 3 SV-caller group was fewer than 3 member variants, and the majority count of neighboring SVs within the 5 SV-caller group was fewer than 5 member variants (S6 Table). These indicate that very few distinct SVs were grouped due to the +/- 500 base pairs merge threshold.*

*S2 File Combination of neighboring SV from multiple callers.*

*S1 Fig*

***S1 Fig. Distance of neighbor SVs in combination strategies.*** *The distribution of distances between start positions in neighbor SVs across different combination strategies. IQR: Interquartile Range.*

*S6 Table*

6. Lines 180 – 184. The procedure to call a variant true positive has the same issues as the procedure to merge calls. The 500 bp limit looks arbitrary, especially if no reciprocal overlap is required. Moreover, it looks too relaxed for relatively small events (lets’ say less than 150 bp), which are a large percentage of the total events.

**Response:**

Thank you for your comment! Addressing the same concerns related to the call merging process. In our study, each individual SV variant was annotated by the name of its SV detection caller in the 'INFO' field. Based on the original study of the Genome in a Bottle (GIAB) benchmark set, the process to form the SV benchmark includes the criterion: “Filter Complex: If two or more supported variants ≥50 bp were within 1000 bp of each other, they were excluded because they are potentially complex or inaccurate.” Therefore, we referenced this threshold of 1000 bp and set up a ±500 base pairs threshold for our analysis. Although we did not initially consider both distance and overlap criteria, each caller or combination strategy was based on the same threshold to compare the performance.

Moreover, considering that most short reads range from 300 to 500 bp in length, we believe that SVs within this range can be sequenced and accurately identified. In practice, using other tools such as the IGV or methods like PCR is commonly used. Therefore, weighing these considerations, we believe that achieving a high recall rate to minimize false negatives (FN) is more crucial for the benefits it brings to clinical practice.

Again, we sincerely appreciate your comment. Moving forward, we will ensure to consider both distance and overlap criteria when setting merge thresholds in future studies, to further refine our approach and enhance the reliability of our results.

7. Lines 271 – 274. It does not make sense to restrict the benchmark only to deletions just because two methods do not call insertions. Benchmarking of insertions should be included for the methods that predict insertions. Because comparing true and predicted insertions is more challenging, compared to deletions, it is important to define and present clearly specific rules to perform both merging and intersection of insertions.

**Response:**

Thank you for your comment! Actually, we have also conducted a performance analysis of insertions with HG002. However, the performance of most single algorithms and the combination strategies was disappointing. Following your suggestion, we have added three additional benchmark sets from HGSVC2. The results of the insertions detection are now also included in the 'Results' section, and we have discussed these findings further in the 'Discussion' section.

***Page 11-24:***

**Results**

Thoroughly Revision.

***Page 25-26:***

**Discussion**

............. According to our study, relying on a single algorithm for SV detection may pose the risk of limited SV size and type detection, along with low F1 score. While our multiple agreement strategy, which focuses on consensus among algorithms, tends to yield higher precision at the cost of lower recall, employing a union combination strategy covers a broader range of SV sizes and diverse types more effectively. Specifically, the union strategy enhances recall and F1 scores for DELs and INSs within the SV size range of 50 to 10k base pairs compared to both single algorithms and the multiple agreement approach, though it does not improve precision. *In clinical practice, post-processing tasks like filtering target genes using a virtual panel, along with additional validation through the Integrative Genomics Viewer (IGV) or methods such as PCR, are commonly used. Therefore, weighing these considerations, we believe that achieving a high recall rate to minimize false negatives (FN) enhance the utility of these findings in clinical settings. Our findings indicate that the union combination strategy significantly improves recall in detecting both deletions (DEL) and insertions (INS), with recall and F1 scores comparable to those achieved with commercial tools. Currently, our study evaluated the ability of various methods to detect DELs and INSs, providing a valuable and optimistic perspective on overall SV detection performance. Although our study only focused on the DELs and INSs— which are the majority but relatively simple SV types in the human genome— methods failing to detect DELs and INSs might also be less effective at identifying more complex SV types.*

*Additionally, although the overall performance of most single callers in detecting INSs is unsatisfactory due to a poor recall rate, the union strategy is particularly effective due to its comprehensive coverage, which capitalizes on the strong performance of individual callers like Manta in INS detection (Fig. 6). This underscores the importance of choosing well-performing callers tailored to specific types and sizes of SVs for different clinical scenarios. Recently, Ramesh Rajaby and colleagues developed an INS caller, INSurVeyor [31], which improves the sensitivity of INS calling from short-read sequencing data, performing even better than Manta. Building on the foundation of our study, we believe that by integrating algorithms that perform well for specific SV types, such as INS detection, and using a union strategy, excellent performance can be achieved tailored to various detection goals.*

*Fig. 2*

*Fig 2. The SVs detected by six SV callers. (A) SVs of all sizes detected by individual SV callers in samples HG002, HG00514, HG00733, and NA19240. (B) Length distribution of different variants for all samples detected by individual SV callers. The dashed line indicates the range from 50-10k base pairs. The maximum, minimum, and median based on the integrated values from all sample sets. DEL: deletion, INS: insertion, DUP: duplication, INV: inversion, and CTX: complex translocation.*

*Fig. 3*

*Fig 3. The distribution of SVs in truth sets and the performance of individual algorithms. (A) The distribution of SVs size and types in truth sets. The dashed line separates the regions above and below 10k base pairs. (B) The comparison of False negative (FN) and False positive (FP) numbers among individual algorithms. (C) The precision, recall, and F1 score of each individual algorithm in detecting “DELs” and “INSs” within the specific size range of 50-10k base pairs. DEL: deletion, INS: insertion.*

*Fig 4*

*Fig 4. Concordance of neighbor SVs detected by member callers in different combination strategies. (A*) *The agreement among three SV detection tools (Manta, DELLY, and GRIDSS). (B) The agreement among five SV detection tools (Manta, DELLY, GRIDSS, LUMPY, and SvABA).*

*Fig 5*

*Fig 5. Distribution and performance of combination strategies. (A) The distribution of SVs in different combination strategies. (B) The comparison of False negative (FN) and False positive (FP) numbers among individual algorithms. (C) The precision, recall, and F1 score of combination strategies in the detection of DELs and INSs within the specific size range of 50-10K base pairs. DEL: deletion, INS: insertions, DUP: duplication, INV: inversion.*

*Fig 6*

***Fig 6. Combination performance of each single caller and combination strategy in detecting DELs and INSs.*** *The maximum, minimum, and macro average of recall, precision, and F1 score based on the integrated values from all sample sets.*

8. Lines 313- 316. I would not say that the unsatisfactory results are compared to Dragen. I would say that the results are unsatisfactory overall.

**Response:**

Thank you for your comment! In our study, we aim to explore free software options to enhance performance. Our findings indicate that the union combine strategy has better recall compared to single tools or the "multiple-agreement approach." Based on this discovery, we hope to further improve precision through additional algorithms, including machine learning algorithms in the near future. Thank you again for your comment!

9. Line 337. It is not surprising that the maximum is 500 if that corresponds to the threshold set by the method. Again, it looks too relaxed for relatively small variants.

**Response:**

Thank you for your comment! Indeed, all variants in a neighboring SV group were within the range of 0 to 500 base pairs, as per our settings in the combination of structural variants The median distance of neighbor SVs across sample sets in III-multiple agreement, V-multiple agreement, III-union, and V-union were 4.0, 7.0-10.0, 0, and 0 base pairs respectively. The Interquartile Range (IQR) of neighboring SVs across sample sets in III- multiple agreement, V- multiple agreement, III-union, and V-union were 6.0-7.0, 15.0-16.0, 2.0, and 6.0-9.0 base pairs, respectively (S1 Fig). This indicates that the majority of neighboring SV distances were very close.

We greatly appreciate your suggestion. Moving forward, we will ensure to consider incorporating both distance and overlap criteria when setting the merge criteria in our future analyses. In response to your comment, we have revised the manuscript to clarify this section in the 'Results.'

***Page 18-19:***

**Results**

................ *We evaluated the distance distribution of variant positions in neighboring SVs across different combination strategies (S1 Fig). The counts of neighboring SVs and the number of contributing member callers across different combination strategies are also presented in S6 Table. All variants in a neighboring SV group were within the range of 0 and 500 base pairs, as per our setting in the combination of structural variants. The median distance of neighboring SVs across sample sets in III-multiple agreement, V-multiple agreement, III-union, and V-union were 4.0, 7.0-10.0, 0, and 0 base pairs respectively. The Interquartile Range (IQR) of neighboring SVs across sample sets in III-multiple agreement, V-multiple agreement, III-union, and V-union were 6.0-7.0, 15.0-16.0, 2.0, and 6.0-9.0 base pairs, respectively (S1 Fig), indicating that the majority of neighboring SV distances were very closed. The majority count of neighboring SVs within the 3 SV-caller group was fewer than 3 member variants, and the majority count of neighboring SVs within the 5 SV-caller group was fewer than 5 member variants (S6 Table). These indicate that very few distinct SVs were grouped due to the +/- 500 base pairs merge threshold.*................

10. Lines 461 – 463. I do not see evidence of anything different or special in this work to say that it was focused on clinical applicability. There is no information on a disease or condition that could be related to the SVs detected on HG002. The authors should clarify what is their meaning of “clinical applicability”.

**Response:**

Thank you for your comment! We understand your concern, and to address this, we have removed and revised the lines in the 'Conclusions' to avoid overstating our findings. Additionally, we have expanded the discussion on clinical applicability in the 'Discussion' section.

***Page 28:***

**Conclusions**

In this study, *~~with a focus on clinical applicability~~,* we evaluated the performance of five state-of-the-art SV detection algorithms and a commercial software, DRAGEN, using short-read whole-genome sequence data from the GIAB v0.6 Tier 1 benchmark set and well referenced benchmark set from HGSVC2..................

***Page 24-25:***

**Discussion**

*In clinical practice, post-processing tasks like filtering target genes using a virtual panel, along with additional validation through the Integrative Genomics Viewer (IGV) or methods such as PCR, are commonly used. Therefore, weighing these considerations, we believe that achieving a high recall rate to minimize false negatives (FN) enhance the utility of these findings in clinical settings.* ~~Our findings indicate that the union combination strategy significantly improves recall in detecting both deletions (DEL) and insertions (INS), with recall and F1 scores comparable to those achieved with commercial tools.~~ Currently, our study evaluated the ability of various methods to detect DELs and INSs, providing a valuable and optimistic perspective on overall SV detection performance. Although our study only focused on the DELs and INSs— which are the majority but relatively simple SV types in the human genome— methods failing to detect DELs and INSs might also be less effective at identifying more complex SV types.

*Additionally, although the overall performance of most single callers in detecting INSs is unsatisfactory due to a poor recall rate, the union strategy is particularly effective due to its comprehensive coverage, which capitalizes on the strong performance of individual callers like Manta in INS detection (Fig. 6). This underscores the importance of choosing well-performing callers tailored to specific types and sizes of SVs for different clinical scenarios. Recently, Ramesh Rajaby and colleagues developed an INS caller, INSurVeyor [31], which improves the sensitivity of INS calling from short-read sequencing data, performing even better than Manta. Building on the foundation of our study, we believe that by integrating algorithms that perform well for specific SV types, such as INS detection, and using a union strategy, excellent performance can be achieved tailored to various detection goals.*

*Moreover, recently, Guiwu Zhuang et al. [32] established an expert-reviewed tumor-specific clinically relevant SVs call set, evaluating several algorithms for SV detection, and developed a random-forest-based decision model to improve the precision. Building on these research foundations, it is worth exploring the potential of integrating the union combination strategy with an machine learning (ML) based decision model in the future. For practical clinical applications, further investigation into the performance using real clinical sample sets is necessary..................*

11. Finally, most of the current discussion could be written without the results of this study. Most of the text could be moved and summarized in the introduction. The text includes many sentences with qualitative assessments of each tool that are not related to the results, and in some cases are even contradictory. The description of the tools could be summarized in a couple of paragraphs of the introduction, avoiding a-priori qualitative assessments about the performance of the algorithms. Once the other comments are solved, especially finding additional gold standards and evaluating more types of variants, an actual discussion could be written based on the results of the new experiments.

**Response:**

Thank you for your comment! Following your suggestion, we have thoroughly revised and reorganized the 'Introduction' and 'Discussions' sections. In accordance with your advice, we have also added three sample sets from the Human Genome Structural Variation Consortium 2 (HGSVC2) and incorporated the analysis of insertion (INS) detection into the 'Discussions' section. Thank you again for your constructive suggestions.

***Page 4-5:***

**Introduction**

................With the advent of next-generation sequencing, the detection of SNVs and small indels (<50 base pairs) has become highly accurate, with an error rate of less than 0.1%. Despite substantial advancements in numerous structural variant (SV) detection methods, their effectiveness in short-read sequences remains limited. Sequencing-based methods employ several approaches to derive information about SVs from short-read sequencing data: read-pair (RP), read-depth (RD), split-read (SR), and assembly (AS) approaches [5-7]. *RP uses the discordant alignment features of paired-end reads that encompass or overlap an SV. RD utilizes the depth features of paired-end reads that overlap an SV, indicating changes in read depth due to the SV. SR uses split (soft-clipped) alignment features of single-end or paired-end reads that span a breakpoint (BP) of an SV. AS detects SVs by aligning contigs, assembled from the entire or unmapped sequencing reads, to the reference sequence.*

*Despite significant growth and improvement in numerous structural variant (SV) detection methods over the years, their performance in short-read sequences remains constrained. Sequencing-based approaches exhibit a high rate of SV miscalling, irrespective of the strategy employed. To address the limitations of short-read sequencing, recent efforts have utilized long reads generated through single-molecule sequencing technology for SV detection in human samples, employing assembly (AS) and/or split-read (SR) approaches [8-10].*

*Recently, long-read technologies such as Oxford Nanopore Technologies (ONT) and PacBio HiFi sequencing have made significant advancements and become more accessible, offering higher accuracy to overcome the limitations of short-read sequencing [11]. However, despite these advancements and cost reductions making long-read sequencing more feasible, substantial challenges remain. These include not only higher costs, longer runtimes, scalability issues, and stringent requirements for sample quantity and quality[12, 13], but also important considerations for the vast amounts of existing short-read data. For instance, many biobanks contain extensive archives of short-read data, which continue to have a high demand for reanalysis. The UK Biobank, for example, has sequenced the whole genomes of 150,119 participants using Illumina NovaSeq sequencing [14]. Similarly, while the All of Us (AoU) project has re-sequenced some samples with long-read techniques, it has already released over 100,000 datasets from Illumina WGS [15] , highlighting the ongoing reliance on short-read approaches..................*

***Page 24-27 :***

**Discussion**

*SVs* can have a substantial phenotypic impact, disrupting gene function, regulation, or modifying gene dosage. Their significance in medicine and molecular biology has been underscored by recent studies [28]. The role of *SVs* in the pathogenicity of genetic diseases is increasingly recognized, and there is a possibility that screening for *SVs* may become routine in clinical settings in the future. Despite the evidence underscoring the importance of *SVs*, they have garnered less attention compared to *SNVs* due to inherent challenges in identification. While theoretically, each type of *SV* produces a distinct pattern in read mapping, offering crucial details for recognizing the underlying alteration, the reality is more intricate. First, sequencing and mapping errors introduce uncertainty into the observed patterns. Second, the patterns generated by different types of *SVs* can exhibit striking similarities, complicating distinctions, such as between novel INSs and tandem DUPs in genomic alignments. Third, the overlap or nesting of multiple *SVs* introduces complex mapping patterns that are significantly more intricate [29].

*Sequencing-based methods theoretically possess the capability to detect any type of SV. However, no single computational methodology can reliably and sensitively identify every size and type of SV, as each detection method exhibits unique strengths and limitations across various SV sizes and types. Numerous studies have highlighted the individual performance of various callers. For example, Varuni Sarwal et al. [30] evaluated the performance of SV detection tools on mouse and human WGS data, focusing on DELs detection using a comprehensive set confirmed by polymerase chain reaction (PCR) and the GIAB benchmark set. Evaluations of combination strategies, however, remain relatively scarce. Some studies evaluated the performance of combining the consensus of individual tools. Daniel L. Cameron et al. [22] assessed 10 SV callers individually and in possible m-of-n ensembles using well-referenced cell lines such as NA12878, HG002, CHM1, and CHM13. They found that while simple ensemble calling sometimes improves the performance of the best individual callers, it is highly sensitive to the specific callers chosen for the ensemble. Similarly, Kosugi et al. [6] analyzed the performance of 69 SV detection algorithms and the effectiveness of combining pairs of these algorithms. They observed that when pairs of SV detection algorithms were analyzed, SVs commonly identified by multiple algorithms exhibited higher precision but lower recall than those identified by a single algorithm. Building on this, our study comprehensively assessed the performance of six state-of-the-art SV detection algorithms. We evaluated these algorithms individually to detail their respective abilities and limitations using both the Genome in a Bottle (GIAB) benchmark set and the Human Genome Structural Variation Consortium 2 (HGSVC2) benchmark set. Additionally, we proposed combination strategies that involved multiple agreement strategies, which requires the consensus of more than two tools, as well as union strategies that integrates the outputs of all selected algorithms.*

According to our study, relying on a single algorithm for SV detection may pose the risk of limited SV size and type detection, along with low F1 score. While our multiple agreement strategy, which focuses on consensus among algorithms, tends to yield higher precision at the cost of lower recall, employing a union combination strategy covers a broader range of SV sizes and diverse types more effectively. Specifically, the union strategy enhances recall and F1 scores for DELs and INSs within the SV size range of 50 to 10k base pairs compared to both single algorithms and the multiple agreement approach, though it does not improve precision. *In clinical practice, post-processing tasks like filtering target genes using a virtual panel, along with additional validation through the Integrative Genomics Viewer (IGV) or methods such as PCR, are commonly used. Therefore, weighing these considerations, we believe that achieving a high recall rate to minimize false negatives (FN) enhance the utility of these findings in clinical settings. ~~Our findings indicate that the union combination strategy significantly improves recall in detecting both deletions (DEL) and insertions (INS), with recall and F1 scores comparable to those achieved with commercial tools.~~ Currently, our study evaluated the ability of various methods to detect DELs and INSs, providing a valuable and optimistic perspective on overall SV detection performance. Although our study only focused on the DELs and INSs— which are the majority but relatively simple SV types in the human genome— methods failing to detect DELs and INSs might also be less effective at identifying more complex SV types.*

*Additionally, although the overall performance of most single callers in detecting INSs is unsatisfactory due to a poor recall rate, the union strategy is particularly effective due to its comprehensive coverage, which capitalizes on the strong performance of individual callers like Manta in INS detection (Fig. 6). This underscores the importance of choosing well-performing callers tailored to specific types and sizes of SVs for different clinical scenarios. Recently, Ramesh Rajaby and colleagues developed an INS caller, INSurVeyor [31], which improves the sensitivity of INS calling from short-read sequencing data, performing even better than Manta. Building on the foundation of our study, we believe that by integrating algorithms that perform well for specific SV types, such as INS detection, and using a union strategy, excellent performance can be achieved tailored to various detection goals.*

*Moreover, recently, Guiwu Zhuang et al. [32] established an expert-reviewed tumor-specific clinically relevant SVs call set, evaluating several algorithms for SV detection, and developed a random-forest-based decision model to improve the precision. Building on these research foundations, it is worth exploring the potential of integrating the union combination strategy with an machine learning (ML) based decision model in the future. For practical clinical applications, further investigation into the performance using real clinical sample sets is necessary.*

*Finally, when addressing SV calling, the role of long-read sequencing cannot be overlooked. Long-read sequencing offers significant advantages as it can span repetitive or other problematic regions. For applications that require identifying very long or complex SVs, performing a localized long-read de novo assembly is more effective than conventional short-read mapping approaches. However, despite the advancement of long-read technologies and the continuous cost reductions by both Oxford Nanopore and PacBio, which have made long-read sequencing increasingly widespread, the costs remain substantial. Additionally, algorithms based on long reads generally have longer runtimes compared to standard algorithms using tools like BWA [6]. A more comprehensive assessment of these technologies is still lacking. Considering time, costs, different clinical practical scenario and the vast amount of existing data, an urgent need remains for an effective short-read-based detection strategy.*

*Minor comments*

12. Figures and tables: The distribution between main and supplementary materials looks arbitrary. If supplementary tables are going to be embedded in the text, then they should not be called supplementary.

**Response:**

We apologize for any confusion caused by the misplaced supplementary tables in the original manuscript. Supplementary tables have been removed from the manuscript file and uploaded as separate 'Supporting Information' files. The legend of supplementary files has been listed in the manuscript after the references list. Thank you so much for your kind reminder.

***Page 33:***

**Supporting information**

***S1 Fig.*** *Distance of neighbor SVs in combination strategies. The distribution of distances between start positions in neighbor SVs across different combination strategies. IQR: Interquartile Range.*

***S1 Table.*** *Types and sizes of all SVs detected by each individual algorithm in HG002.*

***S2 Table.*** *Types and sizes of all SVs detected by each individual algorithm in HG00514.*

***S3 Table.*** *Types and sizes of all SVs detected by each individual algorithm in HG00733.*

***S4 Table.*** *Types and sizes of all SVs detected by each individual algorithm in NA19240.*

***S5 Table.*** *Distribution of SVs in truth sets.*

***S6 Table.*** *Counts of neighboring SVs.*

***S7 Table.*** *Distribution of SVs detected by different combination strategies.*

***S1 File.*** *Example of the refinement process of “duplicated records.”*

***S2 File.*** *Combination of neighboring SV from multiple callers.*

13. Lines 150 -151. The sentence itself does not say much. I do not understand what is stringent about the filtering procedure. The filters mentioned in the text look fairly standard.

**Response:**

Thank you for reminder. We have removed this sentence. Thanks again for your kind reminder.

***Page 7:***

**Methods**

**Calling and refinement of structural variations**

Refinement and filtering of the variants underwent a systematic four-step process. In Step 1, we employed the R package for *SV* annotation [27], to annotate the types and lengths of the identified SVs. Moving to Step 2, *SVs* with lengths less than 50 base pairs (bp) were excluded from further consideration. *In Step 3, the output files generated by some SV callers (GRIDSS, SvABA, LUMPY, and Manta), there can be two separate records representing the same SV. One record typically shows the start position of the variant, while the other shows the end position. We removed the record that shows the end position, ensuring that there is only one record per unique SV, as visually depicted in S1 Fig.* Finally, Step 4 involved the selection of variants with a 'PASS' status in the 'FILTER' field. *~~This stringent filtering process aimed to enhance the precision and reliability of the identified SVs, streamlining the dataset for subsequent analyses and interpretation.~~*

14. Figures 1 and 2 should be composed to understand why the subfigures should go together. The figures look disorganized in the submitted version.

**Response:**

Thank you for commend. We have re-organized Figures 1 and 2. The subfigures was provided as supplementary data. In accordance with your advice, we have also added three sample sets from the Human Genome Structural Variation Consortium 2 (HGSVC2) and incorporated the analysis of insertion (INS) detection into the 'Results' section. All the figures, tables, and supplementary information were also thoroughly amended and reorganized. Thank you again for your constructive suggestions.

***Fig. 1***

***Fig 1. SV detection workflow.*** *SV detection workflow starting with benchmark set collection from GIAB and HGSVC2, followed by sequence alignment using BWA-MEM to produce BAM files. These BAM files are then processed by various SV callers (DRAGEN, Manta, DELLY, LUMPY, GRIDSS, SvABA) to generate VCF files, which are subsequently used for performance assessment, including single tool and combination strategies, based on recall, precision, and F1 score.*

*Fig. 2*

*Fig 2. The SVs detected by six SV callers. (A) SVs of all sizes detected by individual SV callers in samples HG002, HG00514, HG00733, and NA19240. (B) Length distribution of different variants for all samples detected by individual SV callers. The dashed line indicates the range from 50-10k base pairs. The maximum, minimum, and median based on the integrated values from all sample sets. DEL: deletion, INS: insertion, DUP: duplication, INV: inversion, and CTX: complex translocation.*

*Fig. 3*

*Fig 3. The distribution of SVs in truth sets and the performance of individual algorithms. (A) The distribution of SVs size and types in truth sets. The dashed line separates the regions above and below 10k base pairs. (B) The comparison of False negative (FN) and False positive (FP) numbers among individual algorithms. (C) The precision, recall, and F1 score of each individual algorithm in detecting “DELs” and “INSs” within the specific size range of 50-10k base pairs. DEL: deletion, INS: insertion.*

*Fig 4*

*Fig 4. Concordance of neighbor SVs detected by member callers in different combination strategies. (A*) *The agreement among three SV detection tools (Manta, DELLY, and GRIDSS). (B) The agreement among five SV detection tools (Manta, DELLY, GRIDSS, LUMPY, and SvABA).*

*Fig 5*

*Fig 5. Distribution and performance of combination strategies. (A) The distribution of SVs in different combination strategies. (B) The comparison of False negative (FN) and False positive (FP) numbers among individual algorithms. (C) The precision, recall, and F1 score of combination strategies in the detection of DELs and INSs within the specific size range of 50-10K base pairs. DEL: deletion, INS: insertions, DUP: duplication, INV: inversion.*

*Fig 6*

***Fig 6. Combination performance of each single caller and combination strategy in detecting DELs and INSs.*** *The maximum, minimum, and macro average of recall, precision, and F1 score based on the integrated values from all sample sets.*

15. Font types and resolution should be improved for Figures 3A and 3B.

**Response:**

Thank you for your reminder for the resolution issue. We have redrawn all the figures and improved the font type resolution.

16. The supplementary figure 3 has essentially the same information as Figure 2A. Moreover, it does not make sense to include bars for inversions and duplications if the dataset does not include these events.

**Response:**

Thank you for your reminder. We have removed the redundant supplementary figure and reorganized, amended, and updated all the figures and supplementary materials accordingly. Thank you again for your constructive suggestions.

**Reviewer #2**

*Comments to the Author*

Duan et al., assessed six SV calling algorithms, including DRAGEN, DELLY, LUMPY, GRIDSS, SvABA, and Manta. The assessment was done on HG002/NA24385 sample from Genome in a Bottle (GIAB).

*Major comments*

1. Reference Genome: The assessment was done on alignments against GRCh37, which is less complete than GRCh38. Please explain why GRCh38 was not used.

**Response:**

Thank you for your comment! In our study, we utilized the GIAB truth set aligned against GRCh37 for our assessments, which was the version available at the time of our research. Furthermore, following another reviewer’s suggestion, we added three sample sets from HGSVC2 (HG00514, HG00733, and NA19240), aligning them with hg38 to remain consistent with the reference settings of the truth sets.

However, we acknowledge that a more recent version of the GIAB has released a draft benchmark aligned against GRCh38. Moving forward, we will consider transitioning to GRCh38 for our assessments in future studies. We have updated and added this information to the 'Results' and 'Methods' sections of our manuscript. Thank you again for your constructive comments!

***Page 7:***

**Methods**

**Calling and refinement of structural variations**

We obtained the whole-genome *paired-end short reads* of HG002 (NA24385) from the National Institute of Standards and Technology (https://www.nist.gov) and *HG00514, HG00733, and NA19240 were obtained from HGSVC2 (https://www.internationalgenome.org/data-portal/data-collection/hgsvc2*). These raw reads were aligned to the GRCh37 version (for HG002) or *GRCh38 version (for HG00514, HG00733, and NA19240)* of the human reference genome using BWA-MEM, *as the SV benchmark set is based on references.* Subsequently, the resulting BAM files were processed through various SV detection algorithms, utilizing their default parameters as recommended by the authors for calling SVs. Subsequently, the resulting BAM files were input into various SV detection algorithms with their default parameters based on the authors' recommendations for calling SVs.

***Page 12:***

**Results**

**Evaluation of SV detection methods based on the GIAB benchmark set and well referenced cell lines**

*Based on the reference settings consistent with the truth sets，*the raw reads underwent alignment to the GRCh37 *(for HG002) and hg38 (for HG00514, HG00733, and NA19240* ) version of the human reference genome using BWA-MEM followed by our analysis workflow *(Fig 1).*

2. SV Truth Set: GIAB should have an officially released SV list. Why was this not used as the Truth set instead of defining your own?

**Response:**

Thank you for your comment! We indeed used the GIAB released SV list and performed only standard filtering. Variants meeting specific criteria were selected as 'TRUTH' variants 1. Variants with lengths ranging from 50 to 10k bases. 2. Variants of the SV type 'DEL' and 'INS'. After applying the first criterion, 12,745 variants were filtered, predominantly DELs and INSs. Out of these, 12,674 variants fell within the length range of 50 to 10K nucleotides. From this subset, 5,424 DELs were designated as 'TRUTH' variants for comparison with 'TEST' variants obtained from single algorithms and various combination strategies. We have revised this section to avoid misunderstanding.

***Page 6:***

**Methods**

**Truth set**

The GIAB v0.6 Tier 1 benchmark set [1] was obtained from ftp://ftp-trace.ncbi.nlm.nih.gov/ReferenceSamples/giab/data/AshkenazimTrio/analysis/NIST_SVs_Integration_v0.6/. *The HG00154, HG00733, and NA19240 benchmark set was obtained from HGSVC2 [25] (https://www.internationalgenome.org/data-portal/data-collection/hgsvc2).* To analyze performance, variants meeting specific criteria were selected as 'TRUTH' variants: 1*. variants with 'PASS' in the 'FILTER' column, 2. variants with lengths ranging from 50 to 10K base pairs, as we focused on SVs within this size range.* 3. variants of the SV type 'DEL' or *'INS', as we evaluated the performance of DELs and INSs separatively.* After applying the first criterion, *12,745 (HG002), 20,862 (HG00514), 21,189 (HG00733), and 24,318 (NA19240) variants were filtered respectively. Out of these, 12,674 (HG002), 20,762 (HG00514), 21,091(HG00733), and 24,212 (NA19240)* variants fell within the length range of 50 to 10K base pairs designated as 'TRUTH' variants for comparison with 'TEST' variants obtained from single algorithms and various combination strategies.

3. Mendelian Violation Experiment: Since the sample used for the benchmark is a son from a trio, performing a Mendelian violation experiment by comparing with the parents would add value.

**Response:**

Thank you for your comment! The GIAB truth set is based on the call set of trio analysis, and Mendelian violations were already considered in the original study of the GIAB truth set. Therefore, GIAB is highly commended for its comprehensiveness, as highlighted in a recent review by Sina Majidian et al. that discusses benchmark datasets, including those for medically relevant and complex genomic regions. Thus, we assessed the performance of each tool using this robust and well-validated truth set. Thank you again for your comment.

*Reference:*

- *Majidian, S., Agustinho, D.P., Chin, CS. et al. Genomic variant benchmark: Genomic variant benchmark: if you cannot measure it, you cannot improve it. Genome Biol 24, 221 (2023).*

4. Conclusion Alignment: The conclusion does not fully align with the title "Comprehensive evaluation and characterization of short read general-purpose structural variant calling software."

**Response:**

Thank you for your comment! We apologize for any confusion caused by unclear descriptions. The title 'Comprehensive evaluation and characterization of short read general-purpose structural variant calling software' is not our title; our actual title is 'Comparisons of Performances of Structural Variant Detection Algorithms in Solitary or Combination Strategies.'

We have revised the 'Conclusion' section and, following another reviewer’s suggestions, added three sample sets from the Human Genome Structural Variation Consortium 2 (HGSVC2). We have also incorporated the analysis of insertion (INS) detection into the 'Results' section. All figures, tables, and supplementary information have been thoroughly amended and reorganized. We hope the revision could make the conclusion aligns more closely with our title, “Comparisons of Performances of Structural Variant Detection Algorithms in Solitary or Combination Strategies.” Thank you again for your kindly reminder.

***Fig. 1***

***Fig 1. SV detection workflow.*** *SV detection workflow starting with benchmark set collection from GIAB and HGSVC2, followed by sequence alignment using BWA-MEM to produce BAM files. These BAM files are then processed by various SV callers (DRAGEN, Manta, DELLY, LUMPY, GRIDSS, SvABA) to generate VCF files, which are subsequently used for performance assessment, including single tool and combination strategies, based on recall, precision, and F1 score.*

*Fig. 2*

*Fig 2. The SVs detected by six SV callers. (A) SVs of all sizes detected by individual SV callers in samples HG002, HG00514, HG00733, and NA19240. (B) Length distribution of different variants for all samples detected by individual SV callers. The dashed line indicates the range from 50-10k base pairs. The maximum, minimum, and median based on the integrated values from all sample sets. DEL: deletion, INS: insertion, DUP: duplication, INV: inversion, and CTX: complex translocation.*

*Fig. 3*

*Fig 3. The distribution of SVs in truth sets and the performance of individual algorithms. (A) The distribution of SVs size and types in truth sets. The dashed line separates the regions above and below 10k base pairs. (B) The comparison of False negative (FN) and False positive (FP) numbers among individual algorithms. (C) The precision, recall, and F1 score of each individual algorithm in detecting “DELs” and “INSs” within the specific size range of 50-10k base pairs. DEL: deletion, INS: insertion.*

*Fig 4*

*Fig 4. Concordance of neighbor SVs detected by member callers in different combination strategies. (A*) *The agreement among three SV detection tools (Manta, DELLY, and GRIDSS). (B) The agreement among five SV detection tools (Manta, DELLY, GRIDSS, LUMPY, and SvABA).*

*Fig 5*

*Fig 5. Distribution and performance of combination strategies. (A) The distribution of SVs in different combination strategies. (B) The comparison of False negative (FN) and False positive (FP) numbers among individual algorithms. (C) The precision, recall, and F1 score of combination strategies in the detection of DELs and INSs within the specific size range of 50-10K base pairs. DEL: deletion, INS: insertions, DUP: duplication, INV: inversion.*

*Fig 6*

***Fig 6. Combination performance of each single caller and combination strategy in detecting DELs and INSs.*** *The maximum, minimum, and macro average of recall, precision, and F1 score based on the integrated values from all sample sets.*

5. Translocation Assessment: Why were translocations not included in the assessment?

**Response:**

Thank you for your comment! Translocations and inversions were not included in our assessment because the true sets does not provide benchmarks for these types of SVs. As a result, we excluded translocations and inversions from the comparisons in this study. We have clarified this point in our manuscript to explain why our analysis focused specifically on deletions and insertions in 'Methods' section. The distribution of SVs in true sets were also presented in Fig 3A and S5 Table. Additionally, we have referenced the work by Zook et al. (2020), the original study of GIAB benchmarkset. We appreciate your comment!

***Page 8:***

**Methods**

**Evaluation of performance for single algorithm and combination strategies**

The recall, precision, and F1 score for different single algorithms and combinations of multiple algorithms, either in “multiple agreement” strategy or union strategy, were calculated. We focused on the SV type 'DEL' and *'INS' within the range of 50 to 10k base pairs to analyze the performance of different single algorithms and various combination strategies, because the true sets only provide benchmarks for these types of SVs [1].*

***Fig 3A***

***S5 Table***

6. SvABA Reporting: Does SvABA report SVs in BND format? If so, how did you convert BND to other SV types?

**Response:**

Thank you for your comment! In our study, we employed the R package, which was developed by *Cameron DL* et al., to annotate the types and lengths of the identified SVs, including those SVs in BND format.

*Reference:*

- *Cameron DL, Dong R, Papenfuss AT. StructuralVariantAnnotation: a R/Bioconductor foundation for a caller-agnostic structural variant software ecosystem. Bioinformatics. 2022;38(7):2046-8. Epub 2022/02/09. doi: 10.1093/bioinformatics/btac042.*

7. Duplicate Records: Lines 147-148 mention that duplicated records will be removed. Under what conditions would a calling algorithm report duplicated records?

**Response:**

Thank you for your comment! We have provided an example file (S1 file) to more clearly explain the “duplicated records.” This section of ‘Methods‘ has been revised. In the output files generated by some structural variant (SV) callers (GRIDSS, SvABA, LUMPY, and Manta), there can be two separate records representing the same SV. One record typically shows the start position of the variant, while the other shows the end position. We removed the record that shows the end position, ensuring that there is only one record per unique SV.

***Page 7:***

**Methods**

**Calling and refinement of structural variations**

… *In Step 3, the output files generated by some SV callers (GRIDSS, SvABA, LUMPY, and Manta), there can be two separate records representing the same SV. One record typically shows the start position of the variant, while the other shows the end position. We removed the record that shows the end position, ensuring that there is only one record per unique SV, as visually depicted in S1 Fig.* Finally, Step 4 involved the selection of variants with a 'PASS' status in the 'FILTER' field....................

***S1 File***

8. Duplication and Insertion Reporting: Calling algorithms may report duplications as insertions. How do you handle this case if you only combine SVs of the same type?

**Response:**

In our study, we used an R package for SV annotation to determine the types and lengths of the identified SVs. All outputs from different callers were annotated using the same R package, and the SV types were defined based on the results provided by the R package, thus ensuring a relatively consistent setting. The criteria for combining SVs involve matching SV types across different tools. If different tools identify varying SV types, we categorize these findings as inconsistent.

Additionally, our findings indicate that the union combination strategy significantly improves recall and F1 scores, comparable to those achieved with commercial tools. In our study, we only evaluated the performance in detecting deletions (DELs) and insertions (INSs) due to the limitations of the truth set. Practically, when duplications are reported as insertions, using a union strategy ensures that these SVs are not filtered out, allowing for their accuracy to be validated through other tools.

Moreover, considering that most short reads range from 300 to 500 bp in length, we believe that SVs within this range can be sequenced and accurately identified. In clinical practice, post-processing tasks like filtering target genes using a virtual panel, along with additional validation through the Integrative Genomics Viewer (IGV) or methods such as PCR, are commonly used. Therefore, given these considerations, we believe that achieving a high recall rate to minimize false negatives (FNs) significantly enhances the utility of these findings in clinical settings. Thank you again for your insightful comments.

*Reference:*

*•	Cameron DL, Dong R, Papenfuss AT. StructuralVariantAnnotation: a R/Bioconductor foundation for a caller-agnostic structural variant software ecosystem. Bioinformatics. 2022;38(7):2046-8. Epub 2022/02/09. doi: 10.1093/bioinformatics/btac042.*

9. Size Range Limitation: Please explain why the comparison was limited to the range of 50 to 10k base pairs.

**Response:**

Thank you for your comment! Since the vast majority of SV sizes in the true set fall within the range of 50 to 10k base pairs, we only compared SVs within this size range. The distribution of SVs in true sets were presented in Fig 3A and S5 Table.

***Fig 3A***

***S5 Table***

*Minor comments*

10. To be precise, replace “Jewish” with “Ashkenazim”.

**Response:**

Thank you for kind reminder. We have replaced the“Jewish” with “Ashkenazim.”

***Page 12:***

**Results**

**Evaluation of SV detection methods based on the GIAB benchmark set and well referenced cell lines**

We next set out to determine the overall performance of SV callers using the sample HG002, for which a high-confidence SV call set aligned to the GRCh37 version has been established as a standard for SV evaluation [1]. This evaluation is based on the genomic data of the son (HG002) in a broadly consented ~~Ashkenazi Jewish~~ Ashkenazim trio, provided by the Genome in a Bottle Consortium (GIAB) [1].

11. The authors mention that long-read sequencing in clinical diagnostics is hindered by cost constraints, implying this study would cover this application. However, there is no coverage of this aspect in the manuscript.

**Response:**

We appreciate you pointing out this oversight. In the revised version of the manuscript, we have added sections to discuss long-read sequencing and explain why there is still a need for short-read sequencing-based SV detection.

Indeed, recent advancements in long-read technologies, such as Oxford Nanopore Technologies (ONT) and PacBio HiFi sequencing, have significantly increased accessibility and improved accuracy, helping to overcome some limitations of short-read sequencing. Despite advancements and cost reductions that have made long-read sequencing more accessible, significant challenges remain. These include not only higher costs, longer runtimes, scalability issues, and stringent sample quantity and quality requirements, but also important considerations regarding the utility of existing short-read data. For example, many biobanks host extensive archives of short-read data, with a persisting high demand for their reanalysis. The UK Biobank has sequenced the whole genomes of 150,119 participants using Illumina NovaSeq sequencing. Although the All of Us (AoU) project has re-sequenced some samples using long-read techniques, it has already released over 100,000 datasets from Illumina whole genome sequencing. Meanwhile, short-read whole-genome sequencing (WGS) has achieved high accuracy in detecting single nucleotide variants (SNVs) and remains widely utilized. The practical convenience of employing the same sequencing approach for both SNVs and structural variants (SVs) presents significant benefits.

Furthermore, the global market for short-read sequencing, valued at USD 5,341.3 million in 2022, is expected to grow at a compound annual growth rate of 18.46% from 2023 to 2030, driven by increasing demand for personalized medicine and companion diagnostics. Short-read technologies, capable of generating vast data volumes in a single run, are not only cost-effective but also user-friendly. These factors are anticipated to further drive the industry’s growth.

Therefore, while the advantages of long-read sequencing are evident, the need for an effective short-read-based detection strategy remains urgent, considering the time, costs, existing data, and varied clinical scenarios. A more comprehensive assessment of these technologies is still valuable to balance their application in different contexts, reinforcing the utility of short-read WGS for detecting both SNVs and SVs. As you suggest, evaluating the applications of both long and short-read sequencing continues to be a focal point of widespread attention and discussion, this topic has been discussed by researchers like Amarasinghe SL et al. and Mahmoud M as well.

*References:*

- *Halldorsson, B.V., Eggertsson, H.P., Moore, K.H.S. et al. The sequences of 150,119 genomes in the UK Biobank. Nature 607, 732–740 (2022). https://doi.org/10.1038/s41586-022-04965-x*
- *Mahmoud, M., Huang, Y., Garimella, K. et al. Utility of long-read sequencing for All of Us. Nat Commun 15, 837 (2024). https://doi.org/10.1038/s41467-024-44804-3*
- *Amarasinghe SL, Su S, Dong X, Zappia L, Ritchie ME, Gouil Q. Opportunities and challenges in long-read sequencing data analysis. Genome Biology. 2020;21(1):30. doi: 10.1186/s13059-020-1935-5.*
- *Mahmoud M, Gobet N, Cruz-Dávalos DI, Mounier N, Dessimoz C, Sedlazeck FJ. Structural variant calling: the long and the short of it. Genome Biology. 2019;20(1):246. doi: 10.1186/s13059-019-1828-7.*
- *Grand View Research. Market analysis report: Short-read Sequencing Market Size, Share & Trends Analysis Report By Product (Instruments, Consumables), By Workflow, By Application, By End-Use, By Region, And Segment Forecasts, 2023 – 2030. Retrieved from* [*https://www.grandviewresearch.com/industry-analysis/short-read-sequencing-market*](https://www.grandviewresearch.com/industry-analysis/short-read-sequencing-market)*.*

Thank you for your comment!

***Page 4:***

***Introduction***

*Recently, long-read technologies such as Oxford Nanopore Technologies (ONT) and PacBio HiFi sequencing have made significant advancements and become more accessible, offering higher accuracy to overcome the limitations of short-read sequencing [11]. However, despite these advancements and cost reductions making long-read sequencing more feasible, substantial challenges remain. These include not only higher costs, longer runtimes, scalability issues, and stringent requirements for sample quantity and quality[12, 13], but also important considerations for the vast amounts of existing short-read data. For instance, many biobanks contain extensive archives of short-read data, which continue to have a high demand for reanalysis. The UK Biobank, for example, has sequenced the whole genomes of 150,119 participants using Illumina NovaSeq sequencing [14]. Similarly, while the All of Us (AoU) project has re-sequenced some samples with long-read techniques, it has already released over 100,000 datasets from Illumina whole genome sequencing (WGS) [15] , highlighting the ongoing reliance on short-read approaches.*

***Page 27:***

**Discussions**

*Finally, when addressing SV calling, the role of long-read sequencing cannot be overlooked. Long-read sequencing offers significant advantages as it can span repetitive or other problematic regions. For applications that require identifying very long or complex SVs, performing a localized long-read de novo assembly is more effective than conventional short-read mapping approaches. However, despite the advancement of long-read technologies and the continuous cost reductions by both Oxford Nanopore and PacBio, which have made long-read sequencing increasingly widespread, the costs remain substantial. Additionally, algorithms based on long reads generally have longer runtimes compared to standard algorithms using tools like BWA [6]. A more comprehensive assessment of these technologies is still lacking. Considering time, costs, different clinical practical scenario and the vast amount of existing data, an urgent need remains for an effective short-read-based detection strategy.*

*Although our study only focused on the DELs and INSs— which are the majority but relatively simple SV types in the human genome— methods failing to detect DELs and INSs might also be less effective at identifying more complex SV types. Currently, our study evaluated the ability of various methods and combination strategies to detect DELs and INSs, providing a valuable and optimistic perspective on overall SV detection performance.*

12. Please spell out SNV and indel the first time they are used. Regarding SV, once it is defined, there is no need to define it every time it appears.

**Response:**

Thank you for your kind reminder. We have amended the text to spell out SNV (single nucleotide variant) and indel (insertion-deletion) the first time they are used. Additionally, we have ensured that SV (structural variant) is defined only once, with subsequent mentions using the abbreviation. Thank you for your valuable suggestion. All necessary changes have been made.

***Page 4:***

**Introductions**

Genomic *structural variants (SVs)* represent significant alterations in the genome, ranging in size from 50 bases to megabases base pairs. These variants include unbalanced gains and losses of DNA segments as well as balanced rearrangements. SVs are commonly classified into five types: deletions (DELs), insertions (INSs), duplications (DUPs), inversions (INVs), and complex translocations (CTXs) [1]. Due to their relatively large size, SVs account for a greater proportion of heritable sequence differences between individuals compared to *single nucleotide variants (SNVs),* with SVs constituting 0.5–1% of the genome and SNVs approximately 0.1% [2].

13. Clarify what you mean by “high-throughput analysis” in the third paragraph of the Introduction section (Line 78).

**Response:**

Thank you for your kind reminder. To avoid imprecise description, we have removed the section, and reorganized the 'Introduction' section.

14. Replace “paired short reads” with “paired-end short reads” (Line 139).

**Response:**

Thank you for your kind reminder. We have amended the text to replace "paired short reads" with "paired-end short reads."

***Page 7:***

**Methods**

**Calling and refinement of structural variations**

We obtained the whole-genome *paired-end short reads* of HG002 (NA24385) from the National Institute of Standards and Technology (https://www.nist.gov) and *HG00514, HG00733, and NA19240 were obtained from HGSVC2 (https://www.internationalgenome.org/data-portal/data-collection/hgsvc2).*

***Page 12:***

**Results**

**Evaluation of SV detection methods based on the GIAB benchmark set and well referenced cell lines**

....................We obtained the whole-genome *paired-end short reads* of HG002 from the National Institute of Standards and Technology (https://www.nist.gov).....................

15. Ensure consistent use of "nucleotide" or "base pair."

**Response:**

Thank you for your kind reminder. We have amended the text to ensure consistent usage throughout the manuscript. We uniformly use "base pairs" to maintain consistency.

16. The term “least POS” is unclear (Line 161).

**Response:**

Thank you for your kind reminder! In our study, 'POS' refers to the start position of a structural variant (SV). When referring to candidate SVs within a neighbor group, we retained the variant with the smallest start position as the representative. We have revised this section of ‘Methods’, and removed the term "least POS" to avoid ambiguity. Thank you so much for your comment!

***Page 8:***

**Methods**

**Combination strategies of multiple algorithms**

Individual SVs were annotated with the name of their respective SV detection algorithms in the 'INFOa' field (e.g., caller=DELLY). For grouping, one or more variants from different SV callers were considered a neighbor group if they existed within the range of +/- 500 bp of 'POS' *(start position)* and shared the same 'SVTYPE.' *When referring to candidate SVs within a neighbor group, we retained the variant with the smallest start position as the representative.*

17. The statement “We systematically searched scientific articles in PubMed” is ambiguous. Consider removing it.

**Response:**

Thank you for your kind reminder. Following your suggestion, we have removed this line.

***Page 6:***

**Methods**

We *~~conducted a systematic~~* search *~~of scientific articles in PubMed to identify~~* SV detection algorithms capable of handling short-read WGS through multiple methods....................

18. The Input and Output columns are identical for all callers, making them uninformative. Consider removing these columns.

**Response:**

Thank you for suggestion. We have removed these columns, and a revised Table 1 has been provided.

***Page 11:***

**Results**

19. Table S3 is redundant as its information is covered by Table S2.

**Response:**

Thank you for your kind comment. Following your suggestion, we have removed Table S3. Additionally, we have bolded the SV count size within the 50-100,000 range in S1-S5 Tables for clarity.

***S1 Table***

***S2 Table***

***S3 Table***

***S4 Table***
