# Supplementary material for: Comparisons of performances of structural variants detection algorithms in solitary or combination strategy
Source: PLoS One. 2025 Feb 6;20(2):e0314982. doi: 10.1371/journal.pone.0314982 (PMC11801633; doi:10.1371/journal.pone.0314982)
Supplement: Attachment — Submitted filename: point-by-point response letter to Plos One.docx [file pone.0314982.s016.docx]

Submission ID PONE-D-24-11846

**Comparisons of performances of Structural Variants Detection Algorithms in Solitary or Combination Strategy**

Point-by-point Response to the Editor and Reviewers

We greatly thank the editor and reviewers for the thoughtful comments of our manuscript. Following with all your kind suggestions, we have made improvement to our manuscript. The detailed point-by-point response to the comments are listed on the following pages. All the changes in revised manuscript are indicated by red color, and we also provide a clear version of our manuscript in the submission package. We really hope the revision will make the paper be more suitable for publication in ***PLOS ONE*** and look forward to hear from you in the near future. Your kind consideration will be highly appreciated!

**Editor**

*Comments to the Author*

Thank you for submitting your manuscript to PLOS ONE. After careful consideration, we feel that it has merit but does not fully meet PLOS ONE’s publication criteria as it currently stands. Therefore, we invite you to submit a revised version of the manuscript that addresses the points raised during the review process.

Please submit your revised manuscript by Oct 12 2024 11:59PM. If you will need more time than this to complete your revisions, please reply to this message or contact the journal office at [plosone@plos.org](mailto:plosone@plos.org). When you're ready to submit your revision, log on to <https://www.editorialmanager.com/pone/> and select the 'Submissions Needing Revision' folder to locate your manuscript file.

Please include the following items when submitting your revised manuscript:

- A rebuttal letter that responds to each point raised by the academic editor and reviewer(s). You should upload this letter as a separate file labeled 'Response to Reviewers'.
- A marked-up copy of your manuscript that highlights changes made to the original version. You should upload this as a separate file labeled 'Revised Manuscript with Track Changes'.
- An unmarked version of your revised paper without tracked changes. You should upload this as a separate file labeled 'Manuscript'.

If you would like to make changes to your financial disclosure, please include your updated statement in your cover letter. Guidelines for resubmitting your figure files are available below the reviewer comments at the end of this letter.

If applicable, we recommend that you deposit your laboratory protocols in [protocols.io](http://protocols.io) to enhance the reproducibility of your results. Protocols.io assigns your protocol its own identifier (DOI) so that it can be cited independently in the future. For instructions see: [https://journals.plos.org/plosone/s/submission-guidelines#loc-laboratory-protocols](https://c05y1x9s.r.us-east-2.awstrack.me/L0/https:%2F%2Fjournals.plos.org%2Fplosone%2Fs%2Fsubmission-guidelines%23loc-laboratory-protocols/1/010f0191996f7316-e1708288-fd63-49c7-bd3d-835b52c3008c-000000/rp0ipPBo_Juu4h4_aUL69s7zFiY=174). Additionally, PLOS ONE offers an option for publishing peer-reviewed Lab Protocol articles, which describe protocols hosted on [protocols.io](http://protocols.io). Read more information on sharing protocols at [https://plos.org/protocols?utm_medium=editorial-email&utm_source=authorletters&utm_campaign=protocols](https://c05y1x9s.r.us-east-2.awstrack.me/L0/https:%2F%2Fplos.org%2Fprotocols%3Futm_medium=editorial-email%26utm_source=authorletters%26utm_campaign=protocols/1/010f0191996f7316-e1708288-fd63-49c7-bd3d-835b52c3008c-000000/GPcveFbe4GUScjIlVIhIHKZDN3c=174).

**Response:**

We sincerely thank you for the insightful comments and constructive suggestions. We have carefully considered all the feedback and revised our manuscript accordingly. Below, we provide detailed responses to each comment and outline the changes made.

Additionally, we appreciate the recommendation regarding the use of protocols.io, and we will consider depositing our laboratory protocols there as suggested. We sincerely thank the editor again for the valuable feedback and consideration.

***Reviewers' comments:***

Reviewer's Responses to Questions

Comments to the Author

1. If the authors have adequately addressed your comments raised in a previous round of review and you feel that this manuscript is now acceptable for publication, you may indicate that here to bypass the “Comments to the Author” section, enter your conflict of interest statement in the “Confidential to Editor” section, and submit your "Accept" recommendation.

Reviewer #1: (No Response)

Reviewer #3: (No Response)

**Response:**

We appreciate the reviewers' detailed review.

2. Is the manuscript technically sound, and do the data support the conclusions?

Reviewer #1: Partly

Reviewer #3: Yes

**Response:**

We sincerely appreciate the detailed review and the valuable comments provided by the reviewers. Following the reviewers’ constructive suggestions, we have updated our analysis to include all sizes of structural variants (SVs) to further examine the performance of different individual SV callers and combination strategies. We also have calculated and included the ‘runtime’ for each tool used in this study, and this information has been incorporated into the revised manuscript.

We have revised our manuscript to make sure the conclusions are directly supported by the data presented. Furthermore, we have rewritten the ‘Discussion’ section and reorganized the ‘Introduction’ section, any speculative statements have been either revised or removed. All changes are highlighted in red in the revised manuscript for ease of review. We sincerely thank the reviewers once again for the constructive comments.

3. Has the statistical analysis been performed appropriately and rigorously?

Reviewer #1: Yes

Reviewer #3: Yes

**Response:**

We appreciate for your comment!

4. Have the authors made all data underlying the findings in their manuscript fully available?

Reviewer #1: Yes

Reviewer #3: Yes

**Response:**

We appreciate for your comment!

5. Is the manuscript presented in an intelligible fashion and written in standard English?

Reviewer #1: Yes

Reviewer #3: Yes

**Response:**

We appreciate for your comment!

6. Review Comments to the Author

**Response:**

We sincerely appreciate for the detailed review and valuable comment.

**Reviewer #1**

*Comments to the Author*

The manuscript describes a benchmark experiment comparing tools to perform detection of SVs from short read data. The authors performed important changes in the manuscript to address most of my previous concerns, especially those related to the use of HG002 as the only benchmark dataset. They also fixed most empty, vague and even incorrect phrases likely produced by the use of large language models for automated writing (I recommend avoiding the use of such models). Regarding novelty, I would assume that the manuscript is interesting for the journal editors, taking into account that it was send again for another round of review.

I have the following remaining comments for improvement of the study, the last two related to the support of some of the statements included now in the discussion:

*Major comments*

1. I do not see a point on removing variants longer than 10kbp from the analysis. The number of gold standard variants is small, and hence it will not affect importantly the calculations of recall. However, figure 2 suggest that there is a relatively large number of false positive variants in that size range. These false variants are important (especially if they are deletions) because they can be interpreted as important losses of DNA (and possibly genes) that could be falsely related to a trait. It would be great if the authors show an example of at least one of these predictions to rationalize why the software tools make mistakes in this size range.

**Response:**

We sincerely thank you for your valuable comments and constructive suggestions. In response to reviewer’s comment, we have updated our analysis to include all sizes of SVs, all SVs larger than 50 bp are included in the current analysis. The performance of different individual SV callers and combination strategies has also been re-analyzed. The figures, tables, and manuscript have all been updated accordingly to reflect these changes.

Regarding the false positive (FP) variants that the reviewer is concerned about, we sincerely appreciate your insight. We understand that this is an important and valuable issue that warrants further exploration. Our study demonstrated that the multi-agreement strategy resulted in fewer FPs in SV detection compared to DRAGEN and most single algorithms (except Manta, GRIDSS, and SvABA). We are particularly interested in FPs that are consistently detected by multiple callers.

As the primary objective of this study is to compare the performance of individual tools and combination strategies against the commercial tool DRAGEN, we examined FPs that were often misidentified by multiple tools. Specifically, we analyzed the FPs called by at least two algorithms in the HG002 dataset and compared them with DRAGEN. We differentiated FPs identified only by the multi-agreement strategy (M), only by DRAGEN (D), and those identified by both (Both), the FP SV size displaying the median and interquartile range were shown as S4_figure. We found that the majority of FPs are smaller than 10^3 base pairs. For DEL FPs, the M group had a median SV length of 326 bp, while the D group had a median of 120 bp. As for INSs, due to the poor recall of most tools, the number of FPs was quite low, with D group INS FPs having a median SV length of 81 bp. Considering that most short reads range from 300 to 500 bp in length, we believe they should be capable of accurately sequencing variants of this small size. In practice, using additional tools such as IGV or methods like PCR is common, helping to avoid concerns over false positives (FPs) falsely linked to a phenotype. We sincerely thank the reviewer for raising this important point.

Each tool has its own underlying principles and parameters for detecting SVs, as outlined in their respective publications, many employ hard filters—such as thresholds for variant quality, read depth—to reduce FPs. However, care is needed to balance filtering to avoid discarding true positives.

Furthermore, several studies shown that the occurrence of FPs in short-read sequencing for SV identification can be influenced by various factors, affecting overall precision. For example, Daniel L. Cameron et al. assessed the impact of sequence context and event size on the precision of 10 SV callers, evaluating these tools using simulation data and well-referenced cell lines. Their study included several tools that we also selected, such as Manta, DELLY, GRIDSS, and LUMPY. They identified several major factors impact the precision: 1) The proximity of single-nucleotide variants (SNVs) or indels near the SV breakpoints. When multiple flanking variants are present, false positive rates increase. 2) SVs located in low-complexity and simple tandem repeat regions are prone to higher false positive rates. SVs within LTR, LINEs and SINEs regions also show reduced precision, leading to more false positives. 3) The size of the event also plays a role; they indicated SVs smaller than 100 bp are particularly challenging.

As the reviewer rightly mentioned, SV detection using long reads is more accurate, especially in repeat regions. A longer read can span the entire repeat region, addressing many challenges in SV detection. Our study, focusing on short-read callers, observed that tools incorporating assembly (AS) algorithms like Manta, GRIDSS, and SvABA had the fewest FPs across all datasets. We sincerely thank the editor and reviewers for their previous revision suggestions. Building on this foundation, we aim to further improve precision by incorporating machine learning (ML) based tools in the future, as this is also an aspect that many existing comparative studies lack. We believe that ensemble approaches, combined with careful tool selection and context-aware interpretation, can mitigate certain FP issues.

Following your kind suggestion, we have thoroughly revised and added a discussion on regarding FPs and the potential to further enhance precision through ML in the 'Discussion' section. Thank you again for your insightful comments.

Referecne:

- Cameron, D.L., Di Stefano, L. & Papenfuss, A.T. Comprehensive evaluation and characterisation of short read general-purpose structural variant calling software. *Nat Commun* 10, 3240 (2019). https://doi.org/10.1038/s41467-019-11146-4
- Zheng Y, Shang X (2024) SVvalidation: A long-read-based validation method for genomic structural variation. PLOS ONE 19(1): e0291741. https://doi.org/10.1371/journal.pone.0291741

***Page 11-24:***

**Results**

Thoroughly Revision.

***Page 25:***

**Discussion**

...... *As for the concern of FP variants that may could be falsely related to a phenotype. We are particularly interested in FPs that are consistently detected by multiple callers. We analyzed the FPs called by at least two algorithms in the HG002 dataset and compared them with DRAGEN. We differentiated FPs identified only by the multi-agreement strategy (M), only by DRAGEN (D), and those identified by both (Both), the FP SV size displaying the median and interquartile range were shown as S4_figure. We found that the majority of FPs are smaller than 10^3 base pairs. For DEL FPs, the M group had a median SV length of 326 bp, while the D group had a median of 120 bp. As for INSs, due to the poor recall of most tools, the number of FPs was quite low, with D group INS FPs having a median SV length of 81 bp. Considering that most short reads range from 300 to 500 bp in length, we believe they should be capable of accurately sequencing variants of this small size. Each tool has its own underlying principles and parameters for detecting SVs, as outlined in their respective publications, many employ hard filters—such as thresholds for variant quality, read depth—to reduce FPs. However, care is needed to balance filtering to avoid discarding true positives.* In clinical practice, post-processing tasks like filtering target genes using a virtual panel, along with additional validation through the Integrative Genomics Viewer (IGV) or methods such as PCR, are commonly used. Therefore, weighing these considerations, we believe that achieving a high recall rate to minimize FN enhance the utility of these findings in clinical settings.

*Several studies also shown that the occurrence of FPs in short-read sequencing for SV identification can be influenced by various factors, affecting overall precision. For example, Daniel L. Cameron et al. assessed the impact of sequence context and event size on the precision of 10 SV callers, evaluating these tools using simulation data and well-referenced cell lines. Their study included several tools that we also selected, such as Manta, DELLY, GRIDSS, and LUMPY. They identified several major factors impact the precision: 1) The proximity of single-nucleotide variants (SNVs) or indels near the SV breakpoints. When multiple flanking variants are present, false positive rates increase. 2) SVs located in low-complexity and simple tandem repeat regions are prone to higher false positive rates. SVs within LTR, LINEs and SINEs regions also show reduced precision, leading to more false positives. 3) The size of the event also plays a role; they indicated SVs smaller than 100 bp are particularly challenging, whereas deletions between 300 to 500 bp tend to achieve higher precision.*

Finally, when addressing SV calling, the role of long-read sequencing cannot be overlooked. Long-read sequencing offers remarkable advantages as it can span repetitive or other problematic regions. For applications that require identifying very long or complex SVs, performing a localized long-read de novo assembly is more effective than conventional short-read mapping approaches. *Multiple studies have demonstrated that long-read sequencing technologies offer superior accuracy in SV detection compared to short-read approaches. Benchmarking results, as shown in recent studies [31, 39, 40] , consistently highlight the advantages of long reads in terms of sensitivity and specificity, regardless of the specific tools used. Moreover, the recent advancements in long-read technologies and the ongoing reductions in sequencing costs—led by both Oxford Nanopore and PacBio—have made long-read sequencing increasingly accessible and widespread. Integrating short-read and long-read sequencing provide opportunities for developing strategies tailored to diverse research and clinical needs.*......

***Fig. 2***

**Fig 2. The SVs detected by six SV callers. (A*)*** *SVs of sizes ≥ 50 bp and CTX detected by individual SV callers in samples,* HG002, HG00514, HG00733, and NA19240. **(B)** Length distribution of different variants for all samples detected by individual SV callers. *~~The dashed line indicates the range from 50-10k base pairs.~~* The maximum, minimum, and median are based on the integrated values from all sample sets. DEL: deletion, INS: insertion, DUP: duplication, INV: inversion, and CTX: complex translocation.

***Fig. 3***

**Fig 3. The distribution of SVs in truth sets and the performance of individual algorithms. (A)** The distribution of SVs size and types in truth sets. **(B)** The comparison of False negative (FN) and False positive (FP) numbers among individual algorithms. **(C)** The precision, recall, and F1 score of each individual algorithm in detecting “DELs” and “INSs” *with the size ≥ 50 bp*. DEL: deletion, INS: insertion.

***Fig. 4***

**Fig 4. Concordance of neighbor SVs detected by member callers in different combination strategies. (A)** The agreement among three SV detection tools (Manta, DELLY, and GRIDSS). **(B)** The agreement among five SV detection tools (Manta, DELLY, GRIDSS, LUMPY, and SvABA).

***Fig. 5***

**Fig 5. Distribution and performance of combination strategies. (A)** The distribution of SVs in different combination strategies. **(B)** The comparison of False negative (FN) and False positive (FP) numbers among individual algorithms. **(C)** The precision, recall, and F1 score of combination strategies in the detection of DELs and INSs. DEL: deletion, INS: insertions, DUP: duplication, INV: inversion.

***Fig. 6***

**Fig 6. Combination performance of each single caller and combination strategy in detecting DELs and INSs.** The maximum, minimum, and macro average of recall, precision, and F1 score are based on the integrated values from all sample sets.

***S4 Fig.***

***S4. Fig. Length distribution of FP SVs.*** *FPs identified only by the multi-agreement strategy (M), only by DRAGEN (D), and those identified by both (Both), in the HG002 dataset. The FP SV size displaying the median and interquartile range were shown.*

2. I do not understand why variants with lengths smaller than 50bp are filtered out for all tools except GRIDSS. This makes unnecessarily complicated the interpretation of the number of variants produced by each tool. In figure 2, Figure 2A will look much better if small variants reported by GRIDSS are filtered out. In figure 2B it is not clear why different scales are used in the Y axis for the different tools if all tools report numbers within the same orders of magnitude (once the results of GRIDSS are fixed).

**Response:**

We sincerely thank you for your valuable comments and constructive suggestions. Following your kind recommendation, we have filtered out small variants (< 50 bp) across all tools and all the type and size of SVs ($\geq$ 50 bp) detected by each individual algorithm are now presented in the revised Figure 2A. Additionally, the Y-axis scale in Figure 2B has been standardized across all tools for consistency. S1-S4 tables have also been updated accordingly to reflect these changes. We sincerely thank the reviewer once again for their valuable comments.

***Page 13:***

**Results**

...…..*The detection performance of the six SV callers varied, as shown in Figure 2 and S1-S4 Tables. For HG002, DRAGEN exhibited the highest number of detected SVs (10,912), followed by Manta (7,758), DELLY, LUMPY, SvABA, and GRIDSS. The other three datasets obtained from HGSVC2 showed a similar pattern in the number of detected SVs for each caller, as seen in HG002 (Figure 2A), except for LUMPY, which detected a higher number of CTX SVs in the HGSVC2 datasets.*

We also analyzed the distribution of SV sizes detected by various algorithms. Notably, the SV size distribution varied among the different algorithms (Fig 2B and S1-S4 Tables). GRIDSS identified a *~~significant~~ large number of small indels (S1-S4 Tables, S3_Fig),* while DELLY and LUMPY, despite both employing SR and RP algorithms, showed variations in their detected SV sizes and types. The SVs identified by DRAGEN, Manta, and SvABA primarily ranged from 50 to 10K base pairs (Fig 2B and S1-S4 Tables)……..

***Fig. 2***

*Fig 2. The SVs detected by six SV callers. (A) SVs of sizes ≥ 50 bp and CTX detected by individual SV callers in samples, HG002, HG00514, HG00733, and NA19240. (B) Length distribution of different variants for all samples detected by individual SV callers. ~~The dashed line indicates the range from 50-10k base pairs.~~ The maximum, minimum, and median based on the integrated values from all sample sets. DEL: deletion, INS: insertion, DUP: duplication, INV: inversion, and CTX: complex translocation.*

3. Referring to detection of SVs from long reads, the authors mention in the discussion that “A more comprehensive assessment of these technologies is still lacking”. First of all, it is not clear compared to what refers the term “more comprehensive”. More importantly, the statement is arguably false. All papers presenting new tools for SV detection from long reads include benchmark experiments (see dois 10.1093/gigascience/giad112, 10.1186/s13059-020-02107-y, and 10.1038/s41592-018-0001-7 for example ). Comparing to the accuracies presented in this work on the same benchmark datasets, it is clear that SV detection from long reads is much more accurate than SV detection from short reads, regardless the tool used for analysis. This should be clearly mentioned in the discussion, referring to the appropriate papers.

**Response:**

We sincerely appreciate your detailed review and the references provided. We apologize for any ambiguous statements and for any confusion they may have caused. In response to the reviewer's comments, we have revised the discussion. We acknowledge that SV detection from long reads is more accurate than SV detection from short reads, as demonstrated in multiple recent benchmark studies. The references (DOIs: 10.1093/gigascience/giad112, 10.1186/s13059-020-02107-y, 10.1038/s41592-018-0001-7) provided by the reviewer have been incorporated into the revised discussion. Thank you again for your valuable suggestion.

***Page 26:***

**Discussion**

......Finally, when addressing SV calling, the role of long-read sequencing cannot be overlooked. Long-read sequencing offers remarkable advantages as it can span repetitive or other problematic regions. For applications that require identifying very long or complex SVs, performing a localized long-read de novo assembly is more effective than conventional short-read mapping approaches. *Multiple studies have demonstrated that long-read sequencing technologies offer superior accuracy in SV detection compared to short-read approaches. Benchmarking results, as shown in recent studies [31, 39, 40] , consistently highlight the advantages of long reads in terms of sensitivity and specificity, regardless of the specific tools used. Moreover, the recent advancements in long-read technologies and the ongoing reductions in sequencing costs—led by both Oxford Nanopore and PacBio—have made long-read sequencing increasingly accessible and widespread. Integrating short-read and long-read sequencing provide opportunities for developing strategies tailored to diverse research and clinical needs.*......

4. The authors mention in the discussion that the analysis of long reads takes a longer runtime compared to the analysis of short reads, referring to bwa. This is likely a wrong statement. First of all, it is not clear from the text why bwa is mentioned, taking into account that bwa would be the mapping tool and hence it should be compared to tools such as minimap. More importantly, the authors do not provide any result on computational efficiency of the different methods. Since this is actually an important outcome of every benchmark experiment, the authors should include a comparison of computational efficiency among the methods considered in this study. It is understandable that the runtime of their merging strategies will be at least the sum of the runtimes of the individual variant callers. If the authors want to make a statement comparing computational efficiency between the analysis of short and long reads, then they should provide comparisons including software to detect SVs from long reads.

**Response:**

We appreciate the reviewer's insightful comment. In response to the reviewer's comments, we have removed the statement regarding the runtime of long-read sequencing analysis from the discussion.

Additionally, following reviewer’s suggestion, we have now calculated and included the runtime for tools used in this study. These results are presented in S2_Figure and revised manuscript. We sincerely thank you for your valuable comments.

***Page 24:***

**Discussion**

Thoroughly Revision.

***Page 17:***

**Results**

......*The relative runtime and maximum memory usage of each individual SV caller were tested using three HGSVC2 datasets and are presented in S2_figure. Total CPU time reflects the overall CPU usage, while wall time represents the actual elapsed time. Wall time can be shorter than total CPU time when parallelization is efficiently utilized. Among the non-commercial tools, Manta exhibited the lowest run time and maximum memory usage across all datasets, whereas GRIDSS required the most elapsed time and memory*......

***S2 Fig.***

***S2. Fig.*** ***Runtime performance of SV callers.*** *Runtime performance of each tool was tested on an isolated virtual machine, Taiwania 3, a high-performance computing platform featuring 900 compute nodes, each equipped with dual Intel Xeon Platinum 8280 processors (2.4 GHz, 28 cores per processor) and 192 GB of main memory. The system runs on CentOS 7.8 and utilizes the Slurm workload manager for resource scheduling. Taiwania 3 employs 100 Gbps InfiniBand HDR100 high-speed network connectivity, delivering a total computing power of up to 2.7 PFLOPS. We use node ngs92G, with total memory: 92 GB and total CPU cores: 14. Total CPU time indicates the overall CPU utilization, while wall time represents the real total elapsed time. Wall time can be less than the total CPU time if the process is executed efficiently, utilizing parallelization.*

________________________________________________________________________________

I have some additional minor comments (line numbers refer to the version with control change):

*Minor comments*

Pages 15, 17 and 20: I think it is not worth to spell every single value of true positives, false positives and false negatives in the text, especially if you also have tables in the main text with the values. You could use ranges and only report the most important numbers that you want to highlight.

**Response:**

We appreciate the reviewer's suggestion. As recommended, we now present some of the data using ranges instead of listing individual values, and we have highlighted the best results in bold and the worst results with underlines for clarity in the tables. Furthermore, based on your additional recommendations, we have revised our analysis to include all SVs larger than 50 bp. The manuscript has been thoroughly updated. Again, we sincerely thank you for your valuable comments.

***Page 15:***

**Results**

**The individual performance of each algorithm**

......We considered SVs if they were located within a range of +/-500 base pairs of the 'POS' (start position) and were of the same SV type as the truth set SVs. Fig 3 and Table 2 present the detailed performance of each single algorithm. *In HG002, for DELs, DRAGEN performed the best, identifying 3,425 true positives (TPs), followed by Manta, DELLY, GRIDSS, and SvABA, with LUMPY identifying the fewest. As for total error (FP + FN), DRAGEN and Manta also had the lowest error rates, while LUMPY had the highest. Similar trends were observed across other HGSVC2 datasets (Fig 3B and Table 2).*

*For INSs, DRAGEN and Manta again led in TP counts, while DELLY and GRIDSS had lower FPs but unacceptably high false negatives (FNs) across all datasets. (Fig 3B and Table 2).*......

***Page 17:***

**Results**

**The individual performance of each algorithm**

......For each distinct algorithm, we computed the recall, precision, and F1 score. *As shown in Fig 3C and Table 2, for DELs across all benchmark sets, DRAGEN achieved the highest F1 scores (0.64–0.68), followed closely by Manta, DELLY, and GRIDSS. LUMPY scored the lowest F1 in HG002 (0.19), while SvABA had mid-range scores in HG002 but lower in other datasets. Among all non-commercial software, Manta had the highest recall (0.43–0.55%), with GRIDSS showing lower recall but higher precision (0.86–0.95).*

*We also conducted a performance analysis of INSs, but most single algorithms performed disappointingly. Across all datasets, DRAGEN achieved the highest F1 scores (0.45–0.55), followed by Manta. DELLY (0.02–0.04) and GRIDSS (0.01–0.02) had very unsatisfactory F1 scores. Among non-commercial software, Manta had the highest recall (0.16–0.19) but only moderate precision (0.77–0.96). In contrast, GRIDSS and DELLY showed better precision (0.91–0.99) but poor recall (0.00–0.02%). Overall, low recall impacted the performance of these tools (Fig 3C and Table 2).......*

***Page 19:***

**Results**

**Systematic identification of the performances of combination strategies**

......*For DELs in HG002, DRAGEN had the highest TP count (3,425), followed by V-union, III-union, V-multiple agreement, and the lowest for III-multiple agreement (2,554). The FP count was lowest for III-multiple agreement (533) and highest for V-union (2,199). Similar results were observed in the HGSVC2 datasets. Regarding total errors (FP + FN), five-algorithm strategies generally produced more errors than three-algorithm strategies. For INSs, DRAGEN had the highest TP counts, while multiple agreement had the highest total errors. Overall, combination strategies still struggled with a high FN rate (Fig 5B and Table 3).*

*For each combination strategy, we calculated recall, precision, and the F1 score. As shown in Fig 5C and Table 3, for DELs across sample sets, DRAGEN achieved the highest F1 score (0.64–0.68), followed by III-union and V-union, with III-multiple and V-multiple agreement scoring lower. DRAGEN had the highest recall, closely followed by the union groups (0.50–0.62), while multiple agreement groups had lower recall (0.33–0.48). In contrast, precision was higher in the multiple agreement groups (0.74–0.93) and lower in the union groups (0.61–0.76).*

*For INSs, the performance of combination strategies was also disappointing across all benchmark sets (Fig 5 and Table 3). DRAGEN achieved the highest F1 score (0.45–0.55), followed by the union groups (0.28–0.31), with the multiple agreement groups demonstrated the worst performance (0.02–0.04. Increasing the number of tools from three to five did not improve performance, regardless of whether the multiple agreement or union strategies were used*.

1. Line 24: Why the role of SVs is “pivotal”

**Response:**

We sincerely thank you for your thoughtful comments. Research indicates that SVs play a crucial role in genomic variation, with their contribution being higher (1.5%) compared to single nucleotide variants (0.1%). Their impact on gene function, disease mechanisms, and evolutionary processes underscores their importance in understanding genetic diversity and disease. To address reviewer’s concern about the term "pivotal," we have revised the sentence. In response to another reviewer’s suggestion, the 'Abstract' section has been thoroughly revised. Again, we sincerely thank you for your valuable comments.

Reference: Mahmoud, M., Gobet, N., Cruz-Dávalos, D.I. *et al.* Structural variant calling: the long and the short of it. *Genome Biol* **20**, 246 (2019). https://doi.org/10.1186/s13059-019-1828-7

***Page 2:***

***Abstract***

Structural variants (SVs) *have been associated with changes in gene expression, which may contribute to alterations in phenotypes and disease development.* However, the precise identification and characterization of SVs remain challenging. While long-read sequencing offers superior accuracy for SV detection, short-read sequencing remains essential due to practical and cost considerations, as well as the need to analyze existing short-read datasets. *~~To address this, Multiple methodologies targeting different SV classes, zygosities, and size ranges have been developed.~~ ~~Detecting SVs using short-read sequencing data is particularly challenging, often leading to low sensitivity and high false discovery rates.~~ Numerous algorithms for short-read SV detection exist, none are universally optimal, each having limitations for specific SV sizes and types. In this study, we evaluated the efficacy of six advanced SV detection algorithms, including the commercial software DRAGEN, using the GIAB v0.6 Tier 1 benchmark and HGSVC2 cell lines. We employed both individual and combination strategies, with systematic assessments of recall, precision, and F1 scores. Our results demonstrate that union combination approach enhanced detection capabilities, surpassing single algorithms in identifying deletions and insertions, delivered comparable recall and F1 scores to the commercial software DRAGEN. Interestingly, expanding the number of algorithms from three to five in the combination did not enhance performance, highlighting the efficiency of a well-chosen ensemble over a larger algorithmic pool.*

2. Line 50: Base pairs is written twice

**Response:**

Thank you for pointing that out. We have corrected the duplication of "base pairs".

***Page 3:***

***Introduction***

Genomic structural variants (SVs) are substantial alterations in the genome, varying in size from 50 to mega base pairs (bp) *~~base pairs~~*.*…….*

3. Line 82. A noun is missing before “… become more accessible”

**Response:**

We sincerely thank you for pointing that out. We have updated the sentence to improve clarity.

***Page 4:***

***Introduction***

*Recently, long-read technologies such as Oxford Nanopore Technologies (ONT) and PacBio HiFi sequencing have made remarkable advancements, becoming more accessible and offering higher accuracy. These improvements help overcome many of the limitations associated with short-read sequencing [12]……*

4. Line 94: It is not clear which is the “sector” mentioned in the sentence.

**Response:**

We sincerely thank you for pointing that out. Following your comment, we have revised the sentence for clarity.

***Page 4:***

***Introduction***

*Today, there has been substantial progress in short-read-based general-purpose SV detection, with numerous SV detection algorithms being published…….*

5. Line 99: A simple sentence should not start with “Although”.

**Response:**

We appreciate the reviewer's attention to detail. The sentence has been revised. In response to another reviewer’s suggestion, the ‘Introduction’ section has been reorganized in the revised manuscript. Thank you for pointing this out.

***Page 4:***

***Introduction***

*Today, there has been substantial progress in short-read-based general-purpose SV detection, with numerous SV detection algorithms being published.* Many projects use popular SV detection algorithms showed a relatively high accuracy [17-19]. *Although sequencing-based methods theoretically detect any type of SV, no single algorithm can accurately and sensitively detect all types and sizes [20-22]. Several studies have highlighted the individual performance of various callers. For example, Varuni Sarwal et al. [23] evaluated the performance of SV detection tools on mouse and human WGS data, focusing on DELs detection using a comprehensive set confirmed by polymerase chain reaction (PCR) and the GIAB benchmark set.*

*Evaluations of combination strategies, however, remain relatively scarce…….*

6. Line 105. The a-priori qualitative statement about SvABA probably needs a reference, especially taking into account that later SvABA turns out to be one of the worst tool in the comparison.

**Response:**

We appreciate the reviewer's insightful comment. In response, we have added relevant citations. Indeed, in our results, SvABA demonstrated lower overall performance compared to other tools, but it exhibited high precision.

Initially, in selecting SV callers for our analysis, we chose tools based on their performance in recent benchmarking studies, focusing particularly on a combined algorithm approach and their deletion detection capabilities. According to the literatures, SvABA has demonstrated high accuracy across various SV sizes, particularly for DELs [ref below: 1-3]. Although SvABA is not the best, it shows a moderate level of performance for deletion detection compared to other SV callers using a combined algorithm approach [ref below: 4], which contributed to our decision to include it in our study. We sincerely thank the reviewer again for the valuable comments.

References:

1. Gong, T., Hayes, V.M., Chan, E.K.F. "Detection of somatic structural variants from short-read next-generation sequencing data." Briefings in Bioinformatics. 2021, Volume 22, Issue 3. DOI: 10.1093/bib/bbaa056.
2. Zhuang, G., Zhang, X., Du, W., Xu, L., Ma, J., Luo, H., Tang, H., Wang, W., Wang, P., Li, M., Yang, X., Wu, D., Fang, S. "A benchmarking framework for the accurate and cost-effective detection of clinically-relevant structural variants for cancer target identification and diagnosis." J Transl Med. 2024 Jan 16; 22(1):65. DOI: 10.1186/s12967-024-04865-w.
3. Joe, S., Park, J.L., Kim, J., et al. "Comparison of structural variant callers for massive whole-genome sequence data." BMC Genomics. 2024, 25:318. DOI: 10.1186/s12864-024-10239-9.
4. Kosugi, S., Momozawa, Y., Liu, X., et al. "Comprehensive evaluation of structural variation detection algorithms for whole genome sequencing." Genome Biol. 2019. DOI: 10.1186/s13059-019-1720-5.

***Page 5:***

***Introduction***

In a benchmarking study assessing 46 SV callers published between 2009 and 2017 [24], DELLY [17], LUMPY [18], Manta [19], and GRIDSS [25] were identified as popular tools based on their citation counts in the Web of Science database. While SvABA [26] was not included due to its 2018 publication date*, it has shown robust performance in detecting both germline and somatic SVs in other assessments [27].*

7. Line 147 - 149: The first sentence is written in active voice and the second sentence is written in passive voice. Either one can be use but do not mix up.

**Response:**

We sincerely thank you for pointing that out. We have revised the sentence to maintain consistency in voice.

***Page 7:***

**Materials and Methods**

**Calling and refinement of structural variations**

*We obtained the whole-genome paired-end short reads of HG002 (NA24385) from the National Institute of Standards and Technology (https://www.nist.gov), and HG00514, HG00733, and NA19240 from HGSVC2 (https://www.internationalgenome.org/data-portal/data-collection/hgsvc2). These raw reads were aligned to the GRCh37 (for HG002) or GRCh38 (for HG00514, HG00733, and NA19240) versions of the human reference genome using BWA-MEM, as the SV benchmark set was based on the references. Subsequently, the resulting BAM files were processed through various SV detection algorithms, utilizing their default parameters as recommended by the authors for calling SVs....…*

8. Line 161 - 163: The sentence is confusing. It may be better to explain first the issue and then the solution.

**Response:**

We sincerely thank you for the valuable suggestion. We apologize for any unclear statements made and the confusion they may have brought about. We have revised this paragraph.

***Page 7:***

***Materials and Methods***

**Calling and refinement of structural variations**

*....…For the refinement, filtering, and deduplication, the variants underwent a systematic four-step process: 1) SV Annotation: we employed the R package [30] to annotate SV types and lengths. 2) Size Exclusion: SVs smaller than 50 bp were excluded. 3) Record Deduplication: In cases where some SV callers (GRIDSS, SvABA, LUMPY, and Manta) generated output file containing separate records for the start and end of the same SV, we removed the end position record, as shown in S1 Fig. 4) Final Filtering: Only variants marked 'PASS' in the 'FILTER' field were retained for further analysis.*

9. Line 201. strategies is misspelled

**Response:**

We appreciate you pointing that out and apologize for the misspelling. The word "strategies" has been corrected in the revised text. Additionally, in response to another reviewer's suggestion, all formulas in the manuscript have now been numbered for reference and citation purposes.

***Page 8:***

***Materials and Methods***

**Evaluation of performance for single algorithm and combination strategies**

The recall, precision, and F1 score for different single algorithms and combinations of multiple algorithms, either in “multiple agreement” strategy or union strategy, were calculated. We focused on the SV types 'DEL' and 'INS' *~~within the range of 50 to 10k base pairs~~* to analyze the performance of different single algorithms and various combination strategies, because the truth sets only provide benchmarks for these types of SVs [1]. *Variants detected by single algorithms and combination strategies (referred to as 'TEST' sets) were compared against the truth sets.*

For evaluation, the true positive (TP) was defined if a variant in the 'TRUTH' set was located within the range of +/-500 bp of the 'POS' (start position) of a variant in the 'TEST' set and shared the same SV type. False positives (FP) were calculated as the total variant count of the 'TEST' set minus TP, and false negatives (FN) were calculated as the total variant count of the 'TRUTH' set minus TP. Precision, recall, and F1 score were then calculated using the following formulas.

$\text{precision=}\frac{\text{TP}}{\text{TP + FP}}$ (1)

$recall=\frac{\mathrm{TP}}{TP+FN}$ (2)

$F1=2\times\frac{precison\times recall}{precision+recall}$ (3)

To determine the rank of overall performance of each single caller and combination strategies, combined precision score (cPr), combined recall score (cRc), and combined F1 score (cF1), in which the values for all four data sets were integrated, and were calculated by micro average as follows:

$\text{cPr=}\frac{\text{TP1+TP2+TP3+TP4}}{\text{TP1+TP2+TP3+TP4 + FP1+FP2+FP3+FP4}}$ (4)

$cRc=\frac{\text{TP1+TP2+TP3+TP4}}{\text{TP1+TP2+TP3+TP4}+\text{FN1+FN2+FN3+FN4}}$ (5)

$cF1=2\times\frac{cPr\times cRc}{cPr+cRc}$ (6)

10. Line 246. Please explain in the methods how “state-of-the art” is defined and evaluated as a software quality attribute to select software tools.

**Response:**

We sincerely thank the reviewer for the valuable comments. Initially, in selecting SV callers for our analysis, we chose tools based on their performance in recent benchmarking studies, with a particular focus on a combined algorithm approach and deletion detection capabilities. Our selection criteria prioritized tools that demonstrated superior performance and widespread recognition in these studies. We also evaluated the quality of software documentation, including the comprehensiveness of tutorials or manuals, and considered the practical aspects of using these tools on our system. To avoid confusion and provide greater clarity, we have removed the term "state-of-the-art" from the manuscript and revised the relevant section. We thank the reviewer again for the valuable comments.

References:

1. Mu, W., Li, B., et al. "Detection of structural variation using target captured next-generation sequencing data for genetic diagnostic testing." Genet Med. 2019. DOI: 10.1038/s41436-018-0397-6.
2. Gong, T., Hayes, V.M., Chan, E.K.F. "Detection of somatic structural variants from short-read next-generation sequencing data." Briefings in Bioinformatics. 2021, Volume 22, Issue 3. DOI: 10.1093/bib/bbaa056.
3. Zhuang, G., Zhang, X., Du, W., Xu, L., Ma, J., Luo, H., Tang, H., Wang, W., Wang, P., Li, M., Yang, X., Wu, D., Fang, S. "A benchmarking framework for the accurate and cost-effective detection of clinically-relevant structural variants for cancer target identification and diagnosis." J Transl Med. 2024 Jan 16; 22(1):65. DOI: 10.1186/s12967-024-04865-w.
4. Joe, S., Park, J.L., Kim, J., et al. "Comparison of structural variant callers for massive whole-genome sequence data." BMC Genomics. 2024, 25:318. DOI: 10.1186/s12864-024-10239-9.
5. Kosugi, S., Momozawa, Y., Liu, X., et al. "Comprehensive evaluation of structural variation detection algorithms for whole genome sequencing." Genome Biol. 2019. DOI: 10.1186/s13059-019-1720-5.

***Page 11:***

**Results**

**SV detection methods included in this study**

*We first reviewed previous literature on short-reads SV callers, evaluated criteria such as superior performance, widespread recognition, active development, and software/documentation quality, as well as their ease of use on our system. Ultimately, we selected five SV detection algorithms that demonstrated good performance in recent benchmarking studies [7, 27, 31-33],* namely DELLY [17], LUMPY [18], Manta [19], GRIDSS [25], and SvABA [26]…….

11. Line 304. Please provide an statistical test and p-value to assess why the number of short variants reported by GRIDSS is “significant”. Please explain compared to what the number is significantly higher or smaller.

**Response:**

Thank you for your comment. We apologize for any confusion. The term "significant" in this context was not intended to imply statistical significance. We have removed all instances of "significant" that were not used in a statistical context to avoid any misunderstanding. We also provide a statistical test and p-value to assess the significance of the number of short variants reported by GRIDSS, detailed in S3_file.

***Page 13:***

**Results**

**Evaluation of SV detection methods based on the GIAB benchmark set and well referenced cell lines**

We also analyzed the distribution of SV sizes detected by various algorithms. Notably, the SV size distribution varied among the different algorithms (Fig 2B and S1-S4 Tables). *GRIDSS identified a ~~significant~~ large number of small indels (S1-S4 Tables, S3_Fig),* while DELLY and LUMPY, despite both employing SR and RP algorithms, showed variations in their detected SV sizes and types. The SVs identified by DRAGEN, Manta, and SvABA primarily ranged from 50 to 10K base pairs (Fig 2B and S1-S4 Tables). Each algorithm detected DELs, DUPs, and INVs. Although all algorithms identified INSs, LUMPY and SvABA showed particularly poor performance in detecting these variants. CTXs were detected by all algorithms except DELLY (Fig 2 and S1-S4 Tables). The distribution of SV types was consistent across the datasets analyzed.

***S3. Fig.***

**S3. Fig. Efficacy of GRIDSS in detecting small variants compared to other SV detection tools.** The comparative effectiveness of GRIDSS in detecting small variants compared to other SV detection tools. *p<0.0001.

12. Lines 340 - 342: Given that the gold standard is a fixed dataset, the trend of false negatives can be directly inferred from the trend of true positives.

**Response:**

Thank you for your comment. We have reorganized this section and thoroughly revised. As recommended, we now present some of the data using ranges instead of listing individual values, and we have highlighted the best results in bold and the worst results with underlines for clarity in the tables. Furthermore, based on your additional recommendations, we have revised our analysis to include all SVs larger than 50 bp. The manuscript has been thoroughly updated. Again, we sincerely thank you for your valuable comments.

***Page 15:***

**Results**

**The individual performance of each algorithm**

......We considered SVs if they were located within a range of +/-500 base pairs of the 'POS' (start position) and were of the same SV type as the truth set SVs. Fig 3 and Table 2 present the detailed performance of each single algorithm. *In HG002, for DELs, DRAGEN performed the best, identifying 3,425 true positives (TPs), followed by Manta, DELLY, GRIDSS, and SvABA, with LUMPY identifying the fewest. As for total error (FP + FN), DRAGEN and Manta also had the lowest error rates, while LUMPY had the highest. Similar trends were observed across other HGSVC2 datasets (Fig 3B and Table 2).*

*For INSs, DRAGEN and Manta again led in TP counts, while DELLY and GRIDSS had lower FPs but unacceptably high false negatives (FNs) across all datasets. (Fig 3B and Table 2).*......

***Page 19:***

**Results**

**Systematic identification of the performances of combination strategies**

......*For DELs in HG002, DRAGEN had the highest TP count (3,425), followed by V-union, III-union, V-multiple agreement, and the lowest for III-multiple agreement (2,554). The FP count was lowest for III-multiple agreement (533) and highest for V-union (2,199). Similar results were observed in the HGSVC2 datasets. Regarding total errors (FP + FN), five-algorithm strategies generally produced more errors than three-algorithm strategies. For INSs, DRAGEN had the highest TP counts, while multiple agreement had the highest total errors. Overall, combination strategies still struggled with a high FN rate (Fig 5B and Table 3).*......

13. Line 378. The first sentence seems to need citing a reference.

**Response:**

We appreciate the reviewer's attention to detail. We have added references to support the statement.

***Page 17:***

**Results**

**Systematic identification of the performances of combination strategies**

Due to the overall unsatisfactory results with single algorithms, we turned to a strategy commonly *employed in previous studies [7, 27, 35]*—selecting SVs commonly called by multiple algorithms to enhance the precision of the identified SVs. In our pursuit of improved SV calling accuracy, we conducted a thorough assessment of accuracy, precision, and F1 scores across various combination strategies, specifically exploring “multiple agreement” and union strategies involving three or five algorithms.

14. Line 387. It is worth to actually cite the GIAB study in this sentence.

**Response:**

We appreciate the reviewer's attention to detail. We have added references to support the statement.

***Page 18:***

**Results**

**Systematic identification of the performances of combination strategies**

For our three-algorithm combination strategies, we selected Manta, DELLY, and GRIDSS, showcasing superior performance among all tools. Meanwhile, in the case of five-algorithm combination strategies, we considered all tools except the commercial DRAGEN. Each individual SV was annotated by the name of its SV detection caller in the 'INFO' field. *Based on the original study of the GIAB benchmark set [1],* the process to form the SV benchmark includes the criterion: “Filter Complex: If two or more supported variants ≥ 50 bp were within 1000 bp of each other, they were excluded because they are potentially complex or inaccurate.” Therefore, we referenced this threshold of 1000 bp and set up a +/- 500 bp threshold for our analysis. Variants from different SV callers were grouped as neighboring SVs if they overlapped within the range of +/- 500 bp of 'POS' (start position) and shared the same 'SVTYPE.' The “multiple agreement” strategy categorizes variants as 'true-called' only if all members of the neighboring SV are detected by at least two algorithms. In contrast, the union strategy deems a variant as 'true-called' if at least one member of the neighboring SV is detected by at least one algorithm.

15. Line 406. “Close” is misspelled

**Response:**

Thank you for pointing that out. We apologize for the misspelling. The word " Close " has been corrected in the revised text. Based on your additional recommendations, we have revised our analysis to include all SVs larger than 50 bp. The results have been thoroughly updated. Again, we sincerely thank you for your valuable comments.

***Page 18:***

**Results**

**Systematic identification of the performances of combination strategies**

We evaluated the distance distribution of variant positions in neighboring SVs across different combination strategies (S1 Fig). The counts of neighboring SVs and the number of contributing member callers across different combination strategies are presented in S6 Table. All variants within a neighboring SV group were within 0 to 500 bp, as specified by our settings for combining structural variants. *The median distance of neighboring SVs across sample sets was 4.0–5.0 bp for III-multiple agreement, 7.0–10.0 bp for V-multiple agreement, and 0 bp for both III-union and V-union. The interquartile range (IQR) for neighboring SV distances was 7.0 bp for III-multiple agreement, 17.0–23.0 bp for V-multiple agreement, 1.0–2.0 bp for III-union, and 7.0–10.0 bp for V-union, indicating that the majority of neighboring SV distances were very close.*

***S1 Fig.***

**S1 Fig. Distance of neighboring SVs in combination strategies.** The distribution of distances between start positions in neighboring SVs across different combination strategies. IQR: Interquartile Range.

16. Line 432 – 434. Again, given that the gold standard is a fixed dataset, the trend of false negatives can be directly inferred from the trend of true positives.

**Response:**

Thank you for your comment. We have reorganized this section and thoroughly revised. As recommended, we now present some of the data using ranges instead of listing individual values, and we have highlighted the best results in bold and the worst results with underlines for clarity in the tables. Furthermore, based on your additional recommendations, we have revised our analysis to include all SVs larger than 50 bp. The manuscript has been thoroughly updated. Again, we sincerely thank you for your valuable comments.

***Page 18:***

**Results**

**Systematic identification of the performances of combination strategies**

For our three-algorithm combination strategies, we selected Manta, DELLY, and GRIDSS, showcasing superior performance among all tools. Meanwhile, in the case of five-algorithm combination strategies, we considered all tools except the commercial DRAGEN. Each individual SV was annotated by the name of its SV detection caller in the 'INFO' field. *Based on the original study of the GIAB benchmark set [1],* the process to form the SV benchmark includes the criterion: “Filter Complex: If two or more supported variants ≥ 50 bp were within 1000 bp of each other, they were excluded because they are potentially complex or inaccurate.” Therefore, we referenced this threshold of 1000 bp and set up a +/- 500 bp threshold for our analysis. Variants from different SV callers were grouped as neighboring SVs if they overlapped within the range of +/- 500 bp of 'POS' (start position) and shared the same 'SVTYPE.' The “multiple agreement” strategy categorizes variants as 'true-called' only if all members of the neighboring SV are detected by at least two algorithms. In contrast, the union strategy deems a variant as 'true-called' if at least one member of the neighboring SV is detected by at least one algorithm.

17. Line 451: Worst than what?

**Response:**

We appreciate the reviewer’s attention to detail and apologize for the unclear expression. The sentence has been revised for clarity. As for your additional recommendations, we have revised our analysis to include all SVs larger than 50 bp. This section has also been thoroughly updated. Again, we sincerely thank you for your valuable comments.

***Page 19:***

**Results**

**Systematic identification of the performances of combination strategies**

*For DELs in HG002, DRAGEN had the highest TP count (3,425), followed by V-union, III-union, V-multiple agreement, and the lowest for III-multiple agreement (2,554). The FP count was lowest for III-multiple agreement (533) and highest for V-union (2,199). Similar results were observed in the HGSVC2 datasets. Regarding total errors (FP + FN), five-algorithm strategies generally produced more errors than three-algorithm strategies. For INSs, DRAGEN had the highest TP counts, while multiple agreement had the highest total errors. Overall, combination strategies still struggled with a high FN rate (Fig 5B and Table 3).*

*For each combination strategy, we calculated recall, precision, and the F1 score. As shown in Fig 5C and Table 3, for DELs across sample sets, DRAGEN achieved the highest F1 score (0.64–0.68), followed by III-union and V-union, with III-multiple and V-multiple agreement scoring lower. DRAGEN had the highest recall, closely followed by the union groups (0.50–0.62), while multiple agreement groups had lower recall (0.33–0.48). In contrast, precision was higher in the multiple agreement groups (0.74–0.93) and lower in the union groups (0.61–0.76).*

*For INSs, the performance of combination strategies was also disappointing across all benchmark sets (Fig 5 and Table 3). DRAGEN achieved the highest F1 score (0.45–0.55), followed by the union groups (0.28–0.31), with the multiple agreement groups demonstrated the worst performance (0.02–0.04. Increasing the number of tools from three to five did not improve performance, regardless of whether the multiple agreement or union strategies were used.*

18. Line 478: The paper actually compares the insertions from Manta with those reported by DRAGEN. Please rephrase.

**Response:**

We appreciate the reviewer’s comment. We have revised the text in this section accordingly. Additionally, in response to another reviewer’s recommendation, the ‘Discussion' section has been thoroughly reorganized.

***Page 22:***

**Results**

**Systematic identification of the performances of combination strategies**

To determine the overall performance rank of each single caller and combination strategy in detecting DELs and INSs. we calculated the combined precision score (cPr), combined recall score (cRc), and combined F1 score (cF1). The values for all four datasets were integrated and calculated using a micro average (Table 4). Fig 6 also presents the performance across all callers based on the integrated values from all sample sets.

*For DELs, the union strategy outperformed most single algorithms (except Manta) and multiple agreement combinations in terms of F1, achieving similar F1 scores to the commercial software DRAGEN (Fig 6 and Table 4). However, while multiple agreement strategies exhibited superior precision compared to union combinations and DRAGEN, their recall was unsatisfactory.*

*For INSs, the overall performance of all single callers and combination strategies was poor, with F1 scores ranging from 0.01 to 0.30, compared to DRAGEN (0.53). In terms of precision, all single callers and combination strategies performed better than DRAGEN; however, their recall was inadequate. Among all non-commercial software, Manta had the highest F1 score (0.30), second only to DRAGEN. Among the combination strategies, the union strategy achieved an F1 score comparable to Manta. These callers and combinations exhibited moderate to high precision in detecting INSs, but their overall performance was poor mainly due to the low recall rate (Fig 6 and Table 4).*

*Overall, increasing the number of tools from three to five did not improve performance, regardless of whether the multiple agreement or union strategies were used. However, using combination strategies enhanced performance compared to using most single callers alone.*

19. Line 490: Why the similarities are “striking”.

**Response:**

We appreciate the reviewer’s comment and apologize for the unclear expression. We have revised the text in this section accordingly. Additionally, in response to another reviewer’s recommendation, the ‘Discussion’ section has been thoroughly reorganized.

***Page 24:***

**Discussion**

SVs can have a substantial phenotypic impact, disrupting gene function, regulation, or modifying gene dosage. Their significance in medicine and molecular biology has been underscored by recent studies [36]. The role of SVs in the pathogenicity of genetic diseases is increasingly recognized, and there is a possibility that screening for SVs may become routine in clinical settings in the future.

According to our study, relying on a single algorithm for SV detection may pose the risk of limited SV size and type detection, along with low F1 score. While our multiple agreement strategy, which focuses on consensus among algorithms, tends to yield higher precision at the cost of lower recall, employing a union combination *strategy ~~covers a broader range of SV sizes and types more effectively. This approach~~* enhanced recall and F1 scores for DELs compared to both single algorithms and the multiple agreement approach. *However, most callers show poor F1 scores in INS detection due to low recall rates.  Specifically, DELLY (0.02) and GRIDSS (0.01) exhibited very poor F1 scores, while Manta (0.30) were the top performers among all non-commercial tools. The union strategy, which includes Manta, consequently achieved a comparable F1 score for INS detection, as illustrated in (Fig. 6).* This underscores the importance of choosing well-performing callers tailored to specific types and sizes of SVs for different clinical scenarios.

Recently, Ramesh Rajaby and colleagues developed an INS caller, INSurVeyor [37], which improves the sensitivity of INS calling from short-read sequencing data, performing even better than Manta. Building on the foundation of our study, we believe that by integrating algorithms that perform well for specific SV types, such as INS detection, and using a union strategy, excellent performance can be achieved tailored to various detection goals. Moreover, recently, Guiwu Zhuang et al. [38] established an expert-reviewed tumor-specific clinically relevant SVs call set, evaluating several algorithms for SV detection, and developed a random-forest-based decision model to improve the precision. Building on these research foundations, it is worth exploring the potential of integrating the union combination strategy with an machine learning (ML) based decision model in the future. For practical clinical applications, further investigation into the performance using real clinical sample sets is necessary…….

*As for the concern of FP variants that may could be falsely related to a phenotype. We were particularly interested in FPs that are consistently detected by multiple callers. We analyzed the FPs called by at least two algorithms in the HG002 dataset and compared them with DRAGEN. We differentiated FPs identified only by the multi-agreement strategy (M), only by DRAGEN (D), and those identified by both (Both), the FP SV size displaying the median and interquartile range were shown as S4_figure. We found that the majority of FPs are smaller than 10^3 base pairs. For DEL FPs, the M group had a median SV length of 326 bp, while the D group had a median of 120 bp. As for INSs, due to the poor recall of most tools, the number of FPs was quite low, with D group INS FPs having a median SV length of 81 bp. Considering that most short reads range from 300 to 500 bp in length, we believe they should be capable of accurately sequencing variants of this small size. Each tool has its own underlying principles and parameters for detecting SVs, as outlined in their respective publications, many employ hard filters—such as thresholds for variant quality, read depth—to reduce FPs. However, care is needed to balance filtering to avoid discarding true positives.* In clinical practice, post-processing tasks like filtering target genes using a virtual panel, along with additional validation through the Integrative Genomics Viewer (IGV) or methods such as PCR, are commonly used. Therefore, weighing these considerations, we believe that achieving a high recall rate to minimize FN enhance the utility of these findings in clinical settings.

*Several studies also shown that the occurrence of FPs in short-read sequencing for SV identification can be influenced by various factors, affecting overall precision. For example, Daniel L. Cameron et al. assessed the impact of sequence context and event size on the precision of 10 SV callers, evaluating these tools using simulation data and well-referenced cell lines. Their study included several tools that we also selected, such as Manta, DELLY, GRIDSS, and LUMPY. They identified several major factors impact the precision: 1) The proximity of single-nucleotide variants (SNVs) or indels near the SV breakpoints. When multiple flanking variants are present, false positive rates increase. 2) SVs located in low-complexity and simple tandem repeat regions are prone to higher false positive rates. SVs within LTR, LINEs and SINEs regions also show reduced precision, leading to more false positives. 3) The size of the event also plays a role; they indicated SVs smaller than 100 bp are particularly challenging, whereas deletions between 300 to 500 bp tend to achieve higher precision.*

Finally, when addressing SV calling, the role of long-read sequencing cannot be overlooked. Long-read sequencing offers remarkable advantages as it can span repetitive or other problematic regions. For applications that require identifying very long or complex SVs, performing a localized long-read de novo assembly is more effective than conventional short-read mapping approaches. *Multiple studies have demonstrated that long-read sequencing technologies offer superior accuracy in SV detection compared to short-read approaches. Benchmarking results, as shown in recent studies [31, 39, 40] , consistently highlight the advantages of long reads in terms of sensitivity and specificity, regardless of the specific tools used. Moreover, the recent advancements in long-read technologies and the ongoing reductions in sequencing costs—led by both Oxford Nanopore and PacBio—have made long-read sequencing increasingly accessible and widespread. Integrating short-read and long-read sequencing provide opportunities for developing strategies tailored to diverse research and clinical needs.*

Although our study only focused on the DELs and INSs— which are the majority but relatively simple SV types in the human genome— methods failing to detect DELs and INSs might also be less effective at identifying more complex SV types. Currently, our study evaluated the ability of various methods and combination strategies to detect DELs and INSs, providing a valuable and optimistic perspective on overall SV detection performance.

20. Line 492: Significantly more intricate than what? Please provide an statistical test and p-value to assess significance.

**Response:**

Thank you for your comment. We apologize for any confusion. The term "significant" in this context was not intended to imply statistical significance. We have removed all instances of "significant" that were not used in a statistical context to avoid any misunderstanding. Additionally, in response to another reviewer’s recommendation, the ‘Discussion’ section has been thoroughly reorganized. Again, we sincerely thank you for your valuable comments.

***Page 24:***

**Discussion**

SVs can have a substantial phenotypic impact, disrupting gene function, regulation, or modifying gene dosage. Their significance in medicine and molecular biology has been underscored by recent studies [36]. The role of SVs in the pathogenicity of genetic diseases is increasingly recognized, and there is a possibility that screening for SVs may become routine in clinical settings in the future.

According to our study, relying on a single algorithm for SV detection may pose the risk of limited SV size and type detection, along with low F1 score. While our multiple agreement strategy, which focuses on consensus among algorithms, tends to yield higher precision at the cost of lower recall, employing a union combination *strategy ~~covers a broader range of SV sizes and types more effectively. This approach~~* enhanced recall and F1 scores for DELs compared to both single algorithms and the multiple agreement approach. *However, most callers show poor F1 scores in INS detection due to low recall rates.  Specifically, DELLY (0.02) and GRIDSS (0.01) exhibited very poor F1 scores, while Manta (0.30) were the top performers among all non-commercial tools. The union strategy, which includes Manta, consequently achieved a comparable F1 score for INS detection, as illustrated in (Fig. 6).* This underscores the importance of choosing well-performing callers tailored to specific types and sizes of SVs for different clinical scenarios.

Recently, Ramesh Rajaby and colleagues developed an INS caller, INSurVeyor [37], which improves the sensitivity of INS calling from short-read sequencing data, performing even better than Manta. Building on the foundation of our study, we believe that by integrating algorithms that perform well for specific SV types, such as INS detection, and using a union strategy, excellent performance can be achieved tailored to various detection goals. Moreover, recently, Guiwu Zhuang et al. [38] established an expert-reviewed tumor-specific clinically relevant SVs call set, evaluating several algorithms for SV detection, and developed a random-forest-based decision model to improve the precision. Building on these research foundations, it is worth exploring the potential of integrating the union combination strategy with an machine learning (ML) based decision model in the future. For practical clinical applications, further investigation into the performance using real clinical sample sets is necessary…….

*As for the concern of FP variants that may could be falsely related to a phenotype. We were particularly interested in FPs that are consistently detected by multiple callers. We analyzed the FPs called by at least two algorithms in the HG002 dataset and compared them with DRAGEN. We differentiated FPs identified only by the multi-agreement strategy (M), only by DRAGEN (D), and those identified by both (Both), the FP SV size displaying the median and interquartile range were shown as S4_figure. We found that the majority of FPs are smaller than 10^3 base pairs. For DEL FPs, the M group had a median SV length of 326 bp, while the D group had a median of 120 bp. As for INSs, due to the poor recall of most tools, the number of FPs was quite low, with D group INS FPs having a median SV length of 81 bp. Considering that most short reads range from 300 to 500 bp in length, we believe they should be capable of accurately sequencing variants of this small size. Each tool has its own underlying principles and parameters for detecting SVs, as outlined in their respective publications, many employ hard filters—such as thresholds for variant quality, read depth—to reduce FPs. However, care is needed to balance filtering to avoid discarding true positives.* In clinical practice, post-processing tasks like filtering target genes using a virtual panel, along with additional validation through the Integrative Genomics Viewer (IGV) or methods such as PCR, are commonly used. Therefore, weighing these considerations, we believe that achieving a high recall rate to minimize FN enhance the utility of these findings in clinical settings.

*Several studies also shown that the occurrence of FPs in short-read sequencing for SV identification can be influenced by various factors, affecting overall precision. For example, Daniel L. Cameron et al. assessed the impact of sequence context and event size on the precision of 10 SV callers, evaluating these tools using simulation data and well-referenced cell lines. Their study included several tools that we also selected, such as Manta, DELLY, GRIDSS, and LUMPY. They identified several major factors impact the precision: 1) The proximity of single-nucleotide variants (SNVs) or indels near the SV breakpoints. When multiple flanking variants are present, false positive rates increase. 2) SVs located in low-complexity and simple tandem repeat regions are prone to higher false positive rates. SVs within LTR, LINEs and SINEs regions also show reduced precision, leading to more false positives. 3) The size of the event also plays a role; they indicated SVs smaller than 100 bp are particularly challenging, whereas deletions between 300 to 500 bp tend to achieve higher precision.*

Finally, when addressing SV calling, the role of long-read sequencing cannot be overlooked. Long-read sequencing offers remarkable advantages as it can span repetitive or other problematic regions. For applications that require identifying very long or complex SVs, performing a localized long-read de novo assembly is more effective than conventional short-read mapping approaches. *Multiple studies have demonstrated that long-read sequencing technologies offer superior accuracy in SV detection compared to short-read approaches. Benchmarking results, as shown in recent studies [31, 39, 40] , consistently highlight the advantages of long reads in terms of sensitivity and specificity, regardless of the specific tools used. Moreover, the recent advancements in long-read technologies and the ongoing reductions in sequencing costs—led by both Oxford Nanopore and PacBio—have made long-read sequencing increasingly accessible and widespread. Integrating short-read and long-read sequencing provide opportunities for developing strategies tailored to diverse research and clinical needs.*

Although our study only focused on the DELs and INSs— which are the majority but relatively simple SV types in the human genome— methods failing to detect DELs and INSs might also be less effective at identifying more complex SV types. Currently, our study evaluated the ability of various methods and combination strategies to detect DELs and INSs, providing a valuable and optimistic perspective on overall SV detection performance.

21. Line 533. The sentence is vague. Why the union method has “comprehensive coverage”? Moreover, the sentence is contradictory with the results and even with the previous sentence. The previous sentence says that the performance of individual callers is “unsatisfactory due to a poor recall rate”, and then this sentence says that the individual callers have “strong performance”.

**Response:**

Thank you for your insightful comment. We apologize for the confusion caused by the vague and contradictory statements. Based on your additional recommendations, and another reviewer’s suggestion, the ‘Discussion’ section has been thoroughly revised. Again, we sincerely thank you for your valuable comments.

***Page 24:***

**Discussion**

......According to our study, relying on a single algorithm for SV detection may pose the risk of limited SV size and type detection, along with low F1 score. While our multiple agreement strategy, which focuses on consensus among algorithms, tends to yield higher precision at the cost of lower recall, employing a union combination *strategy ~~covers a broader range of SV sizes and types more effectively. This approach~~* enhanced recall and F1 scores for DELs compared to both single algorithms and the multiple agreement approach. *However, most callers show poor F1 scores in INS detection due to low recall rates. Specifically, DELLY (0.02) and GRIDSS (0.01) exhibited very poor F1 scores, while Manta (0.30) were the top performers among all non-commercial tools. The union strategy, which includes Manta, consequently achieved a comparable F1 score for INS detection, as illustrated in (Fig. 6).* This underscores the importance of choosing well-performing callers tailored to specific types and sizes of SVs for different clinical scenarios......

**Reviewer #3**

*Comments to the Author*

The author evaluated six classic SV detection tools on second-generation sequencing data and explored the impact of merging strategies on improving SV detection performance. Below are some suggestions regarding this work:

*Major comments*

1. The author's combination experiment results indicate that the combination strategies indeed improved performance by increasing either recall or precision. However, while this conclusion is evident, the results of the two merging strategies still did not surpass the performance of the single tool, DRAGEN. To further enhance SV detection performance, the author should explore the feasibility of combination strategies based on DRAGEN’s SV detection results.

**Response:**

We sincerely appreciate the reviewer’s detailed review and valuable comments. Further exploration of strategies to enhance SV detection performance is of great interest to us.

In this study, we demonstrated that combination strategies improved performance compared to most single callers, particularly through the union strategy, which produced F1 scores for DELs comparable to DRAGEN and improved INS detection. While we did not surpass DRAGEN, our goal was to showcase the utility of free tools in achieving comparable performance.

When designing the combination strategy, we considered the clinical use of post-processing tasks, such as filtering target genes through a virtual panel and validation using IGV or PCR. We aimed to improve overall performance while preserving recall, leading to the development of the III or V multiple-agreement and union combination strategies. Although these strategies did not outperform DRAGEN, they did help us uncover key insights. For instance, in the union strategy with Manta, we achieved a comparable F1 score for INS detection. Additionally, we found that increasing the number of tools from three to five did not improve performance across combination strategies. Therefore, selecting high-performing callers for specific SV types remains critical.

Based on the study’s conclusions, we believe that to further improve precision without compromising recall, carefully selecting and integrating higher-performing callers could be a viable approach to surpassing DRAGEN. Recently, Ramesh Rajaby and colleagues developed INSurVeyor, an INS caller that improves sensitivity for INS detection from short-read sequencing, outperforming Manta. This suggests opportunities to integrate better-performing algorithms, such as using INSurVeyor in union strategies for enhanced performance. Similarly, Guiwu Zhuang et al. created a tumor-specific SV call set using machine learning to improve precision. Building on our study, integrating ML-based decision models with a union strategy also holds potential for future improvements. We sincerely appreciate the reviewer’s insightful comments.

*References:*

- Rajaby R, Liu D-X, Au CH, Cheung Y-T, Lau AYT, Yang Q-Y, Sung W-K. INSurVeyor: improving insertion calling from short read sequencing data. Nature Communications. 2023;14(1):3243. doi: 10.1038/s41467-023-38870-2.
- Zhuang G, Zhang X, Du W, Xu L, Ma J, Luo H, et al. A benchmarking framework for the accurate and cost-effective detection of clinically-relevant structural variants for cancer target identification and diagnosis. Journal of Translational Medicine. 2024;22(1):65. doi: 10.1186/s12967-024-04865-w.

2. According to the methods described, the author’s validation approach for the combination strategy involves retaining the merged SVs from each VCF file and then calculating the overall TP, FP, and FN values. A more direct approach would be to merge the results into a single VCF file and then calculate the F1 score. The author might consider comparing the outcomes of these two validation methods.

**Response:**

We sincerely appreciate the reviewer’s detailed review and the valuable comments provided. We understand that the method suggested by the reviewer is a viable one for testing.

In our study, we grouped neighboring SVs whose start positions were within +/- 500 bases of each other and shared the same SV type. This was based on the original study of the Genome in a Bottle (GIAB) benchmark set, the process to form the SV benchmark includes the criterion: “Filter Complex: If two or more supported variants ≥50 bp were within 1000 bp of each other, they were excluded because they are potentially complex or inaccurate.” This implies that, within a range of 1000 bp, usually only one SV is detected unless it is complex or inaccurate. Therefore, we referenced this threshold of 1000 bp and set up a ±500 base pairs threshold for our analysis.

Following the reviewer's suggestion, we directly merged results into a single VCF file and then calculating the F1 score. Without grouping neighboring SVs, deduplication was performed only for identical records from different callers, potentially resulting in double-counting if two records were close but not merged. We then calculated the F1, recall, and precision for this approach (As shown in S5 figure, the darker color represents results from the ‘directly merge’ method.) For DEL, the multiple agreement strategies of ‘directly merge method’ showed improved precision but lower recall and F1 due to the strict matching of SVs. Conversely, in the union approach, precision decreased while recall and F1 increased. However, it’s important to note that due to the inability to avoid possible double counting, the recall exceeds 1 in some cases. For INS, due to the initially poor detection rates of individual callers, there was no significant difference between the original and ‘directly merge method.’

We understand the concerns that the neighboring SV method might cause some SVs to be lost during the merging process. To address this, we analyzed the distances between start positions of neighboring SVs across different combination strategies and found that most grouped SVs were within 25 bp of each other, indicating close proximity (S1_Fig.). We also performed statistical analysis on the average number of neighboring SVs per group. For instance, in the group using five callers, there should be at most five neighboring SVs per group. Identifying more than five suggests the presence of additional, smaller individual SVs. Our data shows that, in the group using three SV callers, the majority had fewer than three member variants, and in the group using five SV callers, the majority had fewer than five member variants, as detailed in S6 Table. This suggests that our +/- 500 bp merging strategy rarely grouped distinct SVs inaccurately. Therefore, we consider that using the neighboring SV method to correct for double counting more accurately reflects the true performance of the detection algorithms.

Additionally, since short reads typically range from 300 to 500 bp, we believe that SVs within this size range can be accurately sequenced. Additionally, since short reads typically range from 300 to 500 bp, we believe that SVs within this size range can be accurately sequenced. In practice, tools like IGV or PCR can help clarify cases where two SVs are within 1000 bp of each other, making it feasible to distinguish between them in real-world settings. Again, we sincerely appreciate the reviewer’s insightful comments.

***S5 Fig.***

***S5 Fig.*** ***Combination performance of each single caller and combination strategy in detecting DELs and INSs using the ‘neighboring SV method’ and ‘directly merge method’.*** *The maximum, minimum, and macro averages for recall, precision, and F1 score are calculated across all sample sets. Darker colors represent results from the 'directly merge method’. The 'neighboring SV method' is explained in the 'Combination strategies of multiple algorithms' section of the Methods, while the 'directly merge method' involves merging all caller results into one VCF file before calculating the F1 score.*

3. In the results section, the extensive listing of data might obscure the main conclusions. It is recommended that the author focus on summarizing the key conclusions, present comparative data in tables, and highlight the best results in bold.

Response:

We appreciate the reviewer's suggestion. As recommended, we now present some of the data using ranges instead of listing individual values, and we have highlighted the best results in bold and the worst results with underlines for clarity in the tables. Furthermore, based on the other reviewer’s recommendations, we have revised our analysis to include all SVs larger than 50 bp. The manuscript has been thoroughly updated. Again, we sincerely thank you for your valuable comments.

***Page 15:***

**Results**

**The individual performance of each algorithm**

......We considered SVs if they were located within a range of +/-500 base pairs of the 'POS' (start position) and were of the same SV type as the truth set SVs. Fig 3 and Table 2 present the detailed performance of each single algorithm. *In HG002, for DELs, DRAGEN performed the best, identifying 3,425 true positives (TPs), followed by Manta, DELLY, GRIDSS, and SvABA, with LUMPY identifying the fewest. As for total error (FP + FN), DRAGEN and Manta also had the lowest error rates, while LUMPY had the highest. Similar trends were observed across other HGSVC2 datasets (Fig 3B and Table 2).*

*For INSs, DRAGEN and Manta again led in TP counts, while DELLY and GRIDSS had lower FPs but unacceptably high false negatives (FNs) across all datasets. (Fig 3B and Table 2).*......

***Page 17:***

**Results**

**The individual performance of each algorithm**

......For each distinct algorithm, we computed the recall, precision, and F1 score. *As shown in Fig 3C and Table 2, for DELs across all benchmark sets, DRAGEN achieved the highest F1 scores (0.64–0.68), followed closely by Manta, DELLY, and GRIDSS. LUMPY scored the lowest F1 in HG002 (0.19), while SvABA had mid-range scores in HG002 but lower in other datasets. Among all non-commercial software, Manta had the highest recall (0.43–0.55%), with GRIDSS showing lower recall but higher precision (0.86–0.95).*

*We also conducted a performance analysis of INSs, but most single algorithms performed disappointingly. Across all datasets, DRAGEN achieved the highest F1 scores (0.45–0.55), followed by Manta. DELLY (0.02–0.04) and GRIDSS (0.01–0.02) had very unsatisfactory F1 scores. Among non-commercial software, Manta had the highest recall (0.16–0.19) but only moderate precision (0.77–0.96). In contrast, GRIDSS and DELLY showed better precision (0.91–0.99) but poor recall (0.00–0.02%). Overall, low recall impacted the performance of these tools (Fig 3C and Table 2).......*

***Page 19:***

**Results**

**Systematic identification of the performances of combination strategies**

......*For DELs in HG002, DRAGEN had the highest TP count (3,425), followed by V-union, III-union, V-multiple agreement, and the lowest for III-multiple agreement (2,554). The FP count was lowest for III-multiple agreement (533) and highest for V-union (2,199). Similar results were observed in the HGSVC2 datasets. Regarding total errors (FP + FN), five-algorithm strategies generally produced more errors than three-algorithm strategies. For INSs, DRAGEN had the highest TP counts, while multiple agreement had the highest total errors. Overall, combination strategies still struggled with a high FN rate (Fig 5B and Table 3).*

*For each combination strategy, we calculated recall, precision, and the F1 score. As shown in Fig 5C and Table 3, for DELs across sample sets, DRAGEN achieved the highest F1 score (0.64–0.68), followed by III-union and V-union, with III-multiple and V-multiple agreement scoring lower. DRAGEN had the highest recall, closely followed by the union groups (0.50–0.62), while multiple agreement groups had lower recall (0.33–0.48). In contrast, precision was higher in the multiple agreement groups (0.74–0.93) and lower in the union groups (0.61–0.76).*

*For INSs, the performance of combination strategies was also disappointing across all benchmark sets (Fig 5 and Table 3). DRAGEN achieved the highest F1 score (0.45–0.55), followed by the union groups (0.28–0.31), with the multiple agreement groups demonstrated the worst performance (0.02–0.04. Increasing the number of tools from three to five did not improve performance, regardless of whether the multiple agreement or union strategies were used*.

4. The discussion section devotes significant space to background and motivation, which would be better emphasized in the introduction. It is advisable to reposition this content accordingly.

Response:

We sincerely appreciate the reviewer’s recommendation. In response to reviewer’s insightful suggestion, we have moved the relevant content from the ‘Discussion’ section to the ‘Introduction’. Both sections have been reorganized accordingly. Once again, thank you for your thoughtful advice.

***Page 3:***

**Introduction**

Genomic structural variants (SVs) are substantial alterations in the genome, varying in size from 50 to mega base pairs (bp) ~~base pairs~~. These variants include unbalanced gains and losses of DNA segments as well as balanced rearrangements. SVs are commonly classified into five types: deletions (DELs), insertions (INSs), duplications (DUPs), inversions (INVs), and complex translocations (CTXs) [1]. Owing to their large size, SVs account for a greater proportion of heritable sequence differences between individuals compared to single nucleotide variants (SNVs), with SVs constituting 0.5–1% of the genome and SNVs approximately 0.1% [2].

SVs contribute to genetic diversity and evolution at both individual and population levels. They exert a more profound impact on gene functions and phenotypic changes compared to SNVs and short insertion-deletions (indels) and are associated with various human diseases [3]. Polymorphic SVs are major contributors to common traits, including widespread diseases, while rare germline SVs are responsible for many rare genomic disorders [4].

With the advent of next-generation sequencing (NGS), the detection of SNVs and small indels (<50 bp) has become highly accurate, with an error rate of less than 0.1%. Despite substantial advancements in numerous SV detection methods, their effectiveness in short-read sequences remains limited [5]. Sequencing-based methods employ several approaches to derive information about SVs from short-read sequencing data: read-pair (RP), read-depth (RD), split-read (SR), and assembly (AS) approaches [6-8]. RP uses the discordant alignment features of paired-end reads that encompass or overlap an SV. RD utilizes the depth features of paired-end reads that overlap an SV, indicating changes in read depth due to the SV. SR uses split (soft-clipped) alignment features of single-end or paired-end reads that span a breakpoint (BP) of an SV. AS detects SVs by aligning contigs, assembled from the entire or unmapped sequencing reads, to the reference sequence.

Despite greatly growth and improvement in numerous SV detection methods over the years, their performance in short-read sequences remains constrained. Sequencing-based approaches exhibit a high rate of SV miscalling, irrespective of the strategy employed. To address the limitations of short-read sequencing, recent efforts have utilized long reads generated through single-molecule sequencing technology for SV detection in human samples, employing assembly (AS) and/or split-read (SR) approaches.[9-11]

*Recently, long-read technologies such as Oxford Nanopore Technologies (ONT) and PacBio HiFi sequencing have made remarkable advancements, becoming more accessible and offering higher accuracy. These improvements help overcome many of the limitations associated with short-read sequencing [12].* However, despite these advancements and cost reductions making long-read sequencing more feasible, substantial challenges remain. These include not only higher costs, scalability issues, and stringent requirements for sample quantity and quality [13, 14], but also important considerations for the vast amounts of existing short-read data. For instance, many biobanks contain extensive archives of short-read data, which continue to have a high demand for reanalysis. The UK Biobank, for example, has sequenced the whole genomes of 150,119 participants using Illumina NovaSeq sequencing [15]. Similarly, while the All of Us (AoU) project has re-sequenced some samples with long-read techniques, it has already released over 100,000 datasets from Illumina whole genome sequencing (WGS) [16], highlighting the ongoing reliance on short-read approaches.

*Today, there has been substantial progress in short-read-based general-purpose SV detection, with numerous* SV detection algorithms being published. Many projects use popular SV detection algorithms showed a relatively high accuracy [17-19]. Although sequencing-based methods theoretically detect any type of SV, no single algorithm can accurately and sensitively detect all types and sizes [20-22]. *Several studies have highlighted the individual performance of various callers. For example, Varuni Sarwal et al. [23] evaluated the performance of SV detection tools on mouse and human WGS data, focusing on DELs detection using a comprehensive set confirmed by polymerase chain reaction (PCR) and the GIAB benchmark set.*

*Evaluations of combination strategies, however, remain relatively scarce. Some studies evaluated the performance of combining the consensus of individual tools. Daniel L. Cameron et al. [24] assessed 10 SV callers individually and in possible m-of-n ensembles using well-referenced cell lines such as NA12878, HG002, CHM1, and CHM13. They found that while simple ensemble calling sometimes improves the performance of the best individual callers, it is highly sensitive to the specific callers chosen for the ensemble. Similarly, Kosugi et al. [7] analyzed the performance of 69 SV detection algorithms and the effectiveness of combining pairs of these algorithms. They observed that when pairs of SV detection algorithms were analyzed, SVs commonly identified by multiple algorithms exhibited higher precision but lower recall than those identified by a single algorithm.*

In a benchmarking study assessing 46 SV callers published between 2009 and 2017 [24], DELLY [17], LUMPY [18], Manta [19], and GRIDSS [25] were identified as popular tools based on their citation counts in the Web of Science database. *While SvABA [26] was not included due to its 2018 publication date, it has shown robust performance in detecting both germline and somatic SVs in other assessments [27] .*

*Building on these, our study reviews six state-of-the-art SV detection algorithms, including five previously mentioned tools and a commercial caller DRAGEN, to establish effective SV detection strategies. DELLY combines read RP and SR methods for SV detection, integrating paired-end short reads data from different insert sizes. LUMPY uses a probabilistic representation of SV breakpoints, leveraging RP and SR analyses and clustering overlapping breakpoints to identify SVs. Manta employs a two-phase workflow for high parallelization, building a graph of break-end associations and processing it for variant hypothesis generation, assembly, scoring, and VCF reporting. GRIDSS detects SVs of any size using AS, RP, and SR methods, generating break-end contigs and using a probabilistic model to infer SVs. SvABA operates within local 25k base pairs assembly windows with 2k base pairs overlaps, efficiently detecting SVs from short-read sequencing data with low resource requirements.*

We analyze the SV count, type, and length detected by each algorithm and conduct a comprehensive evaluation of their performance. *Additionally, we proposed combination strategies that involved multiple agreement strategies, which requires the consensus of more than two tools, as well as union strategies that integrates the outputs of all selected algorithms.* Our analysis demonstrates the union approach achieved higher recall rates compared to single algorithms and attained similar recall and F1 scores to the commercial software DRAGEN.

***Page 24:***

**Discussion**

SVs can have a substantial phenotypic impact, disrupting gene function, regulation, or modifying gene dosage. Their significance in medicine and molecular biology has been underscored by recent studies [36]. The role of SVs in the pathogenicity of genetic diseases is increasingly recognized, and there is a possibility that screening for SVs may become routine in clinical settings in the future.

According to our study, relying on a single algorithm for SV detection may pose the risk of limited SV size and type detection, along with low F1 score. While our multiple agreement strategy, which focuses on consensus among algorithms, tends to yield higher precision at the cost of lower recall, employing a union combination *strategy ~~covers a broader range of SV sizes and types more effectively. This approach~~* enhanced recall and F1 scores for DELs compared to both single algorithms and the multiple agreement approach. *However, most callers show poor F1 scores in INS detection due to low recall rates.  Specifically, DELLY (0.02) and GRIDSS (0.01) exhibited very poor F1 scores, while Manta (0.30) were the top performers among all non-commercial tools. The union strategy, which includes Manta, consequently achieved a comparable F1 score for INS detection, as illustrated in (Fig. 6).* This underscores the importance of choosing well-performing callers tailored to specific types and sizes of SVs for different clinical scenarios.

Recently, Ramesh Rajaby and colleagues developed an INS caller, INSurVeyor [37], which improves the sensitivity of INS calling from short-read sequencing data, performing even better than Manta. Building on the foundation of our study, we believe that by integrating algorithms that perform well for specific SV types, such as INS detection, and using a union strategy, excellent performance can be achieved tailored to various detection goals. Moreover, recently, Guiwu Zhuang et al. [38] established an expert-reviewed tumor-specific clinically relevant SVs call set, evaluating several algorithms for SV detection, and developed a random-forest-based decision model to improve the precision. Building on these research foundations, it is worth exploring the potential of integrating the union combination strategy with an machine learning (ML) based decision model in the future. For practical clinical applications, further investigation into the performance using real clinical sample sets is necessary.

*As for the concern of FP variants that may could be falsely related to a phenotype. We were particularly interested in FPs that are consistently detected by multiple callers. We analyzed the FPs called by at least two algorithms in the HG002 dataset and compared them with DRAGEN. We differentiated FPs identified only by the multi-agreement strategy (M), only by DRAGEN (D), and those identified by both (Both), the FP SV size displaying the median and interquartile range were shown as S4_figure. We found that the majority of FPs are smaller than 10^3 base pairs. For DEL FPs, the M group had a median SV length of 326 bp, while the D group had a median of 120 bp. As for INSs, due to the poor recall of most tools, the number of FPs was quite low, with D group INS FPs having a median SV length of 81 bp. Considering that most short reads range from 300 to 500 bp in length, we believe they should be capable of accurately sequencing variants of this small size. Each tool has its own underlying principles and parameters for detecting SVs, as outlined in their respective publications, many employ hard filters—such as thresholds for variant quality, read depth—to reduce FPs. However, care is needed to balance filtering to avoid discarding true positives.* In clinical practice, post-processing tasks like filtering target genes using a virtual panel, along with additional validation through the Integrative Genomics Viewer (IGV) or methods such as PCR, are commonly used. Therefore, weighing these considerations, we believe that achieving a high recall rate to minimize FN enhance the utility of these findings in clinical settings.

*Several studies also shown that the occurrence of FPs in short-read sequencing for SV identification can be influenced by various factors, affecting overall precision. For example, Daniel L. Cameron et al. assessed the impact of sequence context and event size on the precision of 10 SV callers, evaluating these tools using simulation data and well-referenced cell lines. Their study included several tools that we also selected, such as Manta, DELLY, GRIDSS, and LUMPY. They identified several major factors impact the precision: 1) The proximity of single-nucleotide variants (SNVs) or indels near the SV breakpoints. When multiple flanking variants are present, false positive rates increase. 2) SVs located in low-complexity and simple tandem repeat regions are prone to higher false positive rates. SVs within LTR, LINEs and SINEs regions also show reduced precision, leading to more false positives. 3) The size of the event also plays a role; they indicated SVs smaller than 100 bp are particularly challenging, whereas deletions between 300 to 500 bp tend to achieve higher precision.*

Finally, when addressing SV calling, the role of long-read sequencing cannot be overlooked. Long-read sequencing offers remarkable advantages as it can span repetitive or other problematic regions. For applications that require identifying very long or complex SVs, performing a localized long-read de novo assembly is more effective than conventional short-read mapping approaches. *Multiple studies have demonstrated that long-read sequencing technologies offer superior accuracy in SV detection compared to short-read approaches. Benchmarking results, as shown in recent studies [31, 39, 40] , consistently highlight the advantages of long reads in terms of sensitivity and specificity, regardless of the specific tools used. Moreover, the recent advancements in long-read technologies and the ongoing reductions in sequencing costs—led by both Oxford Nanopore and PacBio—have made long-read sequencing increasingly accessible and widespread. Integrating short-read and long-read sequencing provide opportunities for developing strategies tailored to diverse research and clinical needs.*

Although our study only focused on the DELs and INSs— which are the majority but relatively simple SV types in the human genome— methods failing to detect DELs and INSs might also be less effective at identifying more complex SV types. Currently, our study evaluated the ability of various methods and combination strategies to detect DELs and INSs, providing a valuable and optimistic perspective on overall SV detection performance.

5. The author is advised to refine the abstract for clarity and conciseness.

Response:

We sincerely appreciate the reviewer's suggestion. Following your advice, we have rewritten the ‘Abstract’ to improve clarity and conciseness.

***Page 2:***

**Abstract**

Structural variants (SVs) *have been associated with changes in gene expression, which may contribute to alterations in phenotypes and disease development.* However, the precise identification and characterization of SVs remain challenging. While long-read sequencing offers superior accuracy for SV detection, short-read sequencing remains essential due to practical and cost considerations, as well as the need to analyze existing short-read datasets. *~~To address this, Multiple methodologies targeting different SV classes, zygosities, and size ranges have been developed.~~ ~~Detecting SVs using short-read sequencing data is particularly challenging, often leading to low sensitivity and high false discovery rates.~~ Numerous algorithms for short-read SV detection exist, none are universally optimal, each having limitations for specific SV sizes and types. In this study, we evaluated the efficacy of six advanced SV detection algorithms, including the commercial software DRAGEN, using the GIAB v0.6 Tier 1 benchmark and HGSVC2 cell lines. We employed both individual and combination strategies, with systematic assessments of recall, precision, and F1 scores. Our results demonstrate that union combination approach enhanced detection capabilities, surpassing single algorithms in identifying deletions and insertions, delivered comparable recall and F1 scores to the commercial software DRAGEN. Interestingly, expanding the number of algorithms from three to five in the combination did not enhance performance, highlighting the efficiency of a well-chosen ensemble over a larger algorithmic pool.*

6. The low recall rate of DELLY and GRIDSS tools in detecting INS variants is unconvincing. The author should verify the data or provide a reasonable explanation based on the literature.

Response:

We sincerely appreciate the reviewer’s comment. Following another reviewer’s recommendation in a previous revision, we have included three additional datasets from HGSVC2 (HG00514, HG00733, NA19240), alongside HG002 from GIAB, to enhance robustness and provide a more comprehensive assessment. Across these datasets, DELLY and GRIDSS consistently exhibited low recall rates for detecting INSs, which aligns with findings from previous studies.

Joe, S. et al. compared 11 SV callers, including DELLY, Manta, and GRIDSS, in detecting INSs in the HG002 benchmark. Similarly, Cameron, D.L. et al. evaluated the sensitivity of SV callers on simulated data across various event types, including INS. Among the tools, Manta showed superior performance for INS detection, while DELLY and GRIDSS, though effective for DELs, performed inferior recall for INSs.

Recently, Ramesh Rajaby and colleagues developed an INS caller, INSurVeyor [37], which improves the sensitivity of INS calling from short-read sequencing data, performing even better than Manta. This highlights opportunities to integrate better-performing algorithms tailored to specific SV types, like using INSurVeyor in combination with union strategies for enhanced performance. Once again, we thank reviewer’s thoughtful comment.

Refernces:

- Joe, S., Park, JL., Kim, J. et al. Comparison of structural variant callers for massive whole-genome sequence data. BMC Genomics 25, 318 (2024). <https://doi.org/10.1186/s12864-024-10239-9>
- Cameron, D.L., Di Stefano, L. & Papenfuss, A.T. Comprehensive evaluation and characterisation of short read general-purpose structural variant calling software. Nat Commun 10, 3240 (2019). <https://doi.org/10.1038/s41467-019-11146-4>
- Rajaby, R., Liu, DX., Au, C.H. et al. INSurVeyor: improving insertion calling from short read sequencing data. Nat Commun 14, 3243 (2023). <https://doi.org/10.1038/s41467-023-38870-2>

7. All formulas in the manuscript should be numbered for reference and citation purposes.

Response:

Thank you for the helpful suggestion. We have numbered all formulas in the ‘Methods’ section in the revised manuscript.

***Page 8:***

***Materials and Methods***

**Evaluation of performance for single algorithm and combination strategies**

The recall, precision, and F1 score for different single algorithms and combinations of multiple algorithms, either in “multiple agreement” strategy or union strategy, were calculated. We focused on the SV types 'DEL' and 'INS' *~~within the range of 50 to 10k base pairs~~* to analyze the performance of different single algorithms and various combination strategies, because the truth sets only provide benchmarks for these types of SVs [1]. *Variants detected by single algorithms and combination strategies (referred to as 'TEST' sets) were compared against the truth sets.*

For evaluation, the true positive (TP) was defined if a variant in the 'TRUTH' set was located within the range of +/-500 bp of the 'POS' (start position) of a variant in the 'TEST' set and shared the same SV type. False positives (FP) were calculated as the total variant count of the 'TEST' set minus TP, and false negatives (FN) were calculated as the total variant count of the 'TRUTH' set minus TP. Precision, recall, and F1 score were then calculated using the following formulas.

$\text{precision=}\frac{\text{TP}}{\text{TP + FP}}$ (1)

$recall=\frac{\mathrm{TP}}{TP+FN}$ (2)

$F1=2\times\frac{precison\times recall}{precision+recall}$ (3)

To determine the rank of overall performance of each single caller and combination strategies, combined precision score (cPr), combined recall score (cRc), and combined F1 score (cF1), in which the values for all four data sets were integrated, and were calculated by micro average as follows:

$\text{cPr=}\frac{\text{TP1+TP2+TP3+TP4}}{\text{TP1+TP2+TP3+TP4 + FP1+FP2+FP3+FP4}}$ (4)

$cRc=\frac{\text{TP1+TP2+TP3+TP4}}{\text{TP1+TP2+TP3+TP4}+\text{FN1+FN2+FN3+FN4}}$ (5)

$cF1=2\times\frac{cPr\times cRc}{cPr+cRc}$ (6)

8. The term ‘INFOa’ on page 8 of the manuscript should be corrected to ‘INFO’.

Response:

We sincerely appreciate reviewer for pointing out this error and apologize for the typo. We have corrected the term 'INFOa' to 'INFO' in the ‘Methods’ section of the revised manuscript.

***Page 8:***

***Materials and Methods***

**Combination strategies of multiple algorithms**

Individual SVs were annotated with the name of their respective SV detection algorithms in the *'INFO~~a~~'* field (e.g., caller=DELLY). For grouping, one or more variants from different SV callers were considered a neighbor group if they existed within the range of +/- 500 bp of 'POS' (start position) and shared the same 'SVTYPE.' When referring to candidate SVs within a neighbor group, we retained the variant with the smallest start position as the representative.

9. The representation of recall, precision, and F1 scores should be consistent. For example, if the F1 score is 0.67, then the recall should be represented as 0.63, not as a percentage like 62.57.

Response:

Thank you for your valuable comment. We have ensured consistency in the representation of recall, precision, and F1 scores throughout the manuscript. All values are now presented uniformly in decimal form. Again, we sincerely thank you for your valuable comments.
