# Supplementary material for: Comparisons of performances of structural variants detection algorithms in solitary or combination strategy
Source: PLoS One. 2025 Feb 6;20(2):e0314982. doi: 10.1371/journal.pone.0314982 (PMC11801633; doi:10.1371/journal.pone.0314982)
Supplement: Attachment — Submitted filename: Response to Reviewers.docx [file pone.0314982.s017.docx]

Submission ID PONE-D-24-11846

**Comparisons of performances of Structural Variants Detection Algorithms in Solitary or Combination Strategy**

Point-by-point Response to the Editor and Reviewers

We greatly thank the editor and reviewers for the thoughtful comments of our manuscript. Following with all your kind suggestions, we have made improvement to our manuscript. The detailed point-by-point response to the comments are listed on the following pages. All the changes in revised manuscript are indicated by red color, and we also provide a clear version of our manuscript in the submission package. We really hope the revision will make the paper be more suitable for publication in ***PLOS ONE*** and look forward to hear from you in the near future. Your kind consideration will be highly appreciated!

***Editor***

Comments to the Author

Dear Dr. Chen,

Thank you for submitting your manuscript to PLOS ONE. After careful consideration, we feel that it has merit but does not fully meet PLOS ONE’s publication criteria as it currently stands. Therefore, we invite you to submit a revised version of the manuscript that addresses the points raised during the review process.
Please submit your revised manuscript by Dec 06 2024 11:59PM. If you will need more time than this to complete your revisions, please reply to this message or contact the journal office at [plosone@plos.org](mailto:plosone@plos.org). When you're ready to submit your revision, log on to <https://www.editorialmanager.com/pone/> and select the 'Submissions Needing Revision' folder to locate your manuscript file.

Please include the following items when submitting your revised manuscript:

- A rebuttal letter that responds to each point raised by the academic editor and reviewer(s). You should upload this letter as a separate file labeled 'Response to Reviewers'.
- A marked-up copy of your manuscript that highlights changes made to the original version. You should upload this as a separate file labeled 'Revised Manuscript with Track Changes'.
- An unmarked version of your revised paper without tracked changes. You should upload this as a separate file labeled 'Manuscript'.

If you would like to make changes to your financial disclosure, please include your updated statement in your cover letter. Guidelines for resubmitting your figure files are available below the reviewer comments at the end of this letter.

If applicable, we recommend that you deposit your laboratory protocols in [protocols.io](http://protocols.io) to enhance the reproducibility of your results. Protocols.io assigns your protocol its own identifier (DOI) so that it can be cited independently in the future. For instructions see: [https://journals.plos.org/plosone/s/submission-guidelines#loc-laboratory-protocols](https://c05y1x9s.r.us-east-2.awstrack.me/L0/https:%2F%2Fjournals.plos.org%2Fplosone%2Fs%2Fsubmission-guidelines%23loc-laboratory-protocols/1/010f0192b4c99716-7680738c-4034-4d9d-bdbd-4cd2f2147805-000000/oRtkMyuvjqVqS6KDtXBBB5fT2OQ=182). Additionally, PLOS ONE offers an option for publishing peer-reviewed Lab Protocol articles, which describe protocols hosted on [protocols.io](http://protocols.io). Read more information on sharing protocols at [https://plos.org/protocols?utm_medium=editorial-email&utm_source=authorletters&utm_campaign=protocols](https://c05y1x9s.r.us-east-2.awstrack.me/L0/https:%2F%2Fplos.org%2Fprotocols%3Futm_medium=editorial-email%26utm_source=authorletters%26utm_campaign=protocols/1/010f0192b4c99716-7680738c-4034-4d9d-bdbd-4cd2f2147805-000000/AGamdhia7oXK29bs2ecD44lCcQM=182).

We look forward to receiving your revised manuscript.

Kind regards,

Achraf El Allali, PhD
Academic Editor

Journal Requirements:

Please review your reference list to ensure that it is complete and correct. If you have cited papers that have been retracted, please include the rationale for doing so in the manuscript text, or remove these references and replace them with relevant current references. Any changes to the reference list should be mentioned in the rebuttal letter that accompanies your revised manuscript. If you need to cite a retracted article, indicate the article’s retracted status in the References list and also include a citation and full reference for the retraction notice.

**Response:**

Thank you for the editor’s comments. We have carefully considered all the comment and revised our manuscript accordingly. A detailed, point-by-point response to each comment is provided on the following pages and labeled as 'Response to Reviewers'. All changes in the revised manuscript are indicated in red in the document labeled 'Revised Manuscript with Track Changes'. We also provide a clean version of our manuscript in the submission package, labeled 'Manuscript'.

Following your helpful reminder and reviewer’s suggestion, we have carefully reviewed and reordered the reference list. Any adjustments made to the references are noted in this response letter, as suggested, and these changes have been fully incorporated into the revised manuscript. We appreciate your guidance on this matter.

Additionally, we appreciate the recommendation regarding the use of protocols.io, and we will consider depositing our laboratory protocols there as suggested. We sincerely thank the editor again for the valuable feedback and consideration.

Additional Editor Comments:
The reviewers have finished their review and have minor comments that I include here.

**Response:**

We sincerely thank the editor for the insightful comments and constructive suggestions. We have carefully considered all the reviewers' feedback and revised our manuscript accordingly. We also appreciate your mention of machine learning (ML)-based algorithms. Following your suggestion, we have incorporated discussions on these ML-based algorithms. Below, we provide detailed responses to each comment and outline the changes made. Thank you again for your insightful comments.

R1
The authors address properly my previous comments. As a final note, please make sure that the final versions of the figures have good quality and try to increase font sizes as much as possible. In particular the numbers in figure 4 are very difficult to see.

**Response:**

We sincerely appreciate the detailed review and the valuable comments provided by the reviewer. Following the reviewer’s constructive suggestions, we have adjusted all figures, with particular attention to Figure 4, to ensure they are of high quality and enhance readability. Additionally, all figures have been reviewed using the PACE tool to confirm they meet PLOS requirements. We sincerely thank the reviewer again for the helpful feedback.

***Fig. 1***

**Fig 1. SV detection workflow.** SV detection workflow start*s* with benchmark set collection from GIAB and HGSVC2, followed by sequence alignment using BWA-MEM to produce BAM files. These BAM files are then processed by various SV callers (DRAGEN, Manta, DELLY, LUMPY, GRIDSS, SvABA) to generate VCF files, which are subsequently used for performance assessment, including single tool and combination strategies, based on recall, precision, and F1 score.

***Fig. 2***

**Fig 2. The SVs detected by six SV callers. (A)** SVs of sizes ≥ 50 bp and CTX detected by individual SV callers in samples, HG002, HG00514, HG00733, and NA19240. **(B)** Length distribution of different variants for all samples detected by individual SV callers. The maximum, minimum, and median are based on the integrated values from all sample sets. DEL: deletion, INS: insertion, DUP: duplication, INV: inversion, and CTX: complex translocation.

***Fig. 3***

**Fig 3. The distribution of SVs in truth sets and the performance of individual algorithms. (A)** The distribution of SVs size and types in truth sets. **(B)** The comparison of False negative (FN) and False positive (FP) numbers among individual algorithms. **(C)** The precision, recall, and F1 score of each individual algorithm in detecting “DELs” and “INSs” with the size ≥ 50 bp. DEL: deletion, INS: insertion.

***Fig. 4***

**Fig 4. Concordance of neighbor SVs detected by member callers in different combination strategies. (A)** The agreement among three SV detection tools (Manta, DELLY, and GRIDSS). **(B)** The agreement among five SV detection tools (Manta, DELLY, GRIDSS, LUMPY, and SvABA).

***Fig. 5***

**Fig 5. Distribution and performance of combination strategies. (A)** The distribution of SVs in different combination strategies. **(B)** The comparison of False negative (FN) and False positive (FP) numbers among individual algorithms. **(C)** The precision, recall, and F1 score of combination strategies in the detection of DELs and INSs. DEL: deletion, INS: insertions, DUP: duplication, INV: inversion.

***Fig. 6***

**Fig 6. Combination performance of each single caller and combination strategy in detecting DELs and INSs.** The maximum, minimum, and macro average of recall, precision, and F1 score are based on the integrated values from all sample sets.

R2
1. Please conduct a thorough review of the format and text throughout the document. Specifically, the citation at line 75 should be placed before the period, and the comma usage at line 274 needs to be corrected.

**Response:**

We sincerely appreciate the detailed review and the valuable comments provided by the reviewer. We have conducted a thorough review of the format and text throughout the document. The citation at line 75 has been moved before the period, and the comma usage at line 274 has been corrected. We have also double-checked for any additional formatting and punctuation issues to ensure clarity and adherence to style guidelines. Thank you once again for your kind reminder.

***Page 4:***

***Introduction***

Recently, long-read technologies such as Oxford Nanopore Technologies (ONT) and PacBio HiFi sequencing have made remarkable advancements, becoming more accessible and offering higher accuracy. These improvements help overcome many of the limitations associated with short-read sequencing *[11].*

***Page 11:***

***Results***

***Evaluation of SV detection methods based on the GIAB benchmark set and well referenced cell lines***

Based on the reference settings consistent with the truth sets*,~~，~~*the raw reads underwent alignment to the GRCh37 (for HG002) and hg38 (for HG00514, HG00733, and NA19240 ) version of the human reference genome using BWA-MEM followed by our analysis workflow (Fig 1).

2. I recommend reordering the references to align as closely as possible with the order in which they appear in the text.

**Response:**

We sincerely appreciate the reviewer’s recommendation regarding the reference order. Following reviewer’s suggestion, we have reordered the references to align as closely as possible with their order of appearance in the text. Additionally, following editor’s reminder, we have carefully reviewed the reference list. The new added and corrected references are noted in red in 'Revised Manuscript with Track Changes', as suggested. We appreciate your guidance on this matter. Thank you for your valuable input to improve the clarity and flow of the manuscript.

***Page 3:***

***Introduction***

……Owing to their large size, SVs account for a greater proportion of heritable sequence differences between individuals compared to single nucleotide variants (SNVs), with SVs constituting 0.5–1% of the genome and SNVs approximately 0.1% *[2]*…….

***Page 3:***

***Introduction***

……With the advent of next-generation sequencing (NGS), the detection of SNVs and small indels (<50 bp) has become highly accurate, with an error rate of less than 0.1%. Despite substantial advancements in numerous SV detection methods, their effectiveness in short-read sequences remains limited *[5]*…….

***Page 29:***

***References***

1.	Zook JM, Hansen NF, Olson ND, Chapman L, Mullikin JC, Xiao C, et al. A robust benchmark for detection of germline large deletions and insertions. Nat Biotechnol. 2020;38(11):1347-55. Epub 20200615. doi: 10.1038/s41587-020-0538-8. PubMed PMID: 32541955; PubMed Central PMCID: PMCPMC8454654.

*2.	Pang AW, MacDonald JR, Pinto D, Wei J, Rafiq MA, Conrad DF, et al. Towards a comprehensive structural variation map of an individual human genome. Genome Biology. 2010;11(5):R52. doi: 10.1186/gb-2010-11-5-r52.*

3.	Weischenfeldt J, Symmons O, Spitz F, Korbel JO. Phenotypic impact of genomic structural variation: insights from and for human disease. Nat Rev Genet. 2013;14(2):125-38. doi: 10.1038/nrg3373. PubMed PMID: 23329113.

4.	Collins RL, Brand H, Karczewski KJ, Zhao X, Alföldi J, Francioli LC, et al. A structural variation reference for medical and population genetics. Nature. 2020;581(7809):444-51. Epub 20200527. doi: 10.1038/s41586-020-2287-8. PubMed PMID: 32461652; PubMed Central PMCID: PMCPMC7334194.

*5.	Mahmoud M, Gobet N, Cruz-Dávalos DI, Mounier N, Dessimoz C, Sedlazeck FJ. Structural variant calling: the long and the short of it. Genome Biology. 2019;20(1):246. doi: 10.1186/s13059-019-1828-7.*

6.	Guan P, Sung WK. Structural variation detection using next-generation sequencing data: A comparative technical review. Methods. 2016;102:36-49. Epub 20160201. doi: 10.1016/j.ymeth.2016.01.020. PubMed PMID: 26845461.

7.	Lin K, Smit S, Bonnema G, Sanchez-Perez G, de Ridder D. Making the difference: integrating structural variation detection tools. Briefings in Bioinformatics. 2014;16(5):852-64. doi: 10.1093/bib/bbu047.

8.	Seo JS, Rhie A, Kim J, Lee S, Sohn MH, Kim CU, et al. De novo assembly and phasing of a Korean human genome. Nature. 2016;538(7624):243-7. Epub 20161005. doi: 10.1038/nature20098. PubMed PMID: 27706134.

9.	Pendleton M, Sebra R, Pang AW, Ummat A, Franzen O, Rausch T, et al. Assembly and diploid architecture of an individual human genome via single-molecule technologies. Nat Methods. 2015;12(8):780-6. Epub 20150629. doi: 10.1038/nmeth.3454. PubMed PMID: 26121404; PubMed Central PMCID: PMCPMC4646949.

10.	Chaisson MJ, Huddleston J, Dennis MY, Sudmant PH, Malig M, Hormozdiari F, et al. Resolving the complexity of the human genome using single-molecule sequencing. Nature. 2015;517(7536):608-11. Epub 20141110. doi: 10.1038/nature13907. PubMed PMID: 25383537; PubMed Central PMCID: PMCPMC4317254.

11.	Espinosa E, Bautista R, Larrosa R, Plata O. Advancements in long-read genome sequencing technologies and algorithms. Genomics. 2024;116(3):110842. doi: https://doi.org/10.1016/j.ygeno.2024.110842.

12.	De Coster W, Weissensteiner MH, Sedlazeck FJ. Towards population-scale long-read sequencing. Nature Reviews Genetics. 2021;22(9):572-87. doi: 10.1038/s41576-021-00367-3.

13.	Amarasinghe SL, Su S, Dong X, Zappia L, Ritchie ME, Gouil Q. Opportunities and challenges in long-read sequencing data analysis. Genome Biology. 2020;21(1):30. doi: 10.1186/s13059-020-1935-5.

14.	Halldorsson BV, Eggertsson HP, Moore KHS, Hauswedell H, Eiriksson O, Ulfarsson MO, et al. The sequences of 150,119 genomes in the UK Biobank. Nature. 2022;607(7920):732-40. doi: 10.1038/s41586-022-04965-x.

15.	Mahmoud M, Huang Y, Garimella K, Audano PA, Wan W, Prasad N, et al. Utility of long-read sequencing for All of Us. Nature Communications. 2024;15(1):837. doi: 10.1038/s41467-024-44804-3.

16.	Rausch T, Zichner T, Schlattl A, Stütz AM, Benes V, Korbel JO. DELLY: structural variant discovery by integrated paired-end and split-read analysis. Bioinformatics. 2012;28(18):i333-i9. doi: 10.1093/bioinformatics/bts378. PubMed PMID: 22962449; PubMed Central PMCID: PMCPMC3436805.

17.	Layer RM, Chiang C, Quinlan AR, Hall IM. LUMPY: a probabilistic framework for structural variant discovery. Genome Biol. 2014;15(6):R84. Epub 20140626. doi: 10.1186/gb-2014-15-6-r84. PubMed PMID: 24970577; PubMed Central PMCID: PMCPMC4197822.

18.	Chen X, Schulz-Trieglaff O, Shaw R, Barnes B, Schlesinger F, Källberg M, et al. Manta: rapid detection of structural variants and indels for germline and cancer sequencing applications. Bioinformatics. 2016;32(8):1220-2. Epub 20151208. doi: 10.1093/bioinformatics/btv710. PubMed PMID: 26647377.

19.	Cameron DL, Schröder J, Penington JS, Do H, Molania R, Dobrovic A, et al. GRIDSS: sensitive and specific genomic rearrangement detection using positional de Bruijn graph assembly. Genome Res. 2017;27(12):2050-60. Epub 20171102. doi: 10.1101/gr.222109.117. PubMed PMID: 29097403; PubMed Central PMCID: PMCPMC5741059.

20.	Wala JA, Bandopadhayay P, Greenwald NF, O'Rourke R, Sharpe T, Stewart C, et al. SvABA: genome-wide detection of structural variants and indels by local assembly. Genome Res. 2018;28(4):581-91. Epub 20180313. doi: 10.1101/gr.221028.117. PubMed PMID: 29535149; PubMed Central PMCID: PMCPMC5880247.

21.	Pabinger S, Dander A, Fischer M, Snajder R, Sperk M, Efremova M, et al. A survey of tools for variant analysis of next-generation genome sequencing data. Brief Bioinform. 2014;15(2):256-78. Epub 20130121. doi: 10.1093/bib/bbs086. PubMed PMID: 23341494; PubMed Central PMCID: PMCPMC3956068.

22.	Guan P, Sung W-K. Structural variation detection using next-generation sequencing data: A comparative technical review. Methods. 2016;102:36-49. doi: https://doi.org/10.1016/j.ymeth.2016.01.020.

23.	Escaramís G, Docampo E, Rabionet R. A decade of structural variants: description, history and methods to detect structural variation. Briefings in Functional Genomics. 2015;14(5):305-14. doi: 10.1093/bfgp/elv014.

24.	Sarwal V, Niehus S, Ayyala R, Kim M, Sarkar A, Chang S, et al. A comprehensive benchmarking of WGS-based deletion structural variant callers. Brief Bioinform. 2022;23(4). doi: 10.1093/bib/bbac221. PubMed PMID: 35753701; PubMed Central PMCID: PMCPMC9294411.

25.	Cameron DL, Di Stefano L, Papenfuss AT. Comprehensive evaluation and characterisation of short read general-purpose structural variant calling software. Nature Communications. 2019;10(1):3240. doi: 10.1038/s41467-019-11146-4.

26.	Kosugi S, Momozawa Y, Liu X, Terao C, Kubo M, Kamatani Y. Comprehensive evaluation of structural variation detection algorithms for whole genome sequencing. Genome Biology. 2019;20(1):117. doi: 10.1186/s13059-019-1720-5.

27.	Gong T, Hayes VM, Chan EKF. Detection of somatic structural variants from short-read next-generation sequencing data. Briefings in Bioinformatics. 2020;22(3). doi: 10.1093/bib/bbaa056.

28.	Porubsky D, Ebert P, Audano PA, Vollger MR, Harvey WT, Marijon P, et al. Fully phased human genome assembly without parental data using single-cell strand sequencing and long reads. Nature Biotechnology. 2021;39(3):302-8. doi: 10.1038/s41587-020-0719-5.

29.	Cameron DL, Dong R, Papenfuss AT. StructuralVariantAnnotation: a R/Bioconductor foundation for a caller-agnostic structural variant software ecosystem. Bioinformatics. 2022;38(7):2046-8. doi: 10.1093/bioinformatics/btac042. PubMed PMID: 35134827; PubMed Central PMCID: PMCPMC8963309.

30.	Joe S, Park JL, Kim J, Kim S, Park JH, Yeo MK, et al. Comparison of structural variant callers for massive whole-genome sequence data. BMC Genomics. 2024;25(1):318. Epub 20240328. doi: 10.1186/s12864-024-10239-9. PubMed PMID: 38549092; PubMed Central PMCID: PMCPMC10976732.

31.	Zhuang G, Zhang X, Du W, Xu L, Ma J, Luo H, et al. A benchmarking framework for the accurate and cost-effective detection of clinically-relevant structural variants for cancer target identification and diagnosis. J Transl Med. 2024;22(1):65. Epub 20240116. doi: 10.1186/s12967-024-04865-w. PubMed PMID: 38229122; PubMed Central PMCID: PMCPMC10792779.

32.	van Belzen IAEM, Schönhuth A, Kemmeren P, Hehir-Kwa JY. Structural variant detection in cancer genomes: computational challenges and perspectives for precision oncology. npj Precision Oncology. 2021;5(1):15. doi: 10.1038/s41698-021-00155-6.

33.	Sudmant PH, Rausch T, Gardner EJ, Handsaker RE, Abyzov A, Huddleston J, et al. An integrated map of structural variation in 2,504 human genomes. Nature. 2015;526(7571):75-81. Epub 2015/10/04. doi: 10.1038/nature15394. PubMed PMID: 26432246; PubMed Central PMCID: PMCPMC4617611.

*34.	Li G, Jiang T, Li J, Wang Y. PanSVR: Pan-Genome Augmented Short Read Realignment for Sensitive Detection of Structural Variations. Front Genet. 2021;12:731515. Epub 20210819. doi: 10.3389/fgene.2021.731515. PubMed PMID: 34490049; PubMed Central PMCID: PMCPMC8417358.*

35.	Rajaby R, Liu D-X, Au CH, Cheung Y-T, Lau AYT, Yang Q-Y, Sung W-K. INSurVeyor: improving insertion calling from short read sequencing data. Nature Communications. 2023;14(1):3243. doi: 10.1038/s41467-023-38870-2.

*36.	Chiara M, Pesole G, Horner DS. SVM 2 : an improved paired-end-based tool for the detection of small genomic structural variations using high-throughput single-genome resequencing data. Nucleic Acids Research. 2012;40(18):e145-e. doi: 10.1093/nar/gks606.*

*37.	Michaelson JJ, Sebat J. forestSV: structural variant discovery through statistical learning. Nature Methods. 2012;9(8):819-21. doi: 10.1038/nmeth.2085.*

*38.	Kronenberg ZN, Osborne EJ, Cone KR, Kennedy BJ, Domyan ET, Shapiro MD, et al. Wham: Identifying Structural Variants of Biological Consequence. PLoS Comput Biol. 2015;11(12):e1004572. Epub 20151201. doi: 10.1371/journal.pcbi.1004572. PubMed PMID: 26625158; PubMed Central PMCID: PMCPMC4666669.*

*39.	Parikh H, Mohiyuddin M, Lam HYK, Iyer H, Chen D, Pratt M, et al. svclassify: a method to establish benchmark structural variant calls. BMC Genomics. 2016;17(1):64. doi: 10.1186/s12864-016-2366-2.*

*40.	Alzaid E, El Allali A, Aboalsamh H. A Classification Approach for Genome Structural Variations Detection. Journal of Proteomics & Bioinformatics. 2018;11:211-8.*

*41.	Bartenhagen C, Dugas M. Robust and exact structural variation detection with paired-end and soft-clipped alignments: SoftSV compared with eight algorithms. Brief Bioinform. 2016;17(1):51-62. Epub 20150520. doi: 10.1093/bib/bbv028. PubMed PMID: 25998133.*

*42.	Popic V, Rohlicek C, Cunial F, Hajirasouliha I, Meleshko D, Garimella K, Maheshwari A. Cue: a deep-learning framework for structural variant discovery and genotyping. Nature Methods. 2023;20(4):559-68. doi: 10.1038/s41592-023-01799-x.*

43.	Zhuang G, Zhang X, Du W, Xu L, Ma J, Luo H, et al. A benchmarking framework for the accurate and cost-effective detection of clinically-relevant structural variants for cancer target identification and diagnosis. Journal of Translational Medicine. 2024;22(1):65. doi: 10.1186/s12967-024-04865-w.

*44.	Sedlazeck FJ, Rescheneder P, Smolka M, Fang H, Nattestad M, von Haeseler A, Schatz MC. Accurate detection of complex structural variations using single-molecule sequencing. Nature Methods. 2018;15(6):461-8. doi: 10.1038/s41592-018-0001-7.*

*45.	Gaitán N, Duitama J. A graph clustering algorithm for detection and genotyping of structural variants from long reads. GigaScience. 2024;13. doi: 10.1093/gigascience/giad112.*

*46.	Jiang T, Liu Y, Jiang Y, Li J, Gao Y, Cui Z, et al. Long-read-based human genomic structural variation detection with cuteSV. Genome Biology. 2020;21(1):189. doi: 10.1186/s13059-020-02107-y.*

*47.	Mahmoud M, Gobet N, Cruz-Dávalos DI, Mounier N, Dessimoz C, Sedlazeck FJ. Structural variant calling: the long and the short of it. Genome Biol. 2019;20(1):246. Epub 20191120. doi: 10.1186/s13059-019-1828-7. PubMed PMID: 31747936; PubMed Central PMCID: PMCPMC6868818.*

*48.	Heller D, Vingron M. SVIM: structural variant identification using mapped long reads. Bioinformatics. 2019;35(17):2907-15. doi: 10.1093/bioinformatics/btz041. PubMed PMID: 30668829; PubMed Central PMCID: PMCPMC6735718.*

*49.	Zheng Y, Shang X. SVvalidation: A long-read-based validation method for genomic structural variation. PLoS One. 2024;19(1):e0291741. Epub 20240105. doi: 10.1371/journal.pone.0291741. PubMed PMID: 38181020; PubMed Central PMCID: PMCPMC10769053.*

*50.	Zhang Z, Jiang T, Li G, Cao S, Liu Y, Liu B, Wang Y. Kled: an ultra-fast and sensitive structural variant detection tool for long-read sequencing data. Brief Bioinform. 2024;25(2). doi: 10.1093/bib/bbae049. PubMed PMID: 38385878; PubMed Central PMCID: PMCPMC10883419.*

*51.	Hu H, Gao R, Gao W, Gao B, Jiang Z, Zhou M, et al. SVDF: enhancing structural variation detect from long-read sequencing via automatic filtering strategies. Brief Bioinform. 2024;25(4). doi: 10.1093/bib/bbae336. PubMed PMID: 38980375; PubMed Central PMCID: PMCPMC11232458.*

3. The manuscript primarily discusses tools published before 2020; however, significant advancements have been made in SV detection tools based on second-generation sequencing data in recent years. For instance, tools like "PanSVR: Pan-genome augmented short read realignment for sensitive detection of structural variations" and "Psi-Caller: a lightweight short read-based variant caller with high speed and accuracy" represent the latest research outcomes and should be discussed more thoroughly in the text. Additionally, the discussion on third-generation sequencing data is insufficient. It is advisable to incorporate discussions on the latest tools for SV detection using third-generation sequencing data, such as "SVDF: enhancing structural variation detection from long-read sequencing via automatic filtering strategies" and "Kled: an ultra-fast and sensitive structural variant detection tool for long-read sequencing data," to enrich and enhance the discussion section.

In the spirit of what reviewer 2 have mentioned, there are some other tools that use machine learning and post processing approaches such as the following doi: 10.4172/0974-276X.1000488 and <https://doi.org/10.1177/117793221989295>

**Response:**

We sincerely thank the editor and reviewer for the insightful comments and constructive suggestions. Following the recommendations, we have expanded our discussion to include recent advancements in SV detection tools based on short-read sequencing data. For instance, we now discuss "PanSVR," as recommended by the reviewer, which enhances SV calling by incorporating a pan-genome SV reference and read realignment, and "INSurVeyor," which improves the sensitivity of insertion detection from short-read sequencing data. These tools highlight opportunities to integrate algorithms optimized for low-complexity regions and specific SV types, enhancing overall detection accuracy.

Additionally, in response to the reviewer’s suggestion to incorporate a more comprehensive discussion on SV detection tools using third-generation sequencing data, we have included recent tools developed for long-read sequencing (LRS) data, such as "Svvalidator", "Kled” and "SVDF". Svvalidator" and "Kled" captures SV signatures from CIGAR strings and split reads to validate SVs effectively, while "SVDF" employs machine learning (ML)-based adaptive clustering and noise-filtering strategies to enhance SV detection. Integrating short-read and long-read sequencing provide opportunities for developing strategies tailored to diverse research and clinical needs.

Furthermore, in line with the editor’s comments, we have incorporated a discussion on additional tools that leverage ML and post-processing approaches, citing relevant works such as "doi: 10.4172/0974-276X.1000488, " as recommended by the editor. We now discuss "MPRClassify," which uses a multi-part read alignment strategy alongside three random forest classifiers to optimize detection for specific SV types, and "Cue," which transforms sequence alignments into images to highlight SV-related signals, using a convolutional neural network to predict the type, genotype, and genomic location of each SV. Additionally, we reference the work by Guiwu Zhuang et al., who developed a random-forest-based decision model to improve the precision of clinically relevant tumor-specific SV detection by evaluating multiple SV detection algorithms.

The discussion section has been thoroughly revised and now includes references to these relevant tools and studies. Adjustments to the reference list are noted in red in 'Revised Manuscript with Track Changes', as suggested. We appreciate the valuable comments.

***Page 23:***

***Discussion***

SVs can have a substantial phenotypic impact, disrupting gene function, regulation, or modifying gene dosage. Their significance in medicine and molecular biology has been underscored by recent studies [33]. The role of SVs in the pathogenicity of genetic diseases is increasingly recognized, and there is a possibility that screening for SVs may become routine in clinical settings in the future.

According to our study, relying on a single algorithm for SV detection may pose the risk of limited SV size and type detection, along with low F1 score. While our multiple agreement strategy, which focuses on consensus among algorithms, tends to yield higher precision at the cost of lower recall, employing a union combination enhanced recall and F1 scores for DELs compared to both single algorithms and the multiple agreement approach. However, most callers show poor F1 scores in INS detection due to low recall rates.  Specifically, DELLY (0.02) and GRIDSS (0.01) exhibited very poor F1 scores, while Manta (0.30) were the top performers among all non-commercial tools. The union strategy, which includes Manta, consequently achieved a comparable F1 score for INS detection, as illustrated in (Fig 6). This underscores the importance of choosing well-performing callers tailored to specific types and sizes of SVs for different clinical scenarios.

As for the concern of FP variants that may *could* be falsely related to a phenotype. We were particularly interested in FPs that are consistently detected by multiple callers. We analyzed the FPs called by at least two algorithms in the HG002 dataset and compared them with DRAGEN. We differentiated FPs identified only by the multi-agreement strategy (M), only by DRAGEN (D), and those identified by both (Both), the FP SV size displaying the median and interquartile range were shown as S4_figure. We found that the majority of FPs are smaller than 10^3 base pairs. For DEL FPs, the M group had a median SV length of 326 bp, while the D group had a median of 120 bp. As for INSs, due to the poor recall of most tools, the number of FPs was quite low, with D group INS FPs having a median SV length of 81 bp. Considering that most short reads range from 300 to 500 bp in length, we believe they should be capable of accurately sequencing variants of this small size. Each tool has its own underlying principles and parameters for detecting SVs, as outlined in their respective publications, many employ hard filters—such as thresholds for variant quality, and read depth—to reduce FPs. However, care is needed to balance filtering to avoid discarding true positives. In clinical practice, post-processing tasks like filtering target genes using a virtual panel, along with additional validation through the Integrative Genomics Viewer (IGV) or methods such as PCR, are commonly used. Therefore, weighing these considerations, we believe that achieving a high recall rate to minimize FN enhance*s* the utility of these findings in clinical settings.

Several studies *have* also shown that the occurrence of FPs in short-read sequencing for SV identification can be influenced by various factors, affecting overall precision. For example, Daniel L. Cameron et al. assessed the impact of sequence context and event size on the precision of 10 SV callers, evaluating these tools using simulation data and well-referenced cell lines. Their study included several tools that we also selected, such as Manta, DELLY, GRIDSS, and LUMPY. They identified several major factors *that* impact the precision: 1) The proximity of SNVs or indels near the SV breakpoints. When multiple flanking variants are present, false positive rates increase. 2) SVs located in low-complexity and simple tandem repeat regions are prone to higher false positive rates. SVs within LTR, LINEs, and SINEs regions also show reduced precision, leading to more false positives. 3) The size of the event also plays a role; they indicated SVs smaller than 100 bp are particularly challenging, whereas deletions between 300 to 500 bp tend to achieve higher precision.

*SV detection tools based on second-generation sequencing short read data have continued to advance in recent years. Recently, Gaoyang Li et al. published PanSVR [34], which enhances SV calling by incorporating a pan-genome SV reference and read re-alignment. By leveraging SNPs and INDELs in variable number tandem repeat (VNTR) and short tandem repeat (STR) regions, PanSVR improves alignment accuracy in these regions. Furthermore, with the integration of pan-genome reference information, PanSVR enhances recall for long insertions, showcasing the advantages of a pan-genome-based approach. In their benchmark study, PanSVR achieved higher F1 scores than Manta and Delly for detecting both DELs and INSs. Moreover,* Ramesh Rajaby and colleagues developed an INS caller, INSurVeyor [35], which improves the sensitivity of INS calling from short-read sequencing data, performing even better than Manta. This highlights opportunities to integrate better-performing algorithms tailored to low-complexity regions and specific SV types, like using *PanSVR* or INSurVeyor in combination with union strategies for enhanced performance.

*Additionally, research on applying supervised learning techniques for SV detection has been growing, with notable classification-based tools such as SVM2 [36], ForestSV [37], Wham [38], and Svclassify [39]. These tools employ distinct techniques and primarily utilize information extracted from RP signals to predict candidate SV regions. Recently, several machine learning (ML)-based approaches have demonstrated impressive performance. Eman A. Alzaid and colleagues developed MPRClassify [40], which uses a multi-part read alignment strategy with three independent random forest classifiers optimized to detect specific SV types in candidate SV regions. This approach relies solely on single reads, eliminating the need for paired-end read data. Experimental results indicated that single reads can effectively identify SVs without paired-end signatures, achieving performance comparable to existing methods, such as DELLY, Manta, and Softsv [41]. Victoria Popic et al. recently developed Cue [42], which transforms sequence alignments into images that highlight SV-related signals and utilizes a stacked hourglass convolutional neural network to predict the type, genotype, and genomic location of each SV. Evaluations on both synthetic and real short-read datasets demonstrated Cue's superior performance over established methods like Manta, DELLY, LUMPY, and SvABA, particularly in detecting a variety of SV classes, including DELs, DUPs, and INVs. In a*nother recent study, Guiwu Zhuang et al. [43] established an expert-reviewed tumor-specific clinically relevant SV call set, evaluating several algorithms for SV detection, and developed a random-forest-based decision model to improve the precision. Building on these research foundations, it is worth exploring the potential of integrating the union combination strategy with an ML*-*based decision model in the future. For practical clinical applications, further investigation into the performance using real clinical sample sets is necessary.

Finally, when addressing SV calling, the role of long-read sequencing cannot be overlooked. Long-read sequencing (*LRS*) offers remarkable advantages as it can span repetitive or other problematic regions. For applications that require identifying very long or complex SVs, performing a localized long-read de novo assembly is more effective than conventional short-read mapping approaches. Multiple studies have demonstrated that long-read sequencing technologies offer superior accuracy in SV detection compared to short-read approaches. Benchmarking results, as shown in recent studies [44-46], consistently highlight the advantages of long reads in terms of sensitivity and specificity, regardless of the specific tools used. Moreover, the recent advancements in long-read technologies and the ongoing reductions in sequencing costs—led by both Oxford Nanopore and PacBio—have made long-read sequencing increasingly accessible and widespread. *SV detection methods and post- processing approaches based on LRS have been developed to date, primarily relying on alignment-based methods that use manually designed heuristic rules tailored to sequencing platform characteristics [46-50]. For instance, Svvalidator [49] and Kled [50] both collect signatures from CIGAR strings and split reads to validate SVs, with Kled further employing an Omni Merging Algorithm (OMA) to refine and genotype clusters efficiently through multi-threading and optimized memory usage in C++. However, manually designed heuristics can introduce noise and false positives, a limitation that ML-based approaches like SVDF [51] addressed by employing adaptive clustering and noise-filtering strategies.* Integrating short-read and long-read sequencing provide*s* opportunities for developing strategies tailored to diverse research and clinical needs.

Although our study only focused on the DELs and INSs— which are the majority but relatively simple SV types in the human genome— methods failing to detect DELs and INSs might also be less effective at identifying more complex SV types. Currently, our study evaluated the ability of various methods and combination strategies to detect DELs and INSs, providing a valuable and optimistic perspective on overall SV detection performance.

***Page 31:***

***References***

*34. Li G, Jiang T, Li J, Wang Y. PanSVR: Pan-Genome Augmented Short Read Realignment for Sensitive Detection of Structural Variations. Front Genet. 2021;12:731515. Epub 20210819. doi: 10.3389/fgene.2021.731515. PubMed PMID: 34490049; PubMed Central PMCID: PMCPMC8417358.*

*35.	Rajaby R, Liu D-X, Au CH, Cheung Y-T, Lau AYT, Yang Q-Y, Sung W-K. INSurVeyor: improving insertion calling from short read sequencing data. Nature Communications. 2023;14(1):3243. doi: 10.1038/s41467-023-38870-2.*

*36.	Chiara M, Pesole G, Horner DS. SVM 2 : an improved paired-end-based tool for the detection of small genomic structural variations using high-throughput single-genome resequencing data. Nucleic Acids Research. 2012;40(18):e145-e. doi: 10.1093/nar/gks606.*

*37.	Michaelson JJ, Sebat J. forestSV: structural variant discovery through statistical learning. Nature Methods. 2012;9(8):819-21. doi: 10.1038/nmeth.2085.*

*38.	Kronenberg ZN, Osborne EJ, Cone KR, Kennedy BJ, Domyan ET, Shapiro MD, et al. Wham: Identifying Structural Variants of Biological Consequence. PLoS Comput Biol. 2015;11(12):e1004572. Epub 20151201. doi: 10.1371/journal.pcbi.1004572. PubMed PMID: 26625158; PubMed Central PMCID: PMCPMC4666669.*

*39.	Parikh H, Mohiyuddin M, Lam HYK, Iyer H, Chen D, Pratt M, et al. svclassify: a method to establish benchmark structural variant calls. BMC Genomics. 2016;17(1):64. doi: 10.1186/s12864-016-2366-2.*

*40.	Alzaid E, El Allali A, Aboalsamh H. A Classification Approach for Genome Structural Variations Detection. Journal of Proteomics & Bioinformatics. 2018;11:211-8.*

*41.	Bartenhagen C, Dugas M. Robust and exact structural variation detection with paired-end and soft-clipped alignments: SoftSV compared with eight algorithms. Brief Bioinform. 2016;17(1):51-62. Epub 20150520. doi: 10.1093/bib/bbv028. PubMed PMID: 25998133.*

*42.	Popic V, Rohlicek C, Cunial F, Hajirasouliha I, Meleshko D, Garimella K, Maheshwari A. Cue: a deep-learning framework for structural variant discovery and genotyping. Nature Methods. 2023;20(4):559-68. doi: 10.1038/s41592-023-01799-x.*

*43.	Zhuang G, Zhang X, Du W, Xu L, Ma J, Luo H, et al. A benchmarking framework for the accurate and cost-effective detection of clinically-relevant structural variants for cancer target identification and diagnosis. Journal of Translational Medicine. 2024;22(1):65. doi: 10.1186/s12967-024-04865-w.*

*44.	Sedlazeck FJ, Rescheneder P, Smolka M, Fang H, Nattestad M, von Haeseler A, Schatz MC. Accurate detection of complex structural variations using single-molecule sequencing. Nature Methods. 2018;15(6):461-8. doi: 10.1038/s41592-018-0001-7.*

*45.	Gaitán N, Duitama J. A graph clustering algorithm for detection and genotyping of structural variants from long reads. GigaScience. 2024;13. doi: 10.1093/gigascience/giad112.*

*46.	Jiang T, Liu Y, Jiang Y, Li J, Gao Y, Cui Z, et al. Long-read-based human genomic structural variation detection with cuteSV. Genome Biology. 2020;21(1):189. doi: 10.1186/s13059-020-02107-y.*

*47.	Mahmoud M, Gobet N, Cruz-Dávalos DI, Mounier N, Dessimoz C, Sedlazeck FJ. Structural variant calling: the long and the short of it. Genome Biol. 2019;20(1):246. Epub 20191120. doi: 10.1186/s13059-019-1828-7. PubMed PMID: 31747936; PubMed Central PMCID: PMCPMC6868818.*

*48.	Heller D, Vingron M. SVIM: structural variant identification using mapped long reads. Bioinformatics. 2019;35(17):2907-15. doi: 10.1093/bioinformatics/btz041. PubMed PMID: 30668829; PubMed Central PMCID: PMCPMC6735718.*

*49.	Zheng Y, Shang X. SVvalidation: A long-read-based validation method for genomic structural variation. PLoS One. 2024;19(1):e0291741. Epub 20240105. doi: 10.1371/journal.pone.0291741. PubMed PMID: 38181020; PubMed Central PMCID: PMCPMC10769053.*

*50.	Zhang Z, Jiang T, Li G, Cao S, Liu Y, Liu B, Wang Y. Kled: an ultra-fast and sensitive structural variant detection tool for long-read sequencing data. Brief Bioinform. 2024;25(2). doi: 10.1093/bib/bbae049. PubMed PMID: 38385878; PubMed Central PMCID: PMCPMC10883419.*

*51.	Hu H, Gao R, Gao W, Gao B, Jiang Z, Zhou M, et al. SVDF: enhancing structural variation detect from long-read sequencing via automatic filtering strategies. Brief Bioinform. 2024;25(4). doi: 10.1093/bib/bbae336. PubMed PMID: 38980375; PubMed Central PMCID: PMCPMC11232458.*

Since Plos one does not have a proofreading step, please check all the grammar and make sure all figures have the best quality possible.

**Response:**

We appreciate the editor's detailed review and helpful reminder. We have thoroughly checked the grammar using the tool “Grammarly,” and all figures have been reviewed using the PACE tool to confirm they meet PLOS requirements. We sincerely thank the editor once again for the valuable feedback.

***Reviewers' comments:***

Reviewer's Responses to Questions

Comments to the Author

1. If the authors have adequately addressed your comments raised in a previous round of review and you feel that this manuscript is now acceptable for publication, you may indicate that here to bypass the “Comments to the Author” section, enter your conflict of interest statement in the “Confidential to Editor” section, and submit your "Accept" recommendation.

Reviewer #1: All comments have been addressed

Reviewer #3: All comments have been addressed

**Response:**

We sincerely appreciate the detailed review and the valuable comments provided by the reviewers.

2. Is the manuscript technically sound, and do the data support the conclusions?

The manuscript must describe a technically sound piece of scientific research with data that supports the conclusions. Experiments must have been conducted rigorously, with appropriate controls, replication, and sample sizes. The conclusions must be drawn appropriately based on the data presented

Reviewer #1: Yes

Reviewer #3: Yes

**Response:**

We appreciate the reviewers' detailed review.

3. Has the statistical analysis been performed appropriately and rigorously?

Reviewer #1: Yes

Reviewer #3: Yes

**Response:**

We appreciate the reviewers' detailed review.

4. Have the authors made all data underlying the findings in their manuscript fully available?

The [PLOS Data policy](http://c05y1x9s.r.us-east-2.awstrack.me/L0/http:%2F%2Fwww.plosone.org%2Fstatic%2Fpolicies.action%23sharing/1/010f0192b4c99716-7680738c-4034-4d9d-bdbd-4cd2f2147805-000000/yi7iL0smFGGJLZbFEC6GRuZp9oI=182) requires authors to make all data underlying the findings described in their manuscript fully available without restriction, with rare exception (please refer to the Data Availability Statement in the manuscript PDF file). The data should be provided as part of the manuscript or its supporting information, or deposited to a public repository. For example, in addition to summary statistics, the data points behind means, medians and variance measures should be available. If there are restrictions on publicly sharing data—e.g. participant privacy or use of data from a third party—those must be specified.

Reviewer #1: Yes

Reviewer #3: Yes

**Response:**

We appreciate for your comment!

5. Is the manuscript presented in an intelligible fashion and written in standard English?

PLOS ONE does not copyedit accepted manuscripts, so the language in submitted articles must be clear, correct, and unambiguous. Any typographical or grammatical errors should be corrected at revision, so please note any specific errors here.

Reviewer #1: Yes

Reviewer #3: Yes

**Response:**

We appreciate for your comment!

6. Review Comments to the Author

Please use the space provided to explain your answers to the questions above. You may also include additional comments for the author, including concerns about dual publication, research ethics, or publication ethics. (Please upload your review as an attachment if it exceeds 20,000 characters)

**Response:**

We sincerely appreciate for the detailed review and valuable comment.

7. PLOS authors have the option to publish the peer review history of their article (what does this mean?). If published, this will include your full peer review and any attached files. f you choose “no”, your identity will remain anonymous but your review may still be made public.

Do you want your identity to be public for this peer review? For information about this choice, including consent withdrawal, please see our Privacy Policy.

Reviewer #1: Yes: Jorge Duitama

Reviewer #3: No

[NOTE: If reviewer comments were submitted as an attachment file, they will be attached to this email and accessible via the submission site. Please log into your account, locate the manuscript record, and check for the action link "View Attachments". If this link does not appear, there are no attachment files.]

**Response:**

We sincerely appreciate the detailed review and the valuable comments provided by the reviewers.

**Reviewer #1**

*Comments to the Author*

The authors address properly my previous comments. As a final note, please make sure that the final versions of the figures have good quality and try to increase font sizes as much as possible. In particular the numbers in figure 4 are very difficult to see.

**Response:**

We sincerely appreciate the detailed review and the valuable comments provided by the reviewer. Following the reviewer’s constructive suggestions, we have adjusted all figures, with particular attention to Figure 4, to ensure they are of high quality and enhance readability. Additionally, all figures have been reviewed using the PACE tool to confirm they meet PLOS requirements. We sincerely thank the reviewer again for the helpful feedback.

***Fig. 1***

**Fig 1. SV detection workflow.** SV detection workflow start*s* with benchmark set collection from GIAB and HGSVC2, followed by sequence alignment using BWA-MEM to produce BAM files. These BAM files are then processed by various SV callers (DRAGEN, Manta, DELLY, LUMPY, GRIDSS, SvABA) to generate VCF files, which are subsequently used for performance assessment, including single tool and combination strategies, based on recall, precision, and F1 score.

***Fig. 2***

**Fig 2. The SVs detected by six SV callers. (A)** SVs of sizes ≥ 50 bp and CTX detected by individual SV callers in samples, HG002, HG00514, HG00733, and NA19240. **(B)** Length distribution of different variants for all samples detected by individual SV callers. The maximum, minimum, and median are based on the integrated values from all sample sets. DEL: deletion, INS: insertion, DUP: duplication, INV: inversion, and CTX: complex translocation.

***Fig. 3***

**Fig 3. The distribution of SVs in truth sets and the performance of individual algorithms. (A)** The distribution of SVs size and types in truth sets. **(B)** The comparison of False negative (FN) and False positive (FP) numbers among individual algorithms. **(C)** The precision, recall, and F1 score of each individual algorithm in detecting “DELs” and “INSs” with the size ≥ 50 bp. DEL: deletion, INS: insertion.

***Fig. 4***

**Fig 4. Concordance of neighbor SVs detected by member callers in different combination strategies. (A)** The agreement among three SV detection tools (Manta, DELLY, and GRIDSS). **(B)** The agreement among five SV detection tools (Manta, DELLY, GRIDSS, LUMPY, and SvABA).

***Fig. 5***

**Fig 5. Distribution and performance of combination strategies. (A)** The distribution of SVs in different combination strategies. **(B)** The comparison of False negative (FN) and False positive (FP) numbers among individual algorithms. **(C)** The precision, recall, and F1 score of combination strategies in the detection of DELs and INSs. DEL: deletion, INS: insertions, DUP: duplication, INV: inversion.

***Fig. 6***

**Fig 6. Combination performance of each single caller and combination strategy in detecting DELs and INSs.** The maximum, minimum, and macro average of recall, precision, and F1 score are based on the integrated values from all sample sets.

**Reviewer #3**

*Comments to the Author*

The author has made revisions in response to the issues I raised, however, I still I have the following remaining comments:

1. Please conduct a thorough review of the format and text throughout the document. Specifically, the citation at line 75 should be placed before the period, and the comma usage at line 274 needs to be corrected.

**Response:**

We sincerely appreciate the detailed review and the valuable comments provided by the reviewer. We have conducted a thorough review of the format and text throughout the document. The citation at line 75 has been moved before the period, and the comma usage at line 274 has been corrected. We have also double-checked for any additional formatting and punctuation issues to ensure clarity and adherence to style guidelines. Thank you once again for your kind reminder.

***Page 4:***

***Introduction***

Recently, long-read technologies such as Oxford Nanopore Technologies (ONT) and PacBio HiFi sequencing have made remarkable advancements, becoming more accessible and offering higher accuracy. These improvements help overcome many of the limitations associated with short-read sequencing *[11].*

***Page 11:***

***Results***

***Evaluation of SV detection methods based on the GIAB benchmark set and well referenced cell lines***

Based on the reference settings consistent with the truth sets*,~~，~~*the raw reads underwent alignment to the GRCh37 (for HG002) and hg38 (for HG00514, HG00733, and NA19240 ) version of the human reference genome using BWA-MEM followed by our analysis workflow (Fig 1).

2. I recommend reordering the references to align as closely as possible with the order in which they appear in the text.

**Response:**

We sincerely appreciate the reviewer’s recommendation regarding the reference order. Following reviewer’s suggestion, we have reordered the references to align as closely as possible with their order of appearance in the text. Additionally, following editor’s reminder, we have carefully reviewed the reference list. The new added and corrected references are noted in red in 'Revised Manuscript with Track Changes', as suggested. We appreciate your guidance on this matter. Thank you for your valuable input to improve the clarity and flow of the manuscript.

***Page 3:***

***Introduction***

……Owing to their large size, SVs account for a greater proportion of heritable sequence differences between individuals compared to single nucleotide variants (SNVs), with SVs constituting 0.5–1% of the genome and SNVs approximately 0.1% *[2]*…….

***Page 3:***

***Introduction***

……With the advent of next-generation sequencing (NGS), the detection of SNVs and small indels (<50 bp) has become highly accurate, with an error rate of less than 0.1%. Despite substantial advancements in numerous SV detection methods, their effectiveness in short-read sequences remains limited *[5]*…….

***Page 29:***

***References***

1.	Zook JM, Hansen NF, Olson ND, Chapman L, Mullikin JC, Xiao C, et al. A robust benchmark for detection of germline large deletions and insertions. Nat Biotechnol. 2020;38(11):1347-55. Epub 20200615. doi: 10.1038/s41587-020-0538-8. PubMed PMID: 32541955; PubMed Central PMCID: PMCPMC8454654.

*2.	Pang AW, MacDonald JR, Pinto D, Wei J, Rafiq MA, Conrad DF, et al. Towards a comprehensive structural variation map of an individual human genome. Genome Biology. 2010;11(5):R52. doi: 10.1186/gb-2010-11-5-r52.*

3.	Weischenfeldt J, Symmons O, Spitz F, Korbel JO. Phenotypic impact of genomic structural variation: insights from and for human disease. Nat Rev Genet. 2013;14(2):125-38. doi: 10.1038/nrg3373. PubMed PMID: 23329113.

4.	Collins RL, Brand H, Karczewski KJ, Zhao X, Alföldi J, Francioli LC, et al. A structural variation reference for medical and population genetics. Nature. 2020;581(7809):444-51. Epub 20200527. doi: 10.1038/s41586-020-2287-8. PubMed PMID: 32461652; PubMed Central PMCID: PMCPMC7334194.

*5.	Mahmoud M, Gobet N, Cruz-Dávalos DI, Mounier N, Dessimoz C, Sedlazeck FJ. Structural variant calling: the long and the short of it. Genome Biology. 2019;20(1):246. doi: 10.1186/s13059-019-1828-7.*

6.	Guan P, Sung WK. Structural variation detection using next-generation sequencing data: A comparative technical review. Methods. 2016;102:36-49. Epub 20160201. doi: 10.1016/j.ymeth.2016.01.020. PubMed PMID: 26845461.

7.	Lin K, Smit S, Bonnema G, Sanchez-Perez G, de Ridder D. Making the difference: integrating structural variation detection tools. Briefings in Bioinformatics. 2014;16(5):852-64. doi: 10.1093/bib/bbu047.

8.	Seo JS, Rhie A, Kim J, Lee S, Sohn MH, Kim CU, et al. De novo assembly and phasing of a Korean human genome. Nature. 2016;538(7624):243-7. Epub 20161005. doi: 10.1038/nature20098. PubMed PMID: 27706134.

9.	Pendleton M, Sebra R, Pang AW, Ummat A, Franzen O, Rausch T, et al. Assembly and diploid architecture of an individual human genome via single-molecule technologies. Nat Methods. 2015;12(8):780-6. Epub 20150629. doi: 10.1038/nmeth.3454. PubMed PMID: 26121404; PubMed Central PMCID: PMCPMC4646949.

10.	Chaisson MJ, Huddleston J, Dennis MY, Sudmant PH, Malig M, Hormozdiari F, et al. Resolving the complexity of the human genome using single-molecule sequencing. Nature. 2015;517(7536):608-11. Epub 20141110. doi: 10.1038/nature13907. PubMed PMID: 25383537; PubMed Central PMCID: PMCPMC4317254.

11.	Espinosa E, Bautista R, Larrosa R, Plata O. Advancements in long-read genome sequencing technologies and algorithms. Genomics. 2024;116(3):110842. doi: https://doi.org/10.1016/j.ygeno.2024.110842.

12.	De Coster W, Weissensteiner MH, Sedlazeck FJ. Towards population-scale long-read sequencing. Nature Reviews Genetics. 2021;22(9):572-87. doi: 10.1038/s41576-021-00367-3.

13.	Amarasinghe SL, Su S, Dong X, Zappia L, Ritchie ME, Gouil Q. Opportunities and challenges in long-read sequencing data analysis. Genome Biology. 2020;21(1):30. doi: 10.1186/s13059-020-1935-5.

14.	Halldorsson BV, Eggertsson HP, Moore KHS, Hauswedell H, Eiriksson O, Ulfarsson MO, et al. The sequences of 150,119 genomes in the UK Biobank. Nature. 2022;607(7920):732-40. doi: 10.1038/s41586-022-04965-x.

15.	Mahmoud M, Huang Y, Garimella K, Audano PA, Wan W, Prasad N, et al. Utility of long-read sequencing for All of Us. Nature Communications. 2024;15(1):837. doi: 10.1038/s41467-024-44804-3.

16.	Rausch T, Zichner T, Schlattl A, Stütz AM, Benes V, Korbel JO. DELLY: structural variant discovery by integrated paired-end and split-read analysis. Bioinformatics. 2012;28(18):i333-i9. doi: 10.1093/bioinformatics/bts378. PubMed PMID: 22962449; PubMed Central PMCID: PMCPMC3436805.

17.	Layer RM, Chiang C, Quinlan AR, Hall IM. LUMPY: a probabilistic framework for structural variant discovery. Genome Biol. 2014;15(6):R84. Epub 20140626. doi: 10.1186/gb-2014-15-6-r84. PubMed PMID: 24970577; PubMed Central PMCID: PMCPMC4197822.

18.	Chen X, Schulz-Trieglaff O, Shaw R, Barnes B, Schlesinger F, Källberg M, et al. Manta: rapid detection of structural variants and indels for germline and cancer sequencing applications. Bioinformatics. 2016;32(8):1220-2. Epub 20151208. doi: 10.1093/bioinformatics/btv710. PubMed PMID: 26647377.

19.	Cameron DL, Schröder J, Penington JS, Do H, Molania R, Dobrovic A, et al. GRIDSS: sensitive and specific genomic rearrangement detection using positional de Bruijn graph assembly. Genome Res. 2017;27(12):2050-60. Epub 20171102. doi: 10.1101/gr.222109.117. PubMed PMID: 29097403; PubMed Central PMCID: PMCPMC5741059.

20.	Wala JA, Bandopadhayay P, Greenwald NF, O'Rourke R, Sharpe T, Stewart C, et al. SvABA: genome-wide detection of structural variants and indels by local assembly. Genome Res. 2018;28(4):581-91. Epub 20180313. doi: 10.1101/gr.221028.117. PubMed PMID: 29535149; PubMed Central PMCID: PMCPMC5880247.

21.	Pabinger S, Dander A, Fischer M, Snajder R, Sperk M, Efremova M, et al. A survey of tools for variant analysis of next-generation genome sequencing data. Brief Bioinform. 2014;15(2):256-78. Epub 20130121. doi: 10.1093/bib/bbs086. PubMed PMID: 23341494; PubMed Central PMCID: PMCPMC3956068.

22.	Guan P, Sung W-K. Structural variation detection using next-generation sequencing data: A comparative technical review. Methods. 2016;102:36-49. doi: https://doi.org/10.1016/j.ymeth.2016.01.020.

23.	Escaramís G, Docampo E, Rabionet R. A decade of structural variants: description, history and methods to detect structural variation. Briefings in Functional Genomics. 2015;14(5):305-14. doi: 10.1093/bfgp/elv014.

24.	Sarwal V, Niehus S, Ayyala R, Kim M, Sarkar A, Chang S, et al. A comprehensive benchmarking of WGS-based deletion structural variant callers. Brief Bioinform. 2022;23(4). doi: 10.1093/bib/bbac221. PubMed PMID: 35753701; PubMed Central PMCID: PMCPMC9294411.

25.	Cameron DL, Di Stefano L, Papenfuss AT. Comprehensive evaluation and characterisation of short read general-purpose structural variant calling software. Nature Communications. 2019;10(1):3240. doi: 10.1038/s41467-019-11146-4.

26.	Kosugi S, Momozawa Y, Liu X, Terao C, Kubo M, Kamatani Y. Comprehensive evaluation of structural variation detection algorithms for whole genome sequencing. Genome Biology. 2019;20(1):117. doi: 10.1186/s13059-019-1720-5.

27.	Gong T, Hayes VM, Chan EKF. Detection of somatic structural variants from short-read next-generation sequencing data. Briefings in Bioinformatics. 2020;22(3). doi: 10.1093/bib/bbaa056.

28.	Porubsky D, Ebert P, Audano PA, Vollger MR, Harvey WT, Marijon P, et al. Fully phased human genome assembly without parental data using single-cell strand sequencing and long reads. Nature Biotechnology. 2021;39(3):302-8. doi: 10.1038/s41587-020-0719-5.

29.	Cameron DL, Dong R, Papenfuss AT. StructuralVariantAnnotation: a R/Bioconductor foundation for a caller-agnostic structural variant software ecosystem. Bioinformatics. 2022;38(7):2046-8. doi: 10.1093/bioinformatics/btac042. PubMed PMID: 35134827; PubMed Central PMCID: PMCPMC8963309.

30.	Joe S, Park JL, Kim J, Kim S, Park JH, Yeo MK, et al. Comparison of structural variant callers for massive whole-genome sequence data. BMC Genomics. 2024;25(1):318. Epub 20240328. doi: 10.1186/s12864-024-10239-9. PubMed PMID: 38549092; PubMed Central PMCID: PMCPMC10976732.

31.	Zhuang G, Zhang X, Du W, Xu L, Ma J, Luo H, et al. A benchmarking framework for the accurate and cost-effective detection of clinically-relevant structural variants for cancer target identification and diagnosis. J Transl Med. 2024;22(1):65. Epub 20240116. doi: 10.1186/s12967-024-04865-w. PubMed PMID: 38229122; PubMed Central PMCID: PMCPMC10792779.

32.	van Belzen IAEM, Schönhuth A, Kemmeren P, Hehir-Kwa JY. Structural variant detection in cancer genomes: computational challenges and perspectives for precision oncology. npj Precision Oncology. 2021;5(1):15. doi: 10.1038/s41698-021-00155-6.

33.	Sudmant PH, Rausch T, Gardner EJ, Handsaker RE, Abyzov A, Huddleston J, et al. An integrated map of structural variation in 2,504 human genomes. Nature. 2015;526(7571):75-81. Epub 2015/10/04. doi: 10.1038/nature15394. PubMed PMID: 26432246; PubMed Central PMCID: PMCPMC4617611.

*34.	Li G, Jiang T, Li J, Wang Y. PanSVR: Pan-Genome Augmented Short Read Realignment for Sensitive Detection of Structural Variations. Front Genet. 2021;12:731515. Epub 20210819. doi: 10.3389/fgene.2021.731515. PubMed PMID: 34490049; PubMed Central PMCID: PMCPMC8417358.*

35.	Rajaby R, Liu D-X, Au CH, Cheung Y-T, Lau AYT, Yang Q-Y, Sung W-K. INSurVeyor: improving insertion calling from short read sequencing data. Nature Communications. 2023;14(1):3243. doi: 10.1038/s41467-023-38870-2.

*36.	Chiara M, Pesole G, Horner DS. SVM 2 : an improved paired-end-based tool for the detection of small genomic structural variations using high-throughput single-genome resequencing data. Nucleic Acids Research. 2012;40(18):e145-e. doi: 10.1093/nar/gks606.*

*37.	Michaelson JJ, Sebat J. forestSV: structural variant discovery through statistical learning. Nature Methods. 2012;9(8):819-21. doi: 10.1038/nmeth.2085.*

*38.	Kronenberg ZN, Osborne EJ, Cone KR, Kennedy BJ, Domyan ET, Shapiro MD, et al. Wham: Identifying Structural Variants of Biological Consequence. PLoS Comput Biol. 2015;11(12):e1004572. Epub 20151201. doi: 10.1371/journal.pcbi.1004572. PubMed PMID: 26625158; PubMed Central PMCID: PMCPMC4666669.*

*39.	Parikh H, Mohiyuddin M, Lam HYK, Iyer H, Chen D, Pratt M, et al. svclassify: a method to establish benchmark structural variant calls. BMC Genomics. 2016;17(1):64. doi: 10.1186/s12864-016-2366-2.*

*40.	Alzaid E, El Allali A, Aboalsamh H. A Classification Approach for Genome Structural Variations Detection. Journal of Proteomics & Bioinformatics. 2018;11:211-8.*

*41.	Bartenhagen C, Dugas M. Robust and exact structural variation detection with paired-end and soft-clipped alignments: SoftSV compared with eight algorithms. Brief Bioinform. 2016;17(1):51-62. Epub 20150520. doi: 10.1093/bib/bbv028. PubMed PMID: 25998133.*

*42.	Popic V, Rohlicek C, Cunial F, Hajirasouliha I, Meleshko D, Garimella K, Maheshwari A. Cue: a deep-learning framework for structural variant discovery and genotyping. Nature Methods. 2023;20(4):559-68. doi: 10.1038/s41592-023-01799-x.*

43.	Zhuang G, Zhang X, Du W, Xu L, Ma J, Luo H, et al. A benchmarking framework for the accurate and cost-effective detection of clinically-relevant structural variants for cancer target identification and diagnosis. Journal of Translational Medicine. 2024;22(1):65. doi: 10.1186/s12967-024-04865-w.

*44.	Sedlazeck FJ, Rescheneder P, Smolka M, Fang H, Nattestad M, von Haeseler A, Schatz MC. Accurate detection of complex structural variations using single-molecule sequencing. Nature Methods. 2018;15(6):461-8. doi: 10.1038/s41592-018-0001-7.*

*45.	Gaitán N, Duitama J. A graph clustering algorithm for detection and genotyping of structural variants from long reads. GigaScience. 2024;13. doi: 10.1093/gigascience/giad112.*

*46.	Jiang T, Liu Y, Jiang Y, Li J, Gao Y, Cui Z, et al. Long-read-based human genomic structural variation detection with cuteSV. Genome Biology. 2020;21(1):189. doi: 10.1186/s13059-020-02107-y.*

*47.	Mahmoud M, Gobet N, Cruz-Dávalos DI, Mounier N, Dessimoz C, Sedlazeck FJ. Structural variant calling: the long and the short of it. Genome Biol. 2019;20(1):246. Epub 20191120. doi: 10.1186/s13059-019-1828-7. PubMed PMID: 31747936; PubMed Central PMCID: PMCPMC6868818.*

*48.	Heller D, Vingron M. SVIM: structural variant identification using mapped long reads. Bioinformatics. 2019;35(17):2907-15. doi: 10.1093/bioinformatics/btz041. PubMed PMID: 30668829; PubMed Central PMCID: PMCPMC6735718.*

*49.	Zheng Y, Shang X. SVvalidation: A long-read-based validation method for genomic structural variation. PLoS One. 2024;19(1):e0291741. Epub 20240105. doi: 10.1371/journal.pone.0291741. PubMed PMID: 38181020; PubMed Central PMCID: PMCPMC10769053.*

*50.	Zhang Z, Jiang T, Li G, Cao S, Liu Y, Liu B, Wang Y. Kled: an ultra-fast and sensitive structural variant detection tool for long-read sequencing data. Brief Bioinform. 2024;25(2). doi: 10.1093/bib/bbae049. PubMed PMID: 38385878; PubMed Central PMCID: PMCPMC10883419.*

*51.	Hu H, Gao R, Gao W, Gao B, Jiang Z, Zhou M, et al. SVDF: enhancing structural variation detect from long-read sequencing via automatic filtering strategies. Brief Bioinform. 2024;25(4). doi: 10.1093/bib/bbae336. PubMed PMID: 38980375; PubMed Central PMCID: PMCPMC11232458.*

3. The manuscript primarily discusses tools published before 2020; however, significant advancements have been made in SV detection tools based on second-generation sequencing data in recent years. For instance, tools like "PanSVR: Pan-genome augmented short read realignment for sensitive detection of structural variations" and "Psi-Caller: a lightweight short read-based variant caller with high speed and accuracy" represent the latest research outcomes and should be discussed more thoroughly in the text. Additionally, the discussion on third-generation sequencing data is insufficient. It is advisable to incorporate discussions on the latest tools for SV detection using third-generation sequencing data, such as "SVDF: enhancing structural variation detection from long-read sequencing via automatic filtering strategies" and "Kled: an ultra-fast and sensitive structural variant detection tool for long-read sequencing data," to enrich and enhance the discussion section.

**Response:**

We sincerely thank the reviewer for the insightful comments and constructive suggestions. Following the recommendations, we have expanded our discussion to include recent advancements in SV detection tools based on short-read sequencing data. For instance, we now discuss "PanSVR," as recommended by the reviewer, which enhances SV calling by incorporating a pan-genome SV reference and read realignment, and "INSurVeyor," which improves the sensitivity of insertion detection from short-read sequencing data. These tools highlight opportunities to integrate algorithms optimized for low-complexity regions and specific SV types, enhancing overall detection accuracy.

Additionally, in response to the reviewer’s suggestion to incorporate a more comprehensive discussion on SV detection tools using third-generation sequencing data, we have included recent tools developed for long-read sequencing (LRS) data, such as "Svvalidator", "Kled” and "SVDF". Svvalidator" and "Kled" captures SV signatures from CIGAR strings and split reads to validate SVs effectively, while "SVDF" employs machine learning (ML)-based adaptive clustering and noise-filtering strategies to enhance SV detection. Integrating short-read and long-read sequencing provide opportunities for developing strategies tailored to diverse research and clinical needs.

Furthermore, in line with the editor’s comments, we have incorporated a discussion on additional tools that leverage ML and post-processing approaches, citing relevant works such as "doi: 10.4172/0974-276X.1000488, " as recommended by the editor. We now discuss "MPRClassify," which uses a multi-part read alignment strategy alongside three random forest classifiers to optimize detection for specific SV types, and "Cue," which transforms sequence alignments into images to highlight SV-related signals, using a convolutional neural network to predict the type, genotype, and genomic location of each SV. Additionally, we reference the work by Guiwu Zhuang et al., who developed a random-forest-based decision model to improve the precision of clinically relevant tumor-specific SV detection by evaluating multiple SV detection algorithms.

The discussion section has been thoroughly revised and now includes references to these relevant tools and studies. Adjustments to the reference list are noted in red in 'Revised Manuscript with Track Changes', as suggested. We appreciate the valuable comments.

***Page 23:***

***Discussion***

SVs can have a substantial phenotypic impact, disrupting gene function, regulation, or modifying gene dosage. Their significance in medicine and molecular biology has been underscored by recent studies [33]. The role of SVs in the pathogenicity of genetic diseases is increasingly recognized, and there is a possibility that screening for SVs may become routine in clinical settings in the future.

According to our study, relying on a single algorithm for SV detection may pose the risk of limited SV size and type detection, along with low F1 score. While our multiple agreement strategy, which focuses on consensus among algorithms, tends to yield higher precision at the cost of lower recall, employing a union combination enhanced recall and F1 scores for DELs compared to both single algorithms and the multiple agreement approach. However, most callers show poor F1 scores in INS detection due to low recall rates.  Specifically, DELLY (0.02) and GRIDSS (0.01) exhibited very poor F1 scores, while Manta (0.30) were the top performers among all non-commercial tools. The union strategy, which includes Manta, consequently achieved a comparable F1 score for INS detection, as illustrated in (Fig 6). This underscores the importance of choosing well-performing callers tailored to specific types and sizes of SVs for different clinical scenarios.

As for the concern of FP variants that may *could* be falsely related to a phenotype. We were particularly interested in FPs that are consistently detected by multiple callers. We analyzed the FPs called by at least two algorithms in the HG002 dataset and compared them with DRAGEN. We differentiated FPs identified only by the multi-agreement strategy (M), only by DRAGEN (D), and those identified by both (Both), the FP SV size displaying the median and interquartile range were shown as S4_figure. We found that the majority of FPs are smaller than 10^3 base pairs. For DEL FPs, the M group had a median SV length of 326 bp, while the D group had a median of 120 bp. As for INSs, due to the poor recall of most tools, the number of FPs was quite low, with D group INS FPs having a median SV length of 81 bp. Considering that most short reads range from 300 to 500 bp in length, we believe they should be capable of accurately sequencing variants of this small size. Each tool has its own underlying principles and parameters for detecting SVs, as outlined in their respective publications, many employ hard filters—such as thresholds for variant quality, and read depth—to reduce FPs. However, care is needed to balance filtering to avoid discarding true positives. In clinical practice, post-processing tasks like filtering target genes using a virtual panel, along with additional validation through the Integrative Genomics Viewer (IGV) or methods such as PCR, are commonly used. Therefore, weighing these considerations, we believe that achieving a high recall rate to minimize FN enhance*s* the utility of these findings in clinical settings.

Several studies *have* also shown that the occurrence of FPs in short-read sequencing for SV identification can be influenced by various factors, affecting overall precision. For example, Daniel L. Cameron et al. assessed the impact of sequence context and event size on the precision of 10 SV callers, evaluating these tools using simulation data and well-referenced cell lines. Their study included several tools that we also selected, such as Manta, DELLY, GRIDSS, and LUMPY. They identified several major factors *that* impact the precision: 1) The proximity of SNVs or indels near the SV breakpoints. When multiple flanking variants are present, false positive rates increase. 2) SVs located in low-complexity and simple tandem repeat regions are prone to higher false positive rates. SVs within LTR, LINEs, and SINEs regions also show reduced precision, leading to more false positives. 3) The size of the event also plays a role; they indicated SVs smaller than 100 bp are particularly challenging, whereas deletions between 300 to 500 bp tend to achieve higher precision.

*SV detection tools based on second-generation sequencing short read data have continued to advance in recent years. Recently, Gaoyang Li et al. published PanSVR [34], which enhances SV calling by incorporating a pan-genome SV reference and read re-alignment. By leveraging SNPs and INDELs in variable number tandem repeat (VNTR) and short tandem repeat (STR) regions, PanSVR improves alignment accuracy in these regions. Furthermore, with the integration of pan-genome reference information, PanSVR enhances recall for long insertions, showcasing the advantages of a pan-genome-based approach. In their benchmark study, PanSVR achieved higher F1 scores than Manta and Delly for detecting both DELs and INSs. Moreover,* Ramesh Rajaby and colleagues developed an INS caller, INSurVeyor [35], which improves the sensitivity of INS calling from short-read sequencing data, performing even better than Manta. This highlights opportunities to integrate better-performing algorithms tailored to low-complexity regions and specific SV types, like using *PanSVR* or INSurVeyor in combination with union strategies for enhanced performance.

*Additionally, research on applying supervised learning techniques for SV detection has been growing, with notable classification-based tools such as SVM2 [36], ForestSV [37], Wham [38], and Svclassify [39]. These tools employ distinct techniques and primarily utilize information extracted from RP signals to predict candidate SV regions. Recently, several machine learning (ML)-based approaches have demonstrated impressive performance. Eman A. Alzaid and colleagues developed MPRClassify [40], which uses a multi-part read alignment strategy with three independent random forest classifiers optimized to detect specific SV types in candidate SV regions. This approach relies solely on single reads, eliminating the need for paired-end read data. Experimental results indicated that single reads can effectively identify SVs without paired-end signatures, achieving performance comparable to existing methods, such as DELLY, Manta, and Softsv [41]. Victoria Popic et al. recently developed Cue [42], which transforms sequence alignments into images that highlight SV-related signals and utilizes a stacked hourglass convolutional neural network to predict the type, genotype, and genomic location of each SV. Evaluations on both synthetic and real short-read datasets demonstrated Cue's superior performance over established methods like Manta, DELLY, LUMPY, and SvABA, particularly in detecting a variety of SV classes, including DELs, DUPs, and INVs. In a*nother recent study, Guiwu Zhuang et al. [43] established an expert-reviewed tumor-specific clinically relevant SV call set, evaluating several algorithms for SV detection, and developed a random-forest-based decision model to improve the precision. Building on these research foundations, it is worth exploring the potential of integrating the union combination strategy with an ML*-*based decision model in the future. For practical clinical applications, further investigation into the performance using real clinical sample sets is necessary.

Finally, when addressing SV calling, the role of long-read sequencing cannot be overlooked. Long-read sequencing (*LRS*) offers remarkable advantages as it can span repetitive or other problematic regions. For applications that require identifying very long or complex SVs, performing a localized long-read de novo assembly is more effective than conventional short-read mapping approaches. Multiple studies have demonstrated that long-read sequencing technologies offer superior accuracy in SV detection compared to short-read approaches. Benchmarking results, as shown in recent studies [44-46], consistently highlight the advantages of long reads in terms of sensitivity and specificity, regardless of the specific tools used. Moreover, the recent advancements in long-read technologies and the ongoing reductions in sequencing costs—led by both Oxford Nanopore and PacBio—have made long-read sequencing increasingly accessible and widespread. *SV detection methods and post- processing approaches based on LRS have been developed to date, primarily relying on alignment-based methods that use manually designed heuristic rules tailored to sequencing platform characteristics [46-50]. For instance, Svvalidator [49] and Kled [50] both collect signatures from CIGAR strings and split reads to validate SVs, with Kled further employing an Omni Merging Algorithm (OMA) to refine and genotype clusters efficiently through multi-threading and optimized memory usage in C++. However, manually designed heuristics can introduce noise and false positives, a limitation that ML-based approaches like SVDF [51] addressed by employing adaptive clustering and noise-filtering strategies.* Integrating short-read and long-read sequencing provide*s* opportunities for developing strategies tailored to diverse research and clinical needs.

Although our study only focused on the DELs and INSs— which are the majority but relatively simple SV types in the human genome— methods failing to detect DELs and INSs might also be less effective at identifying more complex SV types. Currently, our study evaluated the ability of various methods and combination strategies to detect DELs and INSs, providing a valuable and optimistic perspective on overall SV detection performance.

***Page 31:***

***References***

*34. Li G, Jiang T, Li J, Wang Y. PanSVR: Pan-Genome Augmented Short Read Realignment for Sensitive Detection of Structural Variations. Front Genet. 2021;12:731515. Epub 20210819. doi: 10.3389/fgene.2021.731515. PubMed PMID: 34490049; PubMed Central PMCID: PMCPMC8417358.*

*35.	Rajaby R, Liu D-X, Au CH, Cheung Y-T, Lau AYT, Yang Q-Y, Sung W-K. INSurVeyor: improving insertion calling from short read sequencing data. Nature Communications. 2023;14(1):3243. doi: 10.1038/s41467-023-38870-2.*

*36.	Chiara M, Pesole G, Horner DS. SVM 2 : an improved paired-end-based tool for the detection of small genomic structural variations using high-throughput single-genome resequencing data. Nucleic Acids Research. 2012;40(18):e145-e. doi: 10.1093/nar/gks606.*

*37.	Michaelson JJ, Sebat J. forestSV: structural variant discovery through statistical learning. Nature Methods. 2012;9(8):819-21. doi: 10.1038/nmeth.2085.*

*38.	Kronenberg ZN, Osborne EJ, Cone KR, Kennedy BJ, Domyan ET, Shapiro MD, et al. Wham: Identifying Structural Variants of Biological Consequence. PLoS Comput Biol. 2015;11(12):e1004572. Epub 20151201. doi: 10.1371/journal.pcbi.1004572. PubMed PMID: 26625158; PubMed Central PMCID: PMCPMC4666669.*

*39.	Parikh H, Mohiyuddin M, Lam HYK, Iyer H, Chen D, Pratt M, et al. svclassify: a method to establish benchmark structural variant calls. BMC Genomics. 2016;17(1):64. doi: 10.1186/s12864-016-2366-2.*

*40.	Alzaid E, El Allali A, Aboalsamh H. A Classification Approach for Genome Structural Variations Detection. Journal of Proteomics & Bioinformatics. 2018;11:211-8.*

*41.	Bartenhagen C, Dugas M. Robust and exact structural variation detection with paired-end and soft-clipped alignments: SoftSV compared with eight algorithms. Brief Bioinform. 2016;17(1):51-62. Epub 20150520. doi: 10.1093/bib/bbv028. PubMed PMID: 25998133.*

*42.	Popic V, Rohlicek C, Cunial F, Hajirasouliha I, Meleshko D, Garimella K, Maheshwari A. Cue: a deep-learning framework for structural variant discovery and genotyping. Nature Methods. 2023;20(4):559-68. doi: 10.1038/s41592-023-01799-x.*

*43.	Zhuang G, Zhang X, Du W, Xu L, Ma J, Luo H, et al. A benchmarking framework for the accurate and cost-effective detection of clinically-relevant structural variants for cancer target identification and diagnosis. Journal of Translational Medicine. 2024;22(1):65. doi: 10.1186/s12967-024-04865-w.*

*44.	Sedlazeck FJ, Rescheneder P, Smolka M, Fang H, Nattestad M, von Haeseler A, Schatz MC. Accurate detection of complex structural variations using single-molecule sequencing. Nature Methods. 2018;15(6):461-8. doi: 10.1038/s41592-018-0001-7.*

*45.	Gaitán N, Duitama J. A graph clustering algorithm for detection and genotyping of structural variants from long reads. GigaScience. 2024;13. doi: 10.1093/gigascience/giad112.*

*46.	Jiang T, Liu Y, Jiang Y, Li J, Gao Y, Cui Z, et al. Long-read-based human genomic structural variation detection with cuteSV. Genome Biology. 2020;21(1):189. doi: 10.1186/s13059-020-02107-y.*

*47.	Mahmoud M, Gobet N, Cruz-Dávalos DI, Mounier N, Dessimoz C, Sedlazeck FJ. Structural variant calling: the long and the short of it. Genome Biol. 2019;20(1):246. Epub 20191120. doi: 10.1186/s13059-019-1828-7. PubMed PMID: 31747936; PubMed Central PMCID: PMCPMC6868818.*

*48.	Heller D, Vingron M. SVIM: structural variant identification using mapped long reads. Bioinformatics. 2019;35(17):2907-15. doi: 10.1093/bioinformatics/btz041. PubMed PMID: 30668829; PubMed Central PMCID: PMCPMC6735718.*

*49.	Zheng Y, Shang X. SVvalidation: A long-read-based validation method for genomic structural variation. PLoS One. 2024;19(1):e0291741. Epub 20240105. doi: 10.1371/journal.pone.0291741. PubMed PMID: 38181020; PubMed Central PMCID: PMCPMC10769053.*

*50.	Zhang Z, Jiang T, Li G, Cao S, Liu Y, Liu B, Wang Y. Kled: an ultra-fast and sensitive structural variant detection tool for long-read sequencing data. Brief Bioinform. 2024;25(2). doi: 10.1093/bib/bbae049. PubMed PMID: 38385878; PubMed Central PMCID: PMCPMC10883419.*

*51.	Hu H, Gao R, Gao W, Gao B, Jiang Z, Zhou M, et al. SVDF: enhancing structural variation detect from long-read sequencing via automatic filtering strategies. Brief Bioinform. 2024;25(4). doi: 10.1093/bib/bbae336. PubMed PMID: 38980375; PubMed Central PMCID: PMCPMC11232458.*
